# A Predictive Strategy Based on Volatile Profile and Chemometric Analysis for Traceability and Authenticity of Sugarcane Honey on the Global Market

**DOI:** 10.3390/foods10071559

**Published:** 2021-07-05

**Authors:** Pedro Silva, Jorge Freitas, Fernando M. Nunes, José S. Câmara

**Affiliations:** 1 CQM—Centro de Química da Madeira, Campus da Penteada, Universidade da Madeira, 9020-105 Funchal, Portugal; pedro_dasilva@hotmail.com (P.S.); Jorge.freitas@staff.uma.pt (J.F.); 2 CQ-VR—Centro de Química-Vila Real, Food and Wine Chemistry Lab., Departamento de Química, Universidade de Trás-os-Montes e Alto Douro, 5001-801 Vila Real, Portugal; fnunes@utad.pt; 3 Departamento de Química, Faculdade de Ciências Exactas e Engenharia, Campus da Penteada, Universidade da Madeira, 9020-105 Funchal, Portugal

**Keywords:** sugarcane syrup, volatile profile, authenticity, geographical origin

## Abstract

Sugarcane honey (SCH) is a syrup produced on Madeira Island and recognized by its unique aroma, a complex attribute of quality with an important influence on the final consumer’s acceptance of the product, and determined by a complex mixture of a large number of volatile organic compounds (VOCs) generated during its traditional making process and storage. Therefore, the purpose of this study was to establish the volatile profile of genuine SCH produced by a regional certified producer for seven years and compare it with syrups from non-certified regional producers and with producers from different geographical regions (Spain, Egypt, Brazil and Australia), as a powerful strategy to define the volatomic fingerprint of SCH. Different volatile profiles were recognized for all samples, with 166 VOCs being identified belonging to different chemical classes, including furans, ketones, carboxylic acids, aldehydes and alcohols. Chemometric analysis allowed (i) the differentiation between all syrups, being more pronounced between SCH and other syrups; and (ii) the identification of 32 VOCs as potential markers for the traceability and authenticity of SCH on the global market.

## 1. Introduction

Food authenticity has become a critical issue due to the high globalization of the food trade, leading to an unprecedented diversity of food products on the market, and consequently, to an increasing occurrence of food fraud [1]. Food products with a high added-value, typically exclusive to certain regions or obtained from traditional processes, are the most desirable targets for counterfeiters [2]. In this context, the European Union (EU) promotes three types of authenticity certification for regional or traditional food products: (i) Protected Designation of Origin (PDO); (ii) Protected Geographical Indication (PGI); (iii) Traditional Speciality Guaranteed (TSG). On the one hand, it guarantees a greater appreciation of the product on the market, increasing profitability for producers, as this type of certification is widely attractive to both producers and consumers. On the other hand, it is a guarantee of the quality expected by consumers and, mainly, a guarantee of food security. However, the application process for each of these three types of EU certification is long, time-consuming and exhaustive; every step of the regulatory framework (Regulation N° 1151/2012) must be meticulously followed before submission to the European Commission in order to guarantee the right to use the respective label of certification [2,3,4]. To date, hundreds of EU certification applications have been accepted, including several food products (i.e., wine, vinegar, olive oil, cheese, among others), protecting the local producers and traditional practices.

Recently, the government of Madeira Island, Portugal, has started the process of authentication to protect one of its most valuable traditional food products, the sugarcane syrup (SCH). The SCH is a crystalline black syrup produced from the stalks of fresh sugarcane (*Saccharum officinarum* L.) cultivated under the mild climate conditions of the Atlantic region, and recognized worldwide for its excellent quality and sui generis organoleptic properties. The distinctive and unique properties of SCH arise from the use of sugarcane cultivars grown in the region and, principally, from secular and traditional processing and storage conditions in addition to terroir (climatic conditions and cultivation treatments). To distinguish the SCH from other syrups, molasses and treacles, the government created a regional production certification brand. Nevertheless, its importance and economic value have led to the emergence of adulterated SCH with low-quality sugarcane-derived products from different geographical origins, which has affected its notoriety [5,6]. In this context, it becomes essential for the identification of potential molecular markers to guarantee its typicality and authenticity and, consequently, its traceability on the market, thereby supporting a potential application for EU certification.

One of the most recent and promising developments in the food authentication domain is Foodomics, which emerged as a new approach supported by high resolution- and MS-based techniques to solve some of the new challenges for global food safety [7,8,9]. In SCH, the chemical complexity of VOCs formation and origin can be valuable for the establishment of its typicality and authenticity. The traditional conditions of processing and storage, together with the exclusive use of sugarcanes from authorized varieties, cultivated on Madeira Island, can generate a specific “fingerprint” of the volatile pattern. Furthermore, this approach has been used for the EU certification of several food products, such as Modena Balsamic Vinegar from Italy [10], “La Rioja” Olive Oil from Spain [11], “Corsica” Honey from France [12] and Madeira wine from Portugal [13].

In this context, the purpose of this study was to establish the volatile profile of sugarcane-based syrup produced by a regional certified producer analyzing seven processing years (2007, and 2013 to 2018) in order to determine the typicality and authenticity of genuine SCH. Additionally, the volatile profile of sugarcane-based syrups from non-certified regional producers and from different geographic regions (Spain, Egypt, Brazil and Australia), was established. Subsequently, chemometric analysis was applied to the obtained data allowing the identification of a set of predictive VOCs as potential traceability markers of genuine SCH on the market. The predictive strategies based on MS techniques combined with chemometric analysis have been successfully applied in the traceability of food products [14,15,16]. Solid-phase microextraction in headspace mode (HS-SPME) with gas chromatography–mass spectrometry (GC-MS) methodology was performed according with our previous study [5], being used to establish the volatile profile of all samples. The proposed predictive strategy will represent a valuable tool to guarantee the traceability of genuine SCH, and to support a potential application for EU certification of its geographical origin.

## 2. Materials and Methods

### 2.1. Standards, Reagents, Materials and Software

Internal standard (IS), 4-heptanone, was purchased from Sigma-Aldrich (St. Louis, MO, USA). Sodium chloride was acquired from Panreac (Barcelona, Spain). SPME holder and the fiber DVB/CAR/PDMS (50/30 μm) were acquired from Supelco (Bellefonte, PE, USA). The BP-20 fused silica capillary column (60 m × 0.25 mm I.D. × 0.25 µm) was acquired from SGE (Dortmund, Germany). Ultrapure deionized water (H_2_O) was obtained from a system from Millipore (Burlington, MA, USA). The Mixer was purchased from Thermo Scientific (Burlington, MA, USA). All methods used in chemometric analysis were performed using the STATSOFT STATISTICA 12.0 (2013) software (Tulsa, OK, USA).

### 2.2. Samples

Samples from the traditional and certified producer Fábrica de Mel-de Cana do Ribeiro Sêco (FRS), Madeira Island, Portugal, were collected from lots subsequently placed on the market in 2007 (FRS07), 2013 (FRS13), 2014 (FRS14), 2015 (FRS15), 2016 (FRS16), 2017 (FRS17) and 2018 (FRS18). All other sugarcane-based syrups samples were purchased on the regional market between 2014 and 2018, while the samples from the non-certified regional producers (ECAL14 and NCAL14) and regional homemade producer (GLA14), were obtained in 2014. Samples from Brazil (MDBR14 and MCBR14) were from the 2014 harvest, whereas the samples from Spain (ESP16), Egypt (EGPA16, EGPB16, EGPC16 and EGP17) and Australia (AUS17) were from the 2016 and 2017 harvest, respectively. All samples were stored under stable conditions (4 °C, in the dark). Identification (ID) replicate number, replicate code, sample code, group code, processing year, processing type, geographic origin and regional certification are described in Supplementary Material (S) Table S1.

### 2.3. Solid-Phase Microextraction Procedure

The extraction of VOCs from SCH samples was carried out by HS-SPME developed, optimized and validated in our previous study [5]. Briefly, the samples were prepared every day by addition of sample (15 g) into H_2_O (10 mL) in a ratio 3:2 (*w*/*v*), being homogenized for 1 min in a vortex mixer, and aliquoted (8 mL) and stored at 4 °C. After, these aliquots were placed into a glass vial containing NaCl (60 mg) in a thermostatic bath at 30 °C for 5 min. The HS-SPME was achieved for 60 min at 30 °C with magnetic agitation. Every day, the fiber was cleaned for 15 min at 250 °C in GC-MS, being performed blank assays. All samples were completed in triplicate experiments.

### 2.4. Gas Chromatography-Mass Spectrometry Analysis

The analysis was carried on 6890N Network GC system with a 5975 quadrupole MS detector, both acquired from Agilent Technologies (Santa Clara, CA, USA). A GC fused silica capillary column BP-20 from SGE was used, being acquired from Thermo Scientific (Burlington, MA, USA). The GC protocol for the column oven was: started at 40 °C, then 2 min hold, after it was increased (0.25 °C min^−1^) to 45 °C, then a 2 min hold, subsequently it was increased (4 °C min^−1^) up to 70 °C, another 2 min hold, once again it was increased (3 °C min^−1^) to 130 °C, another 2 min hold, and finally it was increased (3 °C min^−1^) to 220 °C, then a final 7 min hold, resulting in 91.25 min total time. The column flow was 1.0 mL min^−1^ using a carrier gas He (99.999%) from Air Liquid (Lisbon, Portugal). The injection GC port was worked in the splitless mode at 250 °C. In MS system, the temperatures of the transfer line, quadrupole and ionization source were 270, 150 and 230 °C, respectively. Electron impact mass spectra were recorded at 70 eV voltage and at 10 µA current. The MS acquisitions were performed in full-scan mode (30–300 *m*/*z*). The identification VOCs method was performed by the Agilent MS ChemStation Software with a NIST14 Mass Spectral Library (2014). The VOCs’ identification was successfully achieved with a similarity higher than 75%. The total peak area values were accomplished by target ion semi-quantification method. The results are presented as relative peak areas (RPA), being achieved by the ratio of each VOC peak area value by the IS peak area value.

### 2.5. Chemometric Analysis

The chemometric analysis procedure was performed according to the developed procedure in our previous study [17]. Briefly, all samples were grouped in one of four groups formed according to their geographic origin. The FRS07, FRS13, FRS14, FRS15, FRS16, FRS17 and FRS18 samples were classified into the regional certified producer group (CERT); the ECAL14, NCAL14 and GLA14 samples into the regional non-certified producers group (NCERT); the ESP16, EGP17, EGPA16, EGPB16 and EGPC16 samples into the Mediterranean region producers group (MED); the MDBR14, MCBR14 and AUS17 samples into the southern hemisphere region producers group (STH). The one-way ANOVA with Tukey’s post-hoc test, principal component analysis (PCA), partial least square (PLS), linear discriminant analysis (LDA) and hierarchical cluster analysis (HCA) were fully described in our previous study [17].

## 3. Results and Discussion

### 3.1. Establishment of the Volatile Profile from Sugarcane-Based Syrups

The establishment of the VOC profiles of 18 sugarcane-based syrups from different geographical origins was successfully achieved by the HS-SPME/GC-MS method. The information about the 166 identified VOCs is listed in Table S2. The mean RPA and RSD values of the VOCs are described in Table S3, the mean, minimum and maximum values of RPA for the VOCs are summarized in Table S4. The representative GC-MS chromatograms for each sample under analysis is shown in Figure S1.

#### 3.1.1. Number of Identified Volatile Organic Compounds

The analysis of the volatile profile of all samples allowed for the identification and semi-quantitation of 166 VOCs from a wide diversity of chemical classes. One hundred and nineteen VOCs (71.69%) were identified in all samples under analysis. On the contrary, only six VOCs (3.61%) were identified in one specific sample, namely: 2-heptanone (HPT2ONE) in ECAL14; ethyl hexanoate (EESTHA), ethyl octanoate (EESTOA) and ethyl decanoate (EESTDA) in GLA14; 2-ethyl-5-methyl-pyrazine (E5MPYZNE) and trimethyl-pyrazine (TMPYZNE) in AUS17. Interestingly, 48 VOCs (28.92%) were also previously identified in sugarcane-based syrups from other geographical origins, 17 VOCs in syrup from Egypt [18], 14 VOCs in syrup from the USA [19], 12 VOCs in syrup from the Dominican Republic [20], 11 VOCs in syrup from China [21] and 15 VOCs in syrups from Japan [22,23] (Table S5).

#### 3.1.2. Main Volatile Organic Compounds

**Figure 1:**
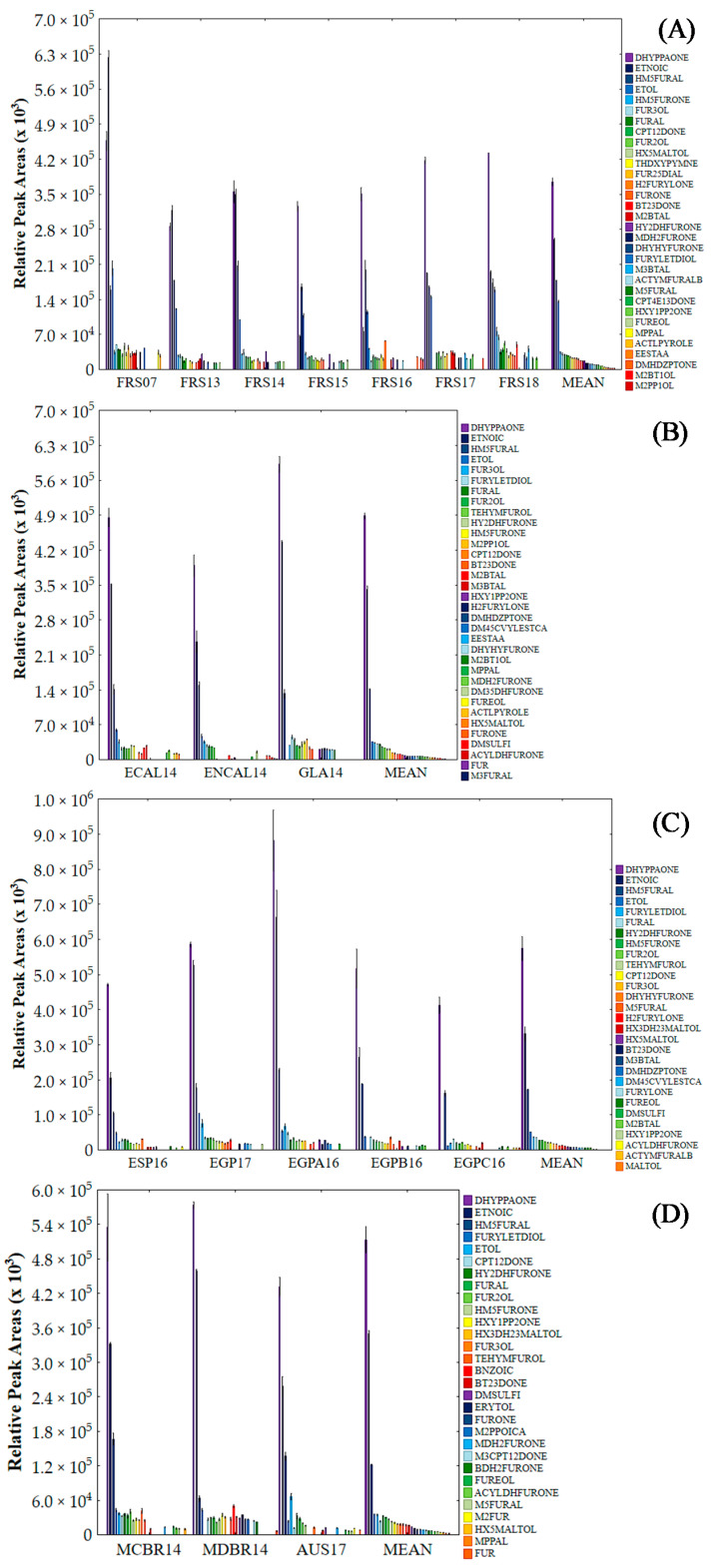
The relative peak area values of the 20 main VOCs for samples of CERT group (**A**), NCERT group (**B**), MED group (**C**) and STH group (**D**).

Although the contribution of each of the 166 VOCs was important to establish the SCH volatile profile, their RPA (×10^3^) values varied between 0.5 and 1 × 10^6^. The 20 major VOCs with higher contribution for the volatile profile of investigated samples are described in Figure 1A–D.

1,3-Dihydroxy-2-propanone (DHYPPAONE), 5-(hydroxymethyl)-2-furfural (HM5FURAL), furfural (FURAL) and 2-furanmethanol (FUR2OL) were common to all samples. Furthermore, DHYPPAONE was the most dominant VOC for 17 samples, second only on the FRS07 sample, followed by HM5FURAL. The high contribution of these VOCs is expected because both are strongly linked to the thermal processing of SCH (i.e., sugars, amino acids), being well-known markers of the occurrence of non-enzymatic browning reactions, such as the Maillard reaction, Strecker reaction and caramelization [6,24]. 3,5-Dimethyl-dihydro-2-furanone (DM35DHFURONE) and 3-methyl-furfural (M3FURAL) were dominant in ENCAL14; maltol (MALTOL), benzoic acid (BNZOIC), erythritol (ERYTOL), 2-methyl-propanoic acid (M2PPOICA), 3-methyl-1,2-cyclopentanedione (M3CPT12DONE) and 5-butyl-dihydro-2(3H)-furanone (BDH2FURONE) were dominant in MDBR14; 2-methyl-furan (M2FUR) was dominant in AUS17. In fact, most of the VOCs classified as main contributors to the volatile profile are commonly linked to high temperatures used in the processing of sugarcane-based syrups, indicating that processing is critical for the establishment of its volatile profile.

#### 3.1.3. Chemical Class Classification of Volatile Organic Compounds

**Figure 2:**
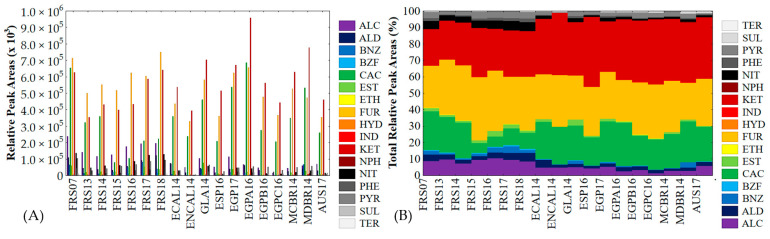
The contribution, relative peak area (**A**) and % total relative peak area (**B**) values of each chemical class group for all samples.

The sum of VOCs, RPA and TRPA values of each chemical class recognized in sugarcane-based samples are summarized in Table S6A–D, respectively. The contribution, RPA and TRPA values, of each chemical class to the volatile profile of all samples are shown in Figure 2A,B, respectively.

Seventeen chemical classes were recognized in SCH’s volatile profile: alcohols (ALC), aldehydes (ALD), benzenes (BNZ), benzofurans (BZF), carboxylic acids (CAC), esters (EST), ethers (ETH), furans (FUR), hydrocarbons (HYD), indenes (IND), ketones (KET), naphthalenes (NPH), nitrogen (NIT), phenols (PHE), pyrans (PYR), sulfur (SUL) and terpenoids (TER). All chemical classes were identified in all sugarcane-based syrups. Interestingly, FUR was the chemical class with the highest contribution to the volatile profile of genuine SCH obtained by the certified producer. For the remaining samples, FUR was the second main class for ECAL14, NCAL14, GLA14, ESP16, EGP17, EGPB16, EGPC16, MCBR14 and AUS17, being the third main class for EGPA16 and MDBR14, where its contribution was higher than 22%. In addition, FUR was the class with the highest number of VOCs (44). HM5FURAL was the VOC with the highest influence in FUR class contribution followed by FURAL, FUR2OL and 3-furanmethanol (FUR3OL). KET was the main class for most of the samples with a contribution higher than 30%, but only the second main contributor for samples from the certified producer, with the exception of the FRS07 samples, where it was the third main contributor. DHYPPAONE was undoubtedly the most dominant from the KET class, being responsible for more than 50% of the total contribution of this chemical class for the total volatile profile. Additionally, 1-hydroxy-2-propanone (HXY1PP2ONE), 1,2-cyclopentanedione (CPT12DONE) and 2,3-butanedione (BT23DONE) had a significant contribution to the KET class, but to a minor level. CAC presented a high contribution to the volatile profile of all samples ranging from 5.97% (FRS15) to 26.26% (EGP16A). Among the nine VOCs classified in the CAC class, ethanoic acid (ETNOIC) was responsible for more than 90% of the total volatile fraction. In fact, FUR, KET and CAC classes comprised a large fraction of the volatile profile of all samples analyzed, from 66.92% (FRS17) to 92.07% (ENCAL14). ALC and NIT classes also had a substantial contribution to the volatile profile of investigated samples, being more pronounced in samples from the certified producer. The contributions of ALC and NIT for certified samples varied between 7.14–10.39% and 3.18–5.88%, respectively. For the remaining samples, the contributions of ALC and NIT ranged between 1.27–5.67% and 0.17–2.59%, respectively. Ethanol (ETOL) was the highest contributor for the ALC class, followed by 2-methyl-1-propanol (M2PP1OL) and 2-cyclohexenol (CHEX2E1OL). Among the 16 VOCs assigned in the NIT class, 2,4,6-trihydroxypyrimidine (THDXYPYMNE), 2-acetylpyrrole (ACTLPYROLE) and 4-pyridinol (PYRDINOL) were the VOCs with the highest contribution. Likewise, ALD, EST and PYR classes had a reasonable contribution (>1%) for all samples from the certified producer, being less expressive for most of the other samples. Typically, BNZ and PHE classes also presented a higher contribution for the volatile profile of certified samples compared to non-certified samples. The number of VOCs classified into each of these chemical classes were: ALD (9), EST (11), PYR (5), BNZ (13) and PHE (8). ALD contribution was mainly explained by 2-methyl-propanal (MPPAL), 2-methyl-butanal (M2BTAL) and 3-methyl-butanal (M3BTAL), while EST contribution was mostly influenced by vinylene carbonate (VYLESTCA), 4,5-dimethyl vinylene carbonate (DM45CVYLESTCA) and ethyl acetate (EESTAA). PYR contribution was predominantly influenced by MALTOL, and its derivatives, 5-hydroxy-maltol (HX5MALTOL) and 3-hydroxy-2,3-dihydro-maltol (HX3DH23MALTOL). BNZ and PHE contributions were highly influenced by benzeneacetaldehyde (BENZACETAL) and 3-methoxy-1,2-benzenediol (M3BNZDIOL); phloroglucinol (PHLOGLNOL) and phenol (PHEOL), respectively. VOCs from BZF, ETH, HYD, NPH, SUL and TER were identified in all samples, but their contribution was normally lower than 1% and the number of VOCs assigned was always lower than five.

Most of the chemical classes found in the volatile profiles of sugarcane-based syrups, such as FUR, BZF, PHE and PYR and, in a minor way, ALD, BNZ, KET, NPH and NIT, are highly related to thermal reactions (i.e., Maillard reaction, Strecker degradation and caramelization) that occurs during the processing of sugarcane [25,26,27]. Alternatively, ALD, KET and NIT classes can also be originated from enzymatic reactions and microbial activity in the sugarcane before processing or during the syrup’s storage. Additionally, ALC, CAC, EST and SUL classes are commonly associated with enzymatic and microbial activity [28,29,30]. Other classes, namely HYD and TER, are probably products from biochemistry pathways that occur in sugarcane plants [31]. The formation and origin of VOCs from the IND and ETH classes are more difficult to establish, being the result of probable crop contamination by biomass burning, plastic residues combustion and pesticide application [32,33].

### 3.2. Chemometric Analysis Based on the Volatile Profile of Sugarcane-Based Syrups

Chemometric analysis was applied to the VOCs’ dataset to obtain a predictive strategy that guarantees the traceability of genuine SCH on the global market. Predictive strategies have been successfully applied for the traceability of food products such as olive oil [34], coffee [35] and cider [36].

#### 3.2.1. One-Way ANOVA Test

One-way ANOVA with post-hoc Tukey test results (*p* and *F* values) are described in Table S7. The assigned group for each sample is described in Table S1.

The results show that 147 VOCs (86.75%) presented statistically significant differences in RPA values between all 18 samples under analysis. On the other hand, 19 VOCs (13.25%) did not show statistically significant differences, being removed from the further analysis. Moreover, 53 VOCs (31.93%) showed high differences, with *F* values ≥ 10 between all samples, and among these, 19 VOCs (11.45%) demonstrated huge significant differences, with *F* values ≥ 20. The post-hoc Tukey test results demonstrated a high level of dissimilarity in volatile profiles between all groups. The CERT group showed the highest dissimilarity from the other groups, which presented 47 VOCs (28.31%) with statistically significant differences for the NCERT, MED and SYH groups, simultaneously. In a minor level of dissimilarity, the STH group presented 22 VOCs (13.25%), followed by the NCERT group with 12 VOCs (7.23%) and the MED group with only two VOCs (1.20%). Interestingly, most of these VOCs only presented significant differences for one specific group and irrespective of all combinations with other groups.

#### 3.2.2. Principal Component Analysis and Partial Least Squares

**Figure 3:**
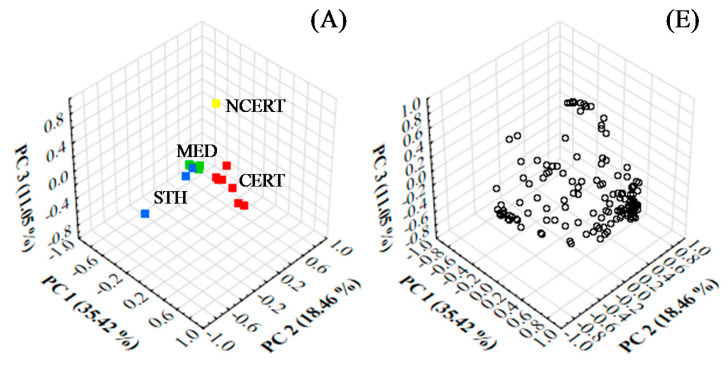

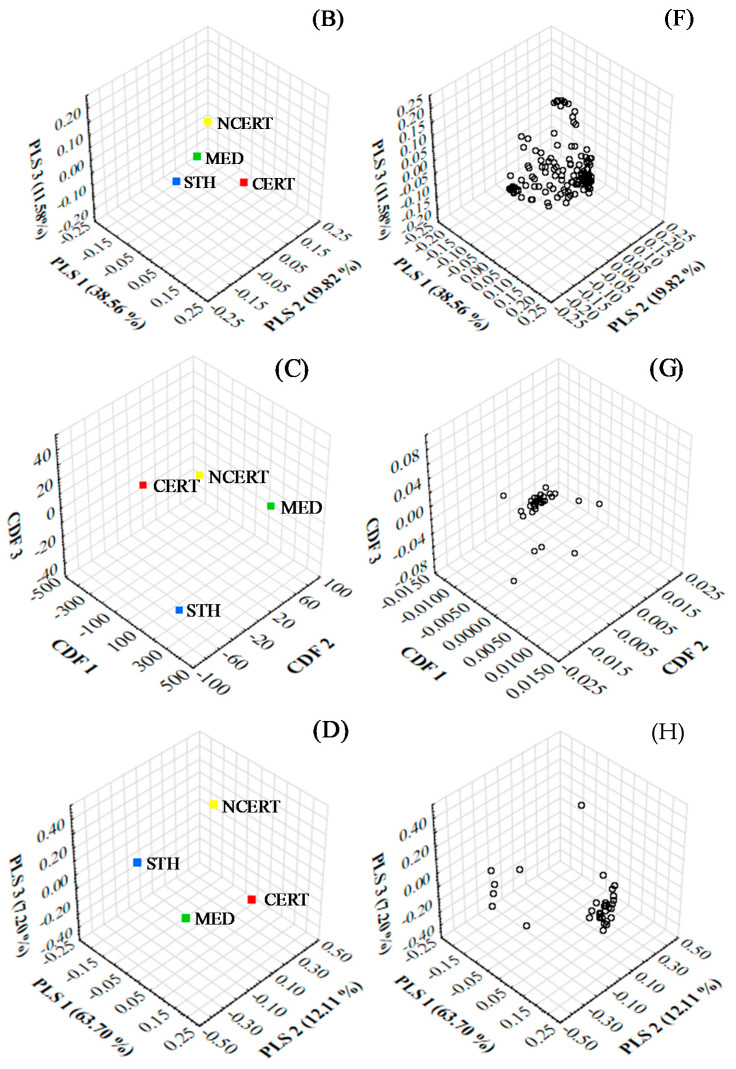
The loading 3D plot of all samples (**A**) and the selected 144 VOCs (**E**) for PCA, all centroids (**B**) and the selected 144 VOCs (**F**) for PLS, all centroids (**C**) and the 32 most predictive VOCs (**G**) for LDA, and all centroids (**D**) and the 32 most predictive VOCs (**H**) for PLS.

The PCA and PLS analyses were performed on 147 VOCs that showed statistically significant differences in the ANOVA test. The loading values and VIP scores for identified VOCs are summarized in Table S9. The loading values of 18 samples and four centroids are listed in Table S10. The scores values of samples are summarized in Table S11. The PCA line plot based on loading values of samples for the three main components are presented in Figure S2A–C, respectively. The PLS line plot based on loading values of four centroids for the three main components are shown in Figure S2D–F. The PCA 3D plots based on loading values of all samples and the VOCs for the three main components are shown in Figure 3A,E, respectively. The PLS 3D plots based on loading values of the four centroids and the VOCs for the three main components are shown in Figure 3B,F, respectively.

The three main components of PCA comprised 64.92% of the total variance (TVA). The projection of structure based on loading results from the three main components demonstrated a clear differentiation between the samples from the certified producer and samples from the other producers. In the PC1 projection (35.42% TVA), all samples from the certified producer showed a high variance from the remaining samples under analysis. In the PC2 projection (18.46%), the samples from Madeira Island, including certified and non-certified producers, presented a slight variance from other geographical regions’ samples. Also, in PC2 projection, the MDBR14 sample showed a high variance from all remaining samples, while in the PC3 projection (11.05%), a high variance was shown between the sample from the homemade producer (GLA14) and all other samples.

In PLS the samples were classified according to the type of producer and geographical localization, being classified as: centroid-CERT (C-CERT), centroid-NCERT (C-NCERT), centroid-MED (C-MED) and centroid-STH (C-STH). The three main components of PLS analysis (PLS1, PLS2 and PLS3) were responsible for 69.96% of TVA, the sum of all 18 components comprised 99.66%. Interestingly, the results from PLS1, PLS2 and PLS3 demonstrated that all group centroids were clearly separated. In PLS1 projection (38.56%), a high variance was obtained between C-CERT and the other centroids. For PLS2 projection (19.82%) a substantial and equitable differentiation was observed between all centroids. In PLS3 projection (11.58%), a higher variance was shown between C-NCERT and the other centroids.

PCA and PLS results were based on 144 VOCs, where each one influenced the projection of samples and centroids differently. In the case of PLS projection, it was possible to identify the individual contribution of each VOC for the projection structure.

The PLS 3D plot based on loading values for all 147 VOCs exposed the fact that a significant number of VOCs influenced the projection of C-CERT, mainly belonging to ALD, ALC, FUR and NIT chemical classes. The projection of NCERT was very influenced by VOCs from EST and BNZ classes, such as EESTOA, EESTDA, ethyl undecanoate (EESTUNDA), benzyl acetate (PMESTAA) and benzeneethanol (BENZETOL), while the projection of STH was influenced principally by VOCs from CAC, IND and NPH, such as heptanoic acid (HPTOIC), octanoic acid (OCTOIC), decanoic acid (DECOIC), 2,3-dihydro-1,1,4,6-tetramethyl-1H-indene (DHT1146MIDNE), and 2,3-dihydro-1,1,5,6-tetramethyl-1H-indene (DHT1156MIDNE). Lastly, the projection of C-MED was influenced by a lower number of VOCs than the other three centroids, they were dimethyl disulfide (DMDSFD), HPT2ONE, E5MPYZNE, TMPYZNE and M3FURAL.

#### 3.2.3. Linear Discriminant Analysis

The procedure applied to reduce the matrix dimension based on the VIP scores showed unsatisfactory results in the PLS and HCA analysis, not being used for further analysis. The LDA information and respective PLS and HCA plots constructed according to VIP scores are described in Table S14 and Figure S5, respectively. The LDA information of the selected 32 VOCs according to the *F* values from the ANOVA test are described in Table 1. The canonical discriminant functions (CDF) coefficients and higher probability classification results of all samples are described in Table S13. The LDA 3D plots based on CDF coefficients of the four centroids and respective VOCs for the three main components are shown in Figure 3C,G, respectively.

Only furfuryl acetate was removed from LDA analysis throughout all 22 steps (backward selection with *p* < 0.05 to enter and remove). Thus, LDA results are described in Table S2. All 54 replicates obtained from 18 syrup samples were classified at a 100% correct rate.

The projection of LDA results based on the three main CDFs presented in Figure 3c demonstrated a high level of discrimination between all four centroids. In CDF 1, a discrimination was verified between the C-CERT and the other centroids. In CDF 2, a high discrimination was observed between all centroids, where it verified a proximity between the centroids based on syrups from Madeira Island (C-CERT and C-NCERT) compared to centroids formed with samples from foreign syrups (C-MED and C-STH). Likewise, in CDF3 a higher discrimination was observed among the four centroids, being more prominent between the C-NCERT and the remaining three centroids. The LDA results evidenced that it was possible to discriminate and classify correctly all syrup samples based on only 32 VOCs.

#### 3.2.4. Partial Least Squares and Hierarchical Clustering Analysis

An additional PLS was completed to validate the projection structure between all syrup samples based only on the 32 most predictive VOCs (Table S14). The loading values and VIP scores of PLS for each VOC are summarized in Table 1. The loading values of four centroids and the scores of 18 samples are summarized in Table S13, while the PLS line plots based on the loading values of four centroids according to the three main components are shown in Figure S4A–C, respectively. The PLS line plot based on the loading values of the previously selected 32 VOCs for the three main components are presented in Figure S4D–F, respectively. The PLS 3D plots based on the loading values of the four centroids and 32 VOCs for the three main components are presented in Figure 3D,H, respectively.

The PLS analysis performed according to the three main components explained 83.00% of TVA and the summary of all 18 components described 99.88%. As predicted, the results shown in the PLS loading 3D plot revealed that the four centroids were categorically separated, whereas a higher and equitable variance between all centroids was verified. Likewise to CDF1, the projection of PLS1 (63.70%) showed a higher variance between the C-CERT and the other three centroids. In PLS2 projection (12.11%), a high variance was verified between the C-STH and the remaining centroids. Finally, in PLS3 projection (7.20%), a substantial variance was observed between the C-MED and the other centroids. 

**Figure 4:**
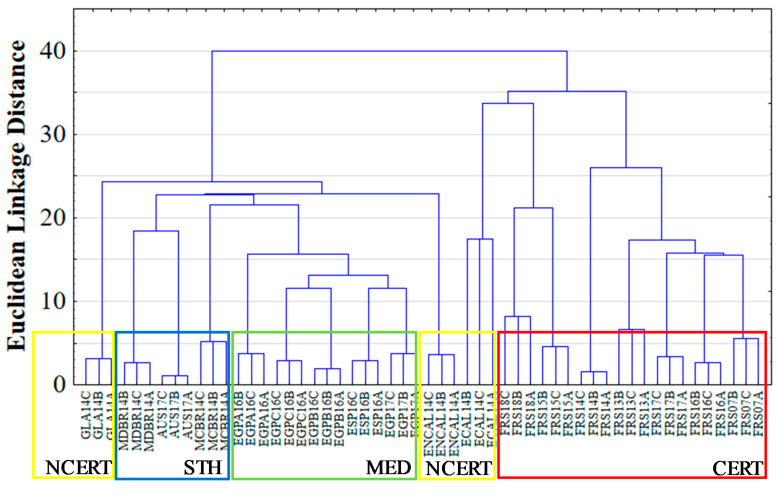
The HCA dendrogram for all replicates (A, B and C) from all samples (NCERT—non-certified producer; STH—Southern hemosphere region producers; MED—Mediterranean region producers; CERT—certified producer.

HCA was completed according to the selected 32 VOCs to define the Euclidean linkage distances between all 54 replicates. The HCA dendrogram is shown in Figure 4.

The higher Euclidean distance was verified between samples from the certified producer and the remaining syrups. The ECAL14 sample from non-certified regional producers presented a substantial proximity to the samples from the certified producer. On the contrary, the syrups from non-certified regional producers, GLA14 and ENCAL14, presented a higher distance from samples of the certified producer, and also among themselves. Another interesting fact regards the proximity between the various syrup samples from the Mediterranean region (Spain and Egypt). The PLS projection and HCA results confirmed that it is possible to differentiate the genuine SCH from other syrups based only on the 32 most predictive VOCs, proving their potential as useful markers for the traceability and authenticity of SCH on the global market.

## 4. Conclusions

The HS-SPME/GC-MS methodology was successfully applied for the establishment of the volatile profile of SCH samples. A total of 166 different VOCs were identified from which 119 were common in all investigated samples. FUR, KET and CAC were the most dominant chemical classes being responsible for a large fraction of the SCH volatile profile in number of VOCs and RPA values. HM5FURAL, DHYPPAONE and ETNOIC were the main identified VOCs. Interestingly, FUR was the main chemical class for all samples from the certified regional producer, and KET the most dominant class for the other syrups samples.

The ANOVA revealed that 144 VOCs showed statistically significant differences between all syrups. PCA and PLS, using full data matrix, demonstrated that the highest level of differentiation was verified between samples from certified producers and other syrup samples. The selection of the 32 most predictive VOCs based on the LDA proved their high predictive capacity, where a high level of differentiation was reached between samples from the regional certified producers and the non-certified producers, Mediterranean producers (Spain and Egypt) and south hemisphere producers (Brazil and Australia). Once again, the highest differentiation level was verified between samples from the certified producer and other syrup samples.

According to the results from the chemometric analysis, we concluded that the establishment of a volatile profile appears to be a promising strategy to identify genuine SCH from other syrups on the market, and also to discriminate the syrups based on their geographical origin. Furthermore, the specificity of some VOCs for a group of syrup samples could be a potential marker. This information is fundamental for guaranteeing the traceability and authenticity of SCH on the global market and, consequently, to support its submission process for EU certification.

## Figures and Tables

**Table 1:** LDA results after matrix reduction method to 20% of original dimension based on the VOCs with higher *F* values from One-way ANOVA.

Volatile Organic Compounds	Abbreviations	ANOVA	LDA					PLS				
		F ^1^	W ^2^	F ^3^	CDF ^4^			Loading Value			VIP ^5^	
					1	2	3	1	2	3	Importance	Power (× 100)
2,4,6-Trihydroxypyrimidine	THDXYPYMNE	180.09	4.62 × 10^−2^	130.62	−70.540	−8.258	14.822	−0.107	−0.339	−0.167	2	22.49
1,4-Pentadiene	PT14DIENE	49.98	2.70 × 10^−1^	17.12	26.030	−39.002	1.486	−0.063	−0.420	0.200	17	15.85
Pentane	PTANE	48.69	1.96 × 10^−1^	25.97	−34.120	12.658	−10.012	0.187	0.035	0.091	16	15.91
4-Pyridinol	PYRDINOL	45.77	1.73 × 10^−1^	30.19	−162.900	51.083	−51.729	0.181	0.065	−0.015	5	21.08
4-Cyclopentene-1,3-dione	CPT4E13DONE	42.70	5.21 × 10^−1^	5.82	−4.580	−17.467	10.460	0.188	−0.016	0.007	20	15.15
Ethanol	ETOL	38.57	2.63 × 10^−1^	17.70	−15.990	−9.449	−3.794	0.167	0.007	0.188	13	16.76
2,5-Furandicarboxaldehyde	FUR25DIAL	30.06	3.12 × 10^−1^	13.95	71.890	−55.246	−11.369	0.179	−0.069	0.049	10	17.99
5-Methyl-2(3H)-Furanone	M5FURONE	29.65	1.88 × 10^−1^	27.32	−9.110	−61.784	18.575	0.197	−0.085	0.005	25	12.42
2-(2-Furanylmethyl)-5-Methyl-Furan	FURYLMFUR	28.08	1.36 × 10^−1^	40.21	88.730	−8.719	24.338	−0.008	0.187	0.392	4	22.12
2-Methyl-Benzofuran	M2BNZFUR	27.39	6.90 × 10^−2^	85.49	−185.440	48.514	−37.864	0.179	−0.041	−0.087	8	18.11
2,2’-Methylenebis 5-Methyl-Furan	MNEB5MFUR	24.37	1.02 × 10^−1^	55.62	105.390	46.353	2.323	0.189	−0.029	0.012	9	18.10
Oxypurinol	OXYPUROL	23.80	2.85 × 10^−1^	15.90	29.440	−11.140	−8.239	0.188	−0.126	0.032	18	15.48
2-Cyclohexenol	CHEX2E1OL	23.74	1.88 × 10^−1^	27.37	122.070	−26.365	46.694	0.197	0.023	0.067	19	15.46
2-Methyl-Dihydro-2(3H)-Furanone	MDH2FURONE	23.37	5.09 × 10^−1^	118.02	87.910	−10.980	6.705	−0.041	−0.436	0.141	12	17.01
5-Acetoxymethyl-2-Furfural B	ACTYMFURALB	22.74	3.75 × 10^−2^	162.39	−220.540	45.621	−33.535	−0.043	−0.443	0.068	11	17.51
Furfural Acetone	FURALTONE	22.71	1.14 × 10^−1^	49.18	−279.810	36.381	−44.076	0.198	−0.013	−0.001	21	14.86
3-Methyl-2,4(3H,5H)-Furandione	M3FURDIONE	22.67	6.43 × 10^−2^	92.12	287.950	−18.438	49.078	0.190	−0.022	0.027	6	19.74
3-Methoxy-1,2-Benzenediol	M3BNZDIOL	22.36	6.43 × 10^−2^	92.12	−206.700	126.409	−33.892	0.203	−0.072	−0.031	32	9.20
Furfuryl Acetate	FURYLACTE	20.88	Removed from analysis.									
2-Furanpropionic Acid	FURPPIONIC	19.07	6.24 × 10^−2^	95.17	303.870	−13.701	39.789	−0.079	−0.128	−0.415	1	23.54
3-Methyl-Pyridazine	M3PYRDZNE	17.95	1.32 × 10^−1^	41.58	131.650	−2.761	−0.685	0.197	0.016	0.011	24	12.79
2-Methyl-Butanal	M2BTAL	17.62	3.43 × 10^−1^	12.13	0.060	−23.095	7.286	0.199	−0.047	−0.097	14	16.51
Cyclotene	CYTENE	17.59	3.19 × 10^−1^	13.49	−83.600	−28.243	12.252	0.201	−0.091	0.002	31	10.46
2-Acetylpyrrole	ACTLPYROLE	17.38	9.14 × 10^−2^	62.99	−90.260	55.198	−30.992	0.188	−0.146	−0.003	27	11.84
Furfuryl Formate	FURYLFMTE	17.23	6.55 × 10^−1^	3.33	−17.910	7.134	−37.968	0.185	−0.001	−0.129	15	16.08
2,3-Dihydro-1,1,4,6-Tetramethyl-1H-Indene	DHT1146MIDNE	17.05	4.92 × 10^−1^	6.53	36.680	0.135	3.888	0.179	0.075	0.090	23	13.62
3-Methyl-Furfural	M3FURAL	16.94	2.36 × 10^−1^	20.52	23.630	−29.167	15.070	0.201	0.001	−0.083	30	11.26
2-Ethyl-Hexanoic Acid	E2HXNOIC	16.91	1.10 × 10^−1^	51.37	36.630	−16.971	11.398	0.196	−0.022	−0.128	22	14.73
Maltol	MALTOL	15.39	2.29 × 10^−1^	21.26	53.870	3.310	19.627	−0.123	−0.085	−0.066	3	22.20
Tetrahydro-5-Methyl-2-Furanmethanol	TEHYMFUROL	15.38	6.09 × 10^−2^	97.61	−46.990	11.888	1.765	0.177	−0.003	−0.206	7	18.33
3,5-Xylenol	XYL35NOL	15.25	7.90 × 10^−2^	73.85	−137.460	−96.967	−1.898	0.201	−0.088	−0.018	29	11.29
2,3-Dihydro-1,1,5,6-Tetramethyl-1H-Indene	DHT1156MIDNE	14.77	6.28 × 10^−1^	3.76	−4.680	56.537	−28.156	0.204	−0.007	−0.004	28	11.42
Decanal	DECAL	14.47	3.55 × 10^−2^	172.01	125.780	−31.467	30.132	0.195	−0.030	−0.020	26	11.95

^1^ F—F value from One-way ANOVA test. ^2^ W—W value from Linear Discriminant Analysis. ^3^ F—F value from Linear Discriminant Analysis. ^4^ CDF—Canonical Discriminant Function Coefficients. ^5^ VIP—Variable importance for projection

## Data Availability

Not applicable.

## Supplementary Materials

The following are available online at https://www.mdpi.com/article/10.3390/foods10071559/s1, Figure S1: The GC-MS chromatograms for FRS07 (A), FRS13 (B), FRS14 (C), FRS15 (D), FRS16 (E), FRS17 (F), FRS18 (G), ECAL14 (H), NCAL14 (I), GLA14 (J), ESP16 (K), EGP17 (L), EGPA16 (M), EGPB16 (N), EGPC16 (O), MDBR14 (P), MCBR14 (Q) and AUS17 (R) samples. Figure S2. The PCA loadings line plot of all samples for PC1 (A), PC2 (B) and PC3 (C), and the PLS loadings line plot of all samples for PLS1 (D), PLS2 (E) and PLS3 (F). Figure S3. The LDA loading line plots of all samples for to CDF1 (A), CDF2 (B) and CDF3 (C), and LDA loading line plots for CDF1 (D), CDF2 (E) and CDF3 (F). Figure S4. The PLS loadings line plots of all samples based on the 32 most predictive VOCs for PLS1 (A), PLS2 (B) and PLS3 (C), and the PLS loadings line plots of the 32 most predictive VOCs for PLS1 (D), PLS2 (E) and PLS3 (F). Figure S5: The PLS 3D plot (A) and HCA dendrogram (B) for results from the matrix reduction procedure based on the VIP scores. Table S1. Information of sugarcane-based syrups samples. Table S2. Information of identified VOCs in sugarcane-based syrups samples. Table S3A. Mean and relative standard deviation values of VOCs from CERT group. Table S3B: Mean and relative standard deviation values of VOCs from NCERT group. Table S3C: Mean and relative standard deviation values of VOCs from MED group. Table S3D: Mean and relative standard deviation values of VOCs from STH group. Table S4: Mean, minimum and maximum peak area values of VOCs. Table S5: VOCs identified in samples from this study and previously identified in others sugarcane-based syrups from other studies. Table S6A: Number of VOCs identified, relative peak areas and total relative peak areas values of main chemical classes from CERT group. Table S6B: Number of volatile organic compounds identified, relative peak areas and total relative peak areas values of main chemical classes from NCERT group. Table S6C: Number of VOCs identified, relative peak areas and total relative peak areas (%) values of main chemical classes from MED group. Table S6D: Number of VOCs identified, relative peak areas and total relative peak areas values of main chemical classes from STH group. Table S7: One-way ANOVA test results based on the relative peak areas of the 147 VOCs. Table S8: Information of PCA and PLS. Table S9: Loading results and variable importance in projection scores of variables from PCA and PLS. Table S10: Loading results of samples and variables from PCA and PLS. Table S11: Scores results of all cases from PCA and PLS. Table S12: Canonical Discriminant Function Coefficients and Highest Probability Classification results. Table S13. Information of PLS based only on the relative peak areas of the 32 most predictive VOCs. Table S14: Results of LDA and PLS analysis based on the relative peak areas of the most predictive VOCs.
