# Supplementary material for: A Predictive Strategy Based on Volatile Profile and Chemometric Analysis for Traceability and Authenticity of Sugarcane Honey on the Global Market
Source: Foods. 2021 Jul 5;10(7):1559. doi: 10.3390/foods10071559 (PMC8305931; doi:10.3390/foods10071559)
Supplement: Supplementary file 1 [file foods-10-01559-s001.zip › foods-1241370-supplementary.pdf]

A predictive strategy based on volatile profile and chemometric analysis for traceability and authenticity of sugarcane honey on the global market

SUPPLEMEMNTARY INFORMATION

Supplementary Information

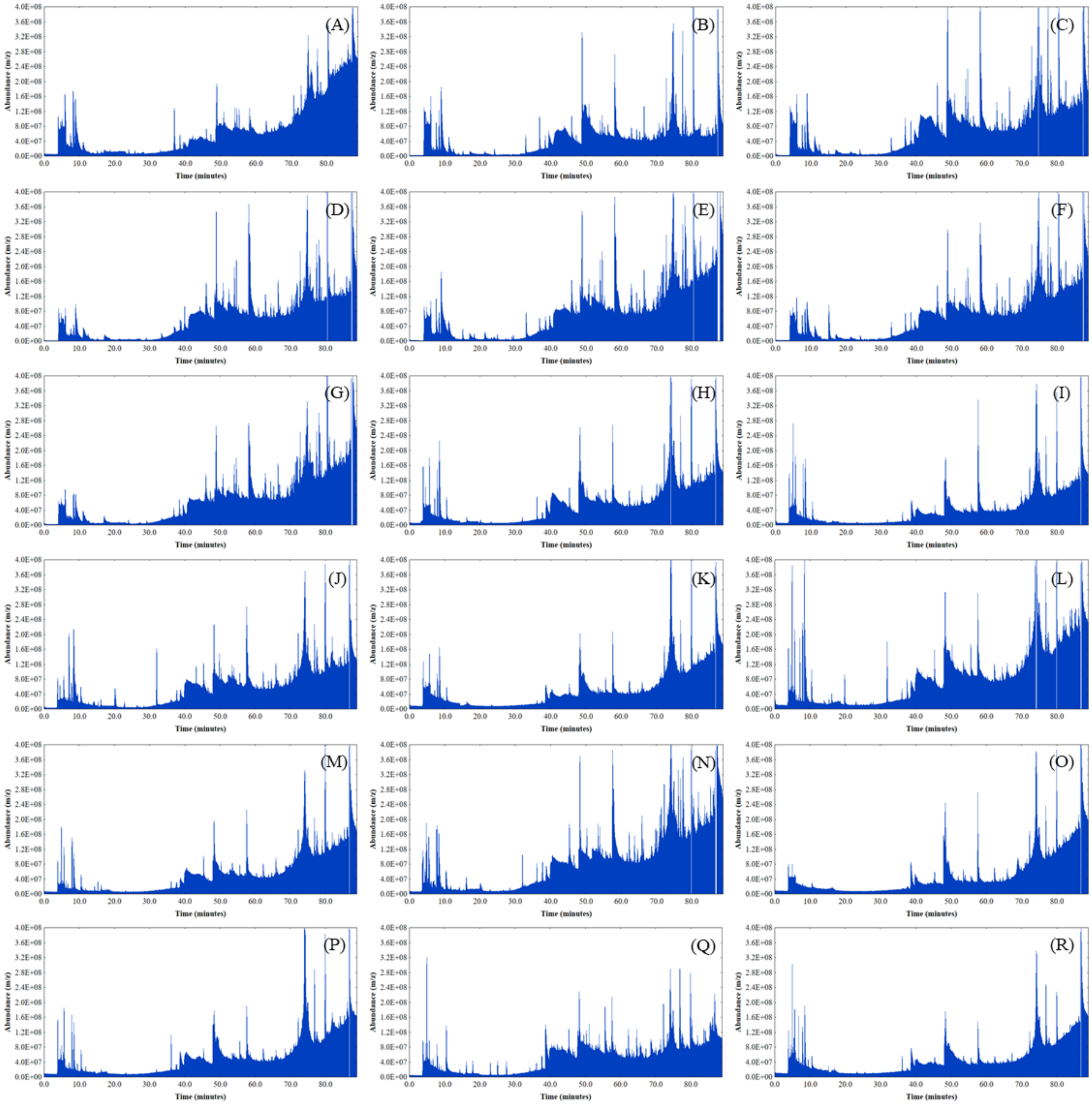

Figure S1. The typical GC-MS chromatograms for FRS07 (A), FRS13 (B), FRS14 (C), FRS15 (D), FRS16 (E), FRS17 (F), FRS18 (G), ECAL14 (H), NCAL14 (I), GLA14 (J), ESP16 (K), EGP17 (L), EGPA16 (M), EGPB16 (N), EGPC16 (O), MDBR14 (P), MCBR14 (Q) and AUS17 (R) samples.

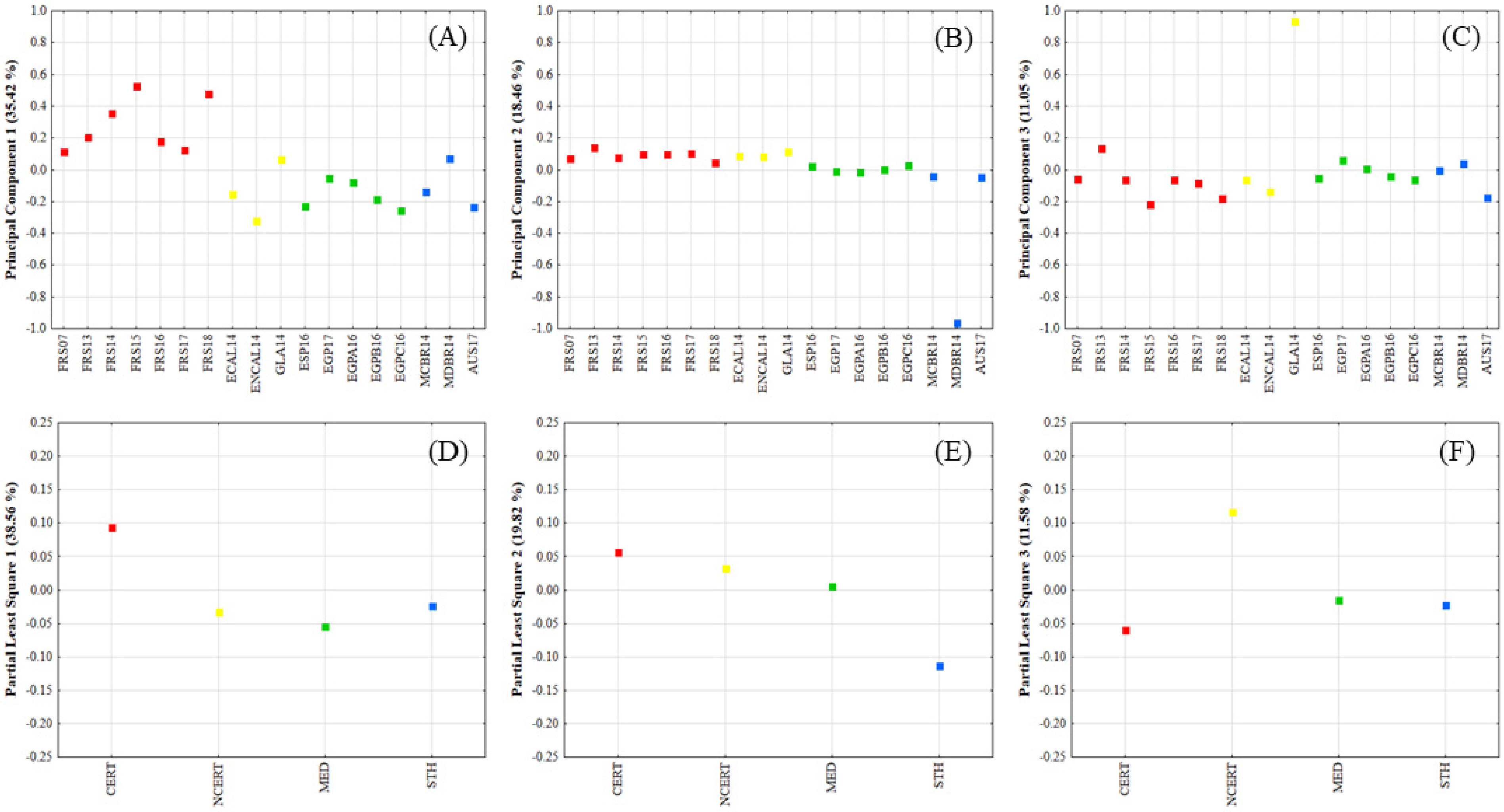

Figure S2. The PCA loadings line plot of all stage samples based on the selected 144 VOCs for PC1 (A), PC2 (B) and PC3 (C), and the PLS loadings line plot of all cultivar samples for PLS1 (D), PLS2 (E) and PLS3 (F).

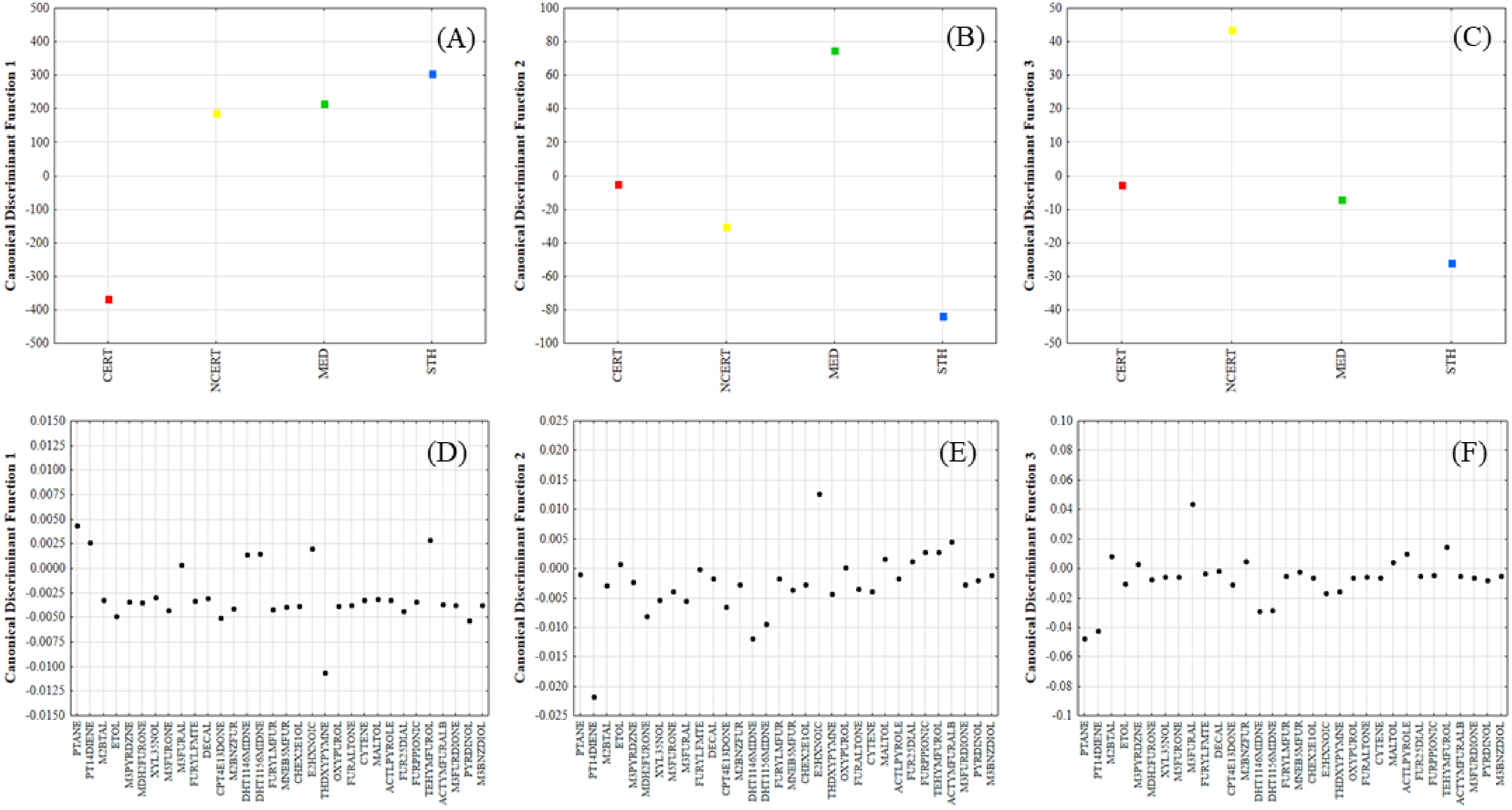

Figure S3. The LDA line plots of all cultivars based on the 31 most predictive VOCs for to CDF1 (A), CDF2 (B) and CDF3 (C), and LDA line plots of the 35 most predictive volatile organic compounds for CDF1 (D), CDF2 (E) and CDF3 (F).

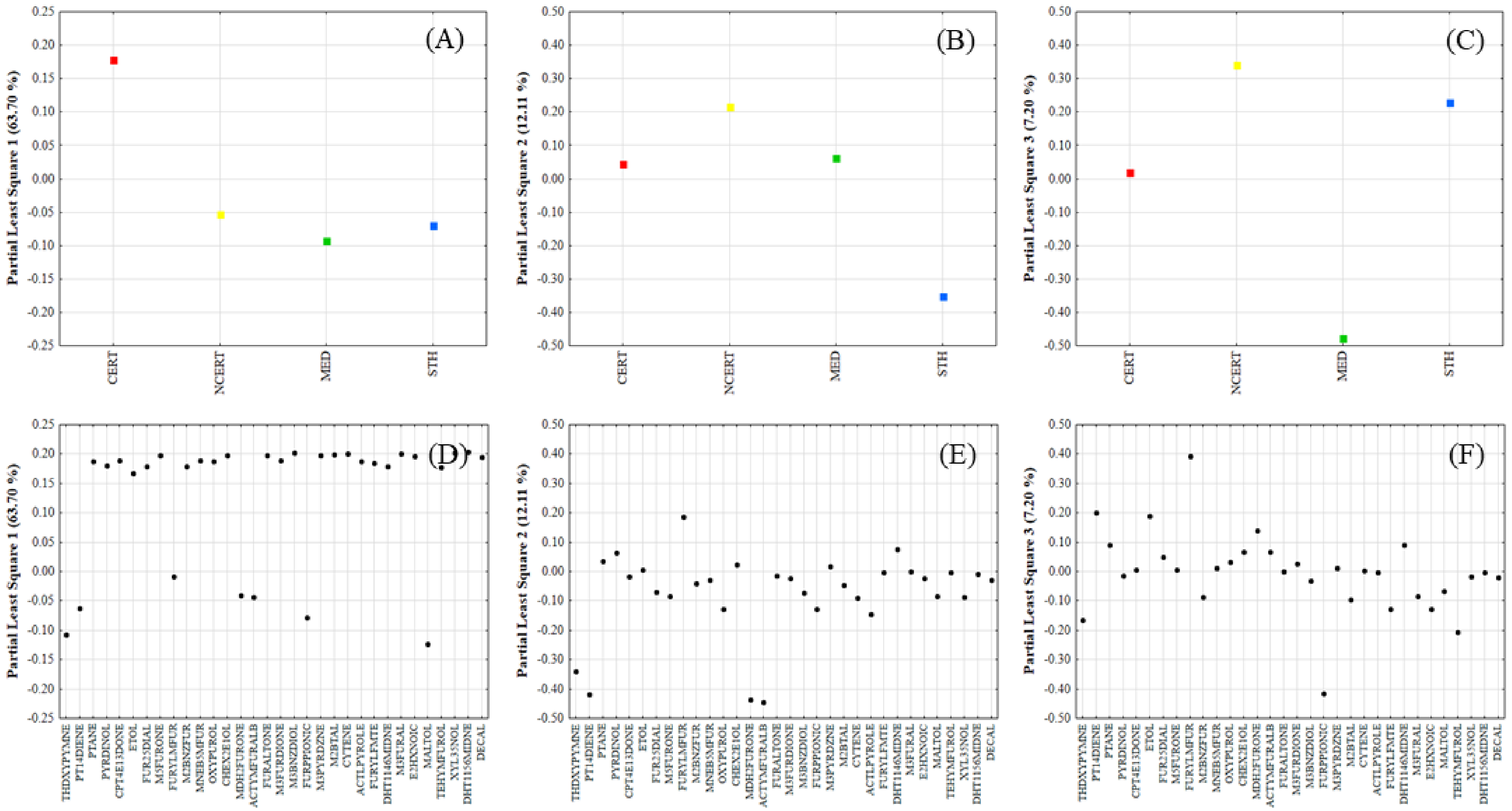

Figure S4. The PLS loadings line plots of all cultivar samples based on the 31 most predictive VOCs for PLS1 (A), PLS2 (B) and PLS3 (C), and the PLS loadings line plots of the 35 most predictive VOCs for PLS1 (D), PLS2 (E) and PLS3 (F).

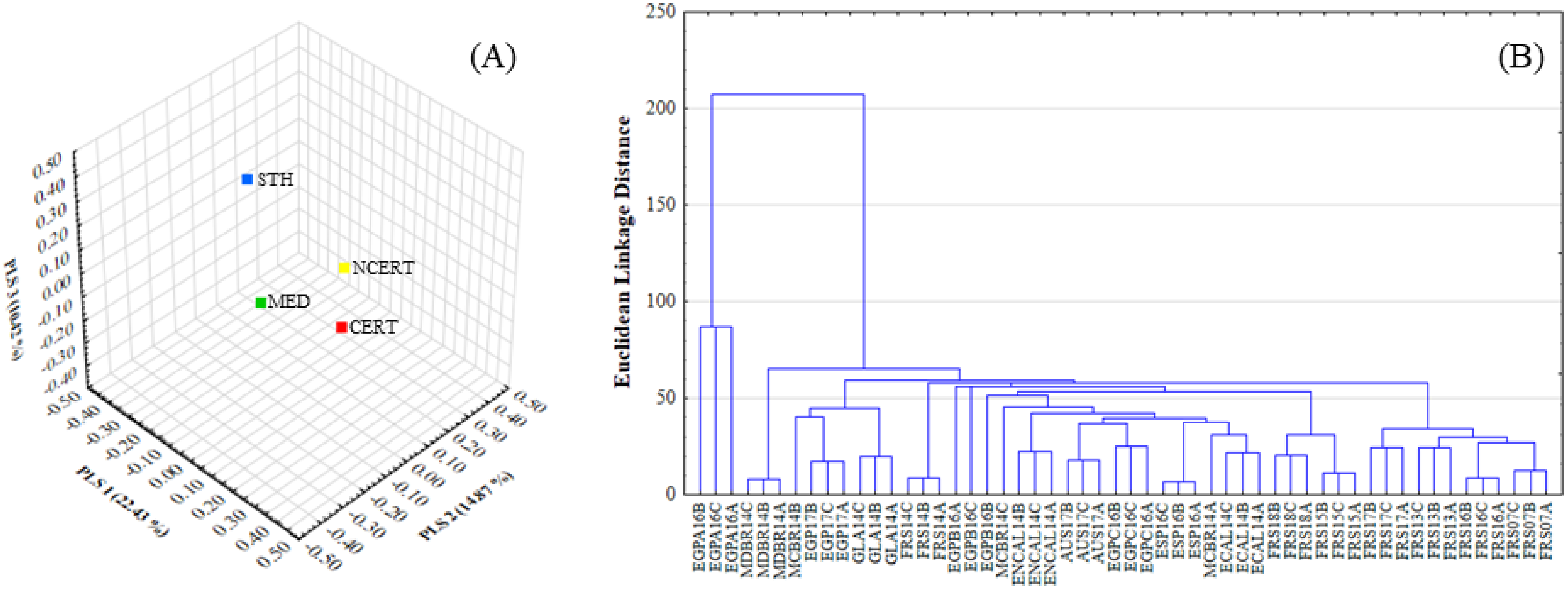

Figure S5. The PLS 3D plot (A) and HCA dendrogram (B) for results from the matrix reduction procedure based on the VIP scores.

Table S1. ID replicate number, ID replicate code, ID sample code, ID group code, processing year, processing type, geographic origin and regional certification of sugarcane-based syrups samples.

ID Replicate Number	ID Replicate Code	ID Sample Code	ID Group Code	Processing Year	Processing Type	Geographic Origin	Regional Certification
1	FRS07A						
2	FRS07B	FRS07	CERT	2007	Industrial	Madeira Island, Portugal, Atlantic Region	Certified
3	FRS07C						
4	FRS13A						
5	FRS13B	FRS13	CERT	2013	Industrial	Madeira Island, Portugal, Atlantic Region	Certified
6	FRS13C						
7	FRS14A						
8	FRS14B	FRS14	CERT	2014	Industrial	Madeira Island, Portugal, Atlantic Region	Certified
9	FRS14C						
10	FRS15A						
11	FRS15B	FRS15	CERT	2015	Industrial	Madeira Island, Portugal, Atlantic Region	Certified
12	FRS15C						
13	FRS16A						
14	FRS16B	FRS16	CERT	2016	Industrial	Madeira Island, Portugal, Atlantic Region	Certified
15	FRS16C						
16	FRS17A						
17	FRS17B	FRS17	CERT	2017	Industrial	Madeira Island, Portugal, Atlantic Region	Certified
18	FRS17C						
19	FRS18A						
20	FRS18B	FRS18	CERT	2018	Industrial	Madeira Island, Portugal, Atlantic Region	Certified
21	FRS18C						
22	ECAL14A						
23	ECAL14B	ECAL14	NCERT	2014	Industrial	Madeira Island, Portugal, Atlantic Region	Non-Certified
24	ECAL14C						
25	ENCAL14A						
26	ENCAL14B	ENCAL14	NCERT	2014	Industrial	Madeira Island, Portugal, Atlantic Region	Non-Certified
27	ENCAL14C						
28	GLA14A	ECAL16	NCERT	2014	Home-made		Non-Certified

29	GLA14B					Madeira Island, Portugal, Atlantic Region	
30	GLA14C						
31	ESP16A						
32	ESP16B	ESP16	MED	2016	Industrial	Spain, Mediterranean Region	Not Applicable
33	ESP16C						
34	EGP17A						
35	EGP17B	EGP17	MED	2017	Industrial	Spain, Mediterranean Region	Not Applicable
36	EGP17C						
37	EGPA16A						
38	EGPA16B	EGPA16	MED	2016	Industrial	Spain, Mediterranean Region	Not Applicable
39	EGPA16C						
40	EGPB16A						
41	EGPB16B	EGPB16	MED	2016	Industrial	Spain, Mediterranean Region	Not Applicable
42	EGPB16C						
43	EGPC16A						
44	EGPC16B	EGPC16	MED	2016	Industrial	Egypt, Mediterranean Region	Not Applicable
45	EGPC16C						
46	MCBR14A						
47	MCBR14B	MCBR14	STH	2014	Industrial	Brazil, Southern Hemisphere Region	Not Applicable
48	MCBR14C						
49	MDBR14A						
50	MDBR14B	MDBR14	STH	2014	Industrial	Brazil, Southern Hemisphere Region	Not Applicable
51	MDBR14C						
52	AUS17A						
53	AUS17B	AUS17	STH	2017	Industrial	Australia, Southern Hemisphere Region	Not Applicable
54	AUS17C						

Table S2. ID number, retention time, main ions (m/z), target ion, match percent, IUPAC name, NIST database, abbreviation, CAS number, molecular formula and main chemical class of identified VOCs in sugarcane-based syrups samples.

ID Number	RT[a]	m/z	TI[b]	MP[c] (%)	ID Type[d]	IUPAC Name	NIST Database Name	Abbreviations	CAS Number	Molecular Formula	Main Chemical Class
1	4,253	43; 42; 41	43	86	RS; MS	pentane	Pentane	PTANE	109-66-0	C5H12	Hydrocarbon
2	4,518	57; 41; 56	57	84	RS; MS	hexane	Hexane	HXANE	110-54-3	C6H14	Hydrocarbon
3	4,586	67; 68; 53	67	90	MS	penta-1,4-diene	1,4-Pentadiene	PT14DIENE	591-93-5	C5H8	Hydrocarbon
4	4,709	47; 48; 45	47	87	RS; MS	methanethiol	Methanethiol	METHIOL	74-93-1	CH4S	Sulfur
5	4,903	44; 43; 42	44	88	RS; MS	acetaldehyde	Ethanal	ETAL	75-07-0	C2H4O	Aldehyde
6	5,282	47; 62; 45	47	97	RS; MS	methylsulfanylmethane	Dimethyl Sulfide	DMSULFI	75-18-3	C2H6S	Sulfur
7	5,713	58; 57; 59	58	79	RS; MS	propanal	Propanal	PPAL	123-38-6	C3H6O	Aldehyde
8	5,830	68; 39; 40	68	91	RS; MS	furan	Furan	FUR	110-00-9	C4H4O	Furan
9	6,041	43; 41; 72	72	86	MS	2-methylpropanal	2-Methyl-Propanal	MPPAL	78-84-2	C4H8O	Aldehyde
10	6,255	31; 45; 74	31	80	RS; MS	ethyl formate	Ethyl Ester Formic Acid	EESTFA	109-94-4	C3H6O2	Ester
11	6,632	56; 55; 37	56	78	MS	prop-2-enal	2-Propenal	PPENAL	107-02-8	C3H4O	Aldehyde
12	7,199	82; 53; 81	82	94	MS	2-methylfuran	2-Methyl-Furan	M2FUR	534-22-5	C5H6O	Furan
13	7,591	43; 61; 70	43	90	RS; MS	ethyl acetate	Ethyl Ester Acetic Acid	EESTAA	141-78-6	C4H8O2	Ester
14	7,906	43; 72; 57	72	78	RS; MS	butan-2-one	2-Butanone	BT2ONE	78-93-3	C4H8O	Ketone
15	8,338	57; 41; 58	57	86	MS	2-methylbutanal	2-Methyl-Butanal	M2BTAL	96-17-3	C5H10O	Aldehyde
16	8,494	44; 41, 43	41	93	MS	3-methylbutanal	3-Methyl-Butanal	M3BTAL	590-86-3	C5H10O	Aldehyde
17	8,946	31; 45; 46	31	78	RS; MS	ethanol	Ethanol	ETOL	64-17-5	C2H6O	Alcohol
18	9,379	78; 77; 52	78	83	RS; MS	Benzene	Benzene	BNZ	71-43-2	C6H6	Benzene
19	9,569	55; 43; 70	55	80	MS	but-3-en-2-one	3-Buten-2-one	BT3ONE	78-94-4	C4H6O	Ketone
20	10,058	96; 95; 43	96	94	MS	2,5-dimethylfuran	2,5-Dimethyl-Furan	DM25FUR	625-86-5	C6H8O	Furan
21	10,190	57; 75; 102	57	82	MS	ethyl propanoate	Ethyl Ester Propanoic Acid	EESTPA	105-37-3	C5H10O2	Ester
22	11,412	43; 86; 41	86	80	MS	butane-2,3-dione	2,3-Butanedione	BT23DONE	431-03-8	C4H6O2	Ketone
23	12,557	43; 56; 73	56	82	MS	2-methylpropyl acetate	2-Methylpropyl Ester Acetic Acid	MP2ESTAA	110-19-0	C6H12O2	Ester
24	14,801	31; 59; 42	31	80	RS; MS	propan-1-ol	1-Propanol	P1POL	71-23-8	C3H8O	Alcohol
25	14,903	95; 110; 43	95	86	MS	2-ethyl-5-methylfuran	2-Ethyl-5-Methyl-Furan	E2M5FUR	1703-52-2	C7H10O	Furan
26	15,111	91; 92; 65	91	94	RS; MS	toluene	Toluene	TOLNE	108-88-3	C7H8	Benzene

27	15,268	43; 71; 88	88	84	MS	ethyl butanoate	Ethyl Ester Butanoic Acid	EESTBA	105-54-4	C6H12O2	Ester
28	15,327	71; 43; 59	71	78	MS	2-methylbut-3-en-2-ol	2-Methyl-3-Buten-2-ol	M3BT2OL	115-18-4	C5H10O	Alcohol
29	16,166	101; 55; 73	101	82	MS	nonomethyl succinate	Monomethyl Ester Butanedioic Acid	METESTBA	3878-55-5	C5H8O4	Ester
30	17,382	43; 57; 100	57	83	MS	pentane-2,3-dione	2,3-Pentanedione	PTDONE	600-14-6	C5H8O2	Ketone
31	18,081	94; 45; 79	94	90	MS	(methyldisulfanyl)methane	Dimethyl Disulfide	DMDSFD	624-92-0	C2H6S2	Sulfur
32	18,284	94; 65; 39	94	78	MS	2-ethenylfuran	2-Ethenyl-Furan	ENYL2FUR	1487-18-9	C6H6O	Furan
33	18,723	81; 99; 55	81	83	MS	2,5-diethyloxolane	2,5-Diethyltetrahydro-Furan	DE25TTHYFUR	41239-48-9	C8H16O	Furan
34	18,905	44; 56; 43	44	94	RS; MS	hexanal	Hexanal	HXAL	66-25-1	C6H12O	Aldehyde
35	21,231	43; 42; 41	43	90	RS; MS	2-methylpropan-1-ol	2-Methyl-1-Propanol	M2PP1OL	78-83-1	C4H10O	Alcohol
36	25,551	85; 41; 56	85	80	MS	5-butyloxolan-2-one	5-Butyl-Dihydro-2(3H)-Furanone	BDH2FURONE	104-50-7	C8H14O2	Furan
37	27,262	108; 107; 77	108	80	MS	3-methylphenol	m-Cresol	MCREOL	108-39-4	C7H8O	Phenol
38	27,966	56; 31; 41	56	80	RS; MS	butan-1-ol	1-Butanol	BT1OL	71-36-3	C4H10O	Alcohol
39	28,085	85; 56; 41	85	82	MS	5-propyloxolan-2-one	5-Propyl-Dihydro-2(3H)-Furanone	PDH2FURONE	105-21-5	C7H12O2	Furan
40	29,054	94; 39; 65	94	87	MS	3-methylpyridazine	3-Methyl-Pyridazine	M3PYRDZNE	1632-76-4	C5H6N2	Nitrogen
41	29,851	43; 58; 71	58	91	RS; MS	heptan-2-one	2-Heptanone	HPT2ONE	110-43-0	C7H14O	Ketone
42	32,080	95; 138; 67	95	85	MS	1-methyl-3-propan-2-ylcyclohexane	m-Menthane	MMTHANE	16580-24-8	C10H20	Terpenoid
43	32,436	80; 53; 52	80	80	MS	pyrimidine	1,3-Diazine	D13ZINE	289-95-2	C4H4N2	Nitrogen
44	32,630	57; 41; 56	57	83	RS; MS	2-methylbutan-1-ol	2-Methyl-1-Butanol	M2BT1OL	137-32-6	C5H12O	Alcohol
45	33,174	82; 53; 81	82	90	MS	2-propylfuran	2-Propyl-Furan	PP2FUR	4229-91-8	C7H10O	Furan
46	34,163	88; 43; 99	88	83	MS	ethyl hexanoate	Ethyl Ester Hexanoic Acid	EESTHA	123-66-0	C8H16O2	Ester
47	34,394	81; 82; 138	81	94	MS	2-pentylfuran	2-Pentyl-Furan	PT2FUR	3777-69-3	C9H14O	Furan
48	36,565	94; 67; 53	94	81	MS	2-methylpyrazine	2-Methyl-Pyrazine	MPYZNE	109-08-0	C5H6N	Nitrogen
49	37,622	43; 72; 100	72	91	MS	2-methyloxolan-3-one	2-Methyl-Dihydro-2(3H)-Furanone	MDH2FURONE	3188-00-9	C5H8O2	Furan
50	37,261	119; 134; 91	119	84	MS	1-methyl-4-propan-2-ylbenzene	p-Cymene	PCYMNE	99-87-6	C10H14	Terpenoid
51	37,369	105; 120; 77	105	95	MS	1,3,5-trimethylbenzene	Mesitylene	MESTLNE	108-67-8	C9H12	Benzene
52	38,007	122; 107; 121	122	80	MS	3,5-dimethylphenol	3,5-Xylenol	XYL35NOL	108-68-9	C8H10O	Phenol
53	38,238	45; 43; 88	45	90	MS	3-hydroxybutan-2-one	3-Hydroxy-2-Butanone	HXY3BT2ONE	513-86-0	C4H8O2	Ketone (Alcohol)
54	38,261	86; 42; 58	86	82	MS	1,3-dioxol-2-one	Vinylene Carbonate	VYLESTCA	96-49-1	C3H4O3	Ester (Ketone)

55	38,738	122; 107; 121	122	82	MS	2,6-dimethylphenol	2,6-Xylenol	XYL26NOL	576-26-1	C8H10O	Phenol
56	39,324	43; 31; 74	43	90	MS	1-hydroxypropan-2-one	1-Hydroxy-2-Propanone	HXY1PP2ONE	116-09-6	C3H6O2	Ketone (Alcohol)
57	40,500	108; 42; 40	108	80	MS	2,5-dimethylpyrazine	2,5-Dimethyl-Pyrazine	DM25PYZNE	123-32-0	C6H8N2	Nitrogen
58	40,910	108; 42; 40	108	90	MS	2,6-dimethylpyrazine	2,6-Dimethyl-Pyrazine	DM26PYZNE	108-50-9	C6H8N2	Nitrogen
59	42,422	82; 39; 54	82	91	MS	cyclopent-2-en-1-one	2-Cyclopenten-1-one	CY2PT1EONE	930-30-3	C5H6O	Ketone
60	41,788	159; 174; 144	159	82	MS	1,2,3,4-tetramethyl-5-prop-1-en-2-ylbenzene	1,2,3,4-Tetramethyl-4-(1-methylethenyl)-Benzene	TM4MEYL1BNZ	61142-76-5	C13H20	Benzene
61	43,239	67; 96; 53	67	83	MS	2-methylcyclopent-2-en-1-one	2-Methyl-2-Cyclopenten-1-one	M2CY2PT1EONE	1120-73-6	C6H8O	Ketone
62	43,444	95; 43; 96	95	80	MS	(5-methylfuran-2-yl)methanethiol	5-Methyl-2-Furanmethanethiol	M2FURTHOL	59303-05-8	C6H8OS	Furan (Sulfur)
63	43,682	57; 88; 56	57	79	MS	1-hydroxybutan-2-one	1-Hydroxy-2-Butanone	H1BT2ONE	5077-67-8	C4H8O2	Ketone (Alcohol)
64	44,389	121; 122; 39	121	80	MS	2-ethyl-6-methylpyrazine	2-Ethyl-6-Methyl-Pyrazine	E6MPYZNE	13925-03-6	C7H10N2	Nitrogen
65	44,691	121; 122; 39	121	77	MS	2-ethyl-5-methylpyrazine	2-Ethyl-5-Methyl-Pyrazine	E5MPYZNE	13360-64-0	C7H10N2	Nitrogen
66	45,234	57; 41; 43	57	93	RS; MS	nonanal	Nonanal	NONAL	124-19-6	C9H18O	Aldehyde
67	45,745	42; 122; 81	122	78	MS	2,3,5-trimethylpyrazine	Trimethyl-Pyrazine	TMPYZNE	14667-55-1	C7H10N2	Nitrogen
68	45,956	67; 95; 124	67	81	MS	(3E)-3-ethyl-2-methylhexa-1,3-diene	3-Ethyl-2-Methyl-1,3-Hexadiene	E3M2HX13DENE	61142-36-7	C9H16	Terpene
69	46,726	68; 96; 40	68	82	MS	cyclohex-2-en-1-one	2-Cyclohexen-1-one	C2HEXONE	930-68-7	C6H8O	Ketone
70	46,901	55; 43; 98	55	87	MS	2-methyl-2H-furan-5-one	5-Methyl-2(3H)-Furanone	M5FURONE	591-12-8	C5H6O2	Furan
71	47,214	98; 42; 69	98	84	MS	4-methyl-1,3-dihydroimidazol-2-one	1,3-Dihydro-4-Methyl-2H-Imidazol-2-one	M4IMDZONE	214-751-9	C4H6N2O	Nitrogen (Ketone)
72	47,569	88; 101; 57	88	89	MS	ethyl octanoate	Ethyl Ester Octanoic Acid	EESTOA	106-32-1	C10H20O2	Ester
73	47,861	159; 174; 131	159	94	MS	1,2,3,4-tetrahydro-1,1,6-trimethylnaphthalene	1,2,3,4-Tetrahydro-1,1,6-Trimethyl-Naphthalene	T116TNP	475-03-6	C13H18	Naphthalene
74	48,559	96; 95; 39	96	96	RS; MS	2-furanaldehyde	Furfural	FURAL	98-01-1	C5H4O2	Furan (Aldehyde)
75	49,502	43; 45; 60	43	90	RS; MS	Acetic Acid	Ethanoic Acid	ETNOIC	64-19-7	C2H4O2	Carboxylic Acid
76	49,783	159; 174; 131	159	96	MS	1,2,3,4-tetrahydro-1,6,8-trimethylnaphthalene	1,2,3,4-Tetrahydro-1,6,8-Trimethyl-Naphthalene	T168TNP	30316-36-0	C13H18	Naphthalene
77	50,004	57; 41; 55	57	82	MS	2-ethylhexan-1-ol	2-Ethyl-1-Hexanol	E2HX1OL	104-76-7	C8H18O	Alcohol
78	50,131	110; 109; 53	110	92	MS	3-methyl-2-furanaldehyde	3-Methyl-Furfural	M3FURAL	33342-48-2	C6H6O2	Furan (Aldehyde)

79	50,259	81; 53; 126	81	82	MS	furan-2-ylmethyl formate	Furfuryl Formate	FURYLFMTE	13493-97-5	C6H6O3	Furan (Ester)
80	50,655	57; 43; 55	57	93	RS; MS	decanal	Decanal	DECAL	112-31-2	C10H2O	Aldehyde
81	50,895	95; 110; 39	95	91	RS; MS	1-(furan-2-yl)ethanone	1-(2-Furanyl)-Ethanone	FURYLONE	1192-62-7	C6H6O2	Furan (Ketone)
82	51,329	106; 105; 77	106	95	RS; MS	benzaldehyde	Benzaldehyde	BENZAL	100-52-7	C7H6O	Benzene (Aldehyde)
83	51,771	81; 98; 52	81	95	MS	furan-2-ylmethyl acetate	Furfuryl Acetate	FURYLACTE	623-17-6	C7H8O3	Furan (Ester)
84	52,336	74; 45; 73	74	90	RS; MS	propanoic acid	Propanoic Acid	PPANOIC	79-09-4	C3H6O2	Carboxylic Acid
85	53,078	63; 78; 45	63	91	MS	methylsulfinylmethane	Dimethyl Sulfoxide	DMSULFO	67-68-5	C2H6OS	Sulfur
86	53,245	159; 174; 131	159	94	MS	1,2,3,4-tetrahydro-1,5,8-trimethylnaphthalene	1,2,3,4-Tetrahydro-1,5,8-Trimethyl-Naphthalene	T158TNP	21693-51-6	C13H18	Naphthalene
87	53,657	110; 109; 53	110	94	MS	5-methyl-2-furanaldehyde	5-Methyl-Furfural	M5FURAL	620-02-0	C6H6O2	Furan (Aldehyde)
88	54,207	96; 42; 68	96	91	MS	cyclopent-4-ene-1,3-dione	4-Cyclopentene-1,3-dione	CPT4E13DONE	930-60-9	C5H4O2	Ketone
89	54,861	131; 132; 103	131	97	MS	2-methyl-1-benzofuran	2-Methyl-Benzofuran	M2BNZFUR	4265-25-2	C9H8O	Benzofuran
90	55,678	109; 124; 81	109	80	MS	1-(5-methylfuran-2-yl)ethanone	1-(5-Methyl-2-Furanyl)-Ethanone	M5FURYLONE	1193-79-9	C7H8O2	Furan (Ketone)
91	55,835	45; 59; 72	45	90	MS	2-(2-ethoxyethoxy)ethanol	Diethylene Glycol Ethyl Ether	DENGOLEETHR	111-90-0	C6H14O3	Ether (Alcohol)
92	56,251	42; 41; 56	42	82	MS	3-methyloxolan-3-one	3-Methyl-Dihydro-2(3H)-Furanone	M3DHFURONE	1679-47-6	C5H8O2	Furan (Lactone)
93	56,693	159; 174; 128	159	86	MS	3,3,5,7-tetramethyl-1,2-dihydroindene	2,3-Dihydro-1,1,4,6-Tetramethyl-1H-Indene	DHT1146MIDNE	941-60-6	C13H18	Indene
94	57,065	91; 92; 120	91	90	MS	2-phenylacetaldehyde	Benzeneacetaldehyde	BENZACETAL	122-78-1	C8H8O	Benzene (Aldehyde)
95	57,117	69; 41; 112	91	87	MS	2,4-dimethyl-2H-furan-5-one	3,5-Dimethyl-Dihydro-2-Furanone	DM35DHFURONE	5584-69-0	C6H8O2	Furan (Lactone)
96	57,323	72; 55; 45	72	78	MS	prop-2-enoic acid	2-Propenoic Acid	PPE2NOIC	79-10-7	C3H4O2	Carboxylic Acid
97	57,337	88; 101; 73	88	89	MS	ethyl decanoate	Ethyl Ester Decanoic Acid	EESTDA	110-38-3	C12H24O2	Ester
98	57,354	105; 77; 51	105	87	MS	1-phenylethanone	Acetophenone	ACETPHONE	98-86-2	C8H8O	Benzene (Ketone)
99	57,744	98; 41; 81	98	98	RS; MS	furan-2-ylmethanol	2-Furanmethanol	FUR2OL	98-00-0	C5H6O2	Furan (Alcohol)
100	57,981	98; 41; 97	97	93	MS	furan-3-ylmethanol	3-Furanmethanol	FUR3OL	4412-91-3	C5H6O2	Furan (Alcohol)
101	58,675	159; 128; 144	159	86	MS	3,3,5,6-tetramethyl-1,2-dihydroindene	2,3-Dihydro-1,1,5,6-Tetramethyl-1H-Indene	DHT1156MIDNE	942-43-8	C13H18	Indene
102	58,897	162; 91; 43	162	91	MS	2-(2-furylmethyl)-5-methylfuran	2-(2-Furanylmethyl)-5-Methyl-Furan	FURYLMFUR	13678-51-8	C10H10O2	Furan

103	60,229	41; 69; 98	41	83	MS	4-methyl-2H-furan-5-one	3-Methyl-2(5H)-Furanone	M3FURONE	22122-36-7	C5H6O2	Furan (Lactone)
104	60,456	95; 112; 43	95	93	MS	(5-methylfuran-2-yl)methanol	5-Methyl-2-Furanmethanol	M5FUR2OL	3857-25-8	C6H8O2	Furan (Alcohol)
105	60,947	108; 91; 90	108	75	MS	benzyl acetate	Phenylmethyl Ester Acetic Acid	PMESTAA	140-11-4	C9H10O2	Benzene (Ester)
106	61,749	55; 84; 54	55	80	MS	2H-furan-5-one	2(5H)-Furanone	FURONE	497-23-4	C4H4O2	Furan (Lactone)
107	62,073	176; 43; 161	176	96	MS	2-methyl-5-[(5-methylfuran-2-yl)methyl]furan	2,2'-Methylenebis[5-Methyl-Furan	MNEB5MFUR	13679-43-1	C10H10O2	Furan
108	62,421	98; 55; 42	98	90	MS	cyclopentane-1,2-dione	1,2-Cyclopentanedione	CPT12DONE	3008-40-0	C5H6O2	Ketone
109	63,971	70; 39; 41	70	84	MS	cyclohex-2-en-1-ol	2-Cyclohexenol	CHEX2E1OL	822-67-3	C6H10O	Alcohol
110	64,539	104; 43; 91	104	84	MS	2-phenylethyl acetate	2-Phenylethyl Ester Acetic Acid	PEESTAA	103-45-7	C10H12O2	Benzene (Ester)
111	64,803	112; 69; 41	112	93	MS	3-methylcyclopentane-1,2-dione	3-Methyl-1,2-Cyclopentanedione	M3CPT12DONE	765-70-8	C6H8O2	Ketone
112	64,824	73; 88; 41	73	89	MS	2-ethylhexanoic acid	2-Ethyl-Hexanoic Acid	E2HXNOIC	149-57-5	C8H16O2	Carboxylic Acid
113	64,922	69; 121; 41	69	96	RS; MS	1-(2,6,6-trimethylcyclohexa-1,3-dien-1-yl)but-2-en-1-one	β-Damascenone	DAMSNONE	23726-93-4	C13H18O	Terpenoid (Ketone)
114	65,265	88; 101; 73	88	80	MS	ethyl undecanoate	Ethyl Ester Undecanoic Acid	EESTUNDA	627-90-7	C13H26O2	Ester
115	65,660	43; 41; 73	43	79	MS	2-methylpropanoic acid	2-Methyl-Propanoic Acid	M2PPOICA	79-31-2	C4H8O2	Carboxylic Acid
116	65,986	128; 57; 85	128	77	MS	1,3-diazinane-2,4,6-trione	2,4,6-Trihydroxypyrimidine	THDXYPYMNE	67-52-7	C4H4N2O3	Nitrogen
117	66,118	109; 124, 81	109	90	MS	4-methoxyphenol	Mequinol	MEQNOL	150-76-5	C7H8O2	Phenol (Ether)
118	66,384	114; 58; 57	114	84	MS	2,2-diethyl-3-methyl-1,3-oxazolidine	2,2-Diethyl-3-Methyl-Oxazolidine	DEMOXZDNE		C8H17NO	Nitrogen
119	66,461	109; 152, 52	109	82	MS	1,7-dihydropyrazolo[3,4-d]pyrimidine-4,6-dione	Oxypurinol	OXYPUROL	2465-59-0	C5H4N4O2	Nitrogen
120	66,688	79; 108; 77	79	92	MS	phenylmethanol	Benzenemethanol	BENZMTOL	100-51-6	C7H8O	Benzene (Alcohol)
121	67,106	69; 41; 39	69	87	MS	3-methyl-2H-furan-5-one	4-Methyl-2(5H)-Furanone	M4FUR2ONE	6124-79-4	C5H6O2	Furan (Lactone)
122	67,293	126; 55; 83	126	95	MS	3-ethyl-2-hydroxycyclopent-2-en-1-one	3-Ethyl-2-Hydroxy-2-Cyclopenten-1-one	E3H2C2PT1ONE	21835-01-8	C7H10O2	Ketone (Alcohol)
123	68,036	91; 92; 122	91	78	MS	2-phenylethanol	Benzeneethanol	BENZETOL	60-12-8	C8H10O	Benzene (Alcohol)
124	68,580	44; 71; 55	44	80	RS; MS	prop-2-enamide	Acrylamide	ACRYLMDE	79-06-1	C3H5NO	Nitrogen
125	68,698	61; 44; 43	61	79	MS	(2S,3R)-butane-1,2,3,4-tetrol	Erythritol	ERYTOL	149-32-6	C4H10O4	Alcohol (Polyol)

126	68,890	112; 111; 39	112	80	MS	2,3-dimethyl-1H-pyrazol-5-one	2,3-Dimethyl-3-Pyrazolin-5-one	DM23PYZLONE	3201-28-3	C5H8N2O	Nitrogen
127	69,323	136; 121; 65	136	87	MS	(E)-4-(furan-2-yl)but-3-en-2-one	Furfural Acetone	FURALTONE	623-15-4	C8H8O2	Furan (Ketone)
128	69,469	112; 69; 55	112	85	MS	2-hydroxy-3-methylcyclopent-2-en-1-one	Cyclotene	CYTENE	80-71-7	C6H8O2	Ketone (Alcohol)
129	69,557	124; 95; 107	124	80	MS	2-methylbenzene-1,4-diol	2-Methyl-1,4-Benzenediol	M2BNZ14DIOL	95-71-6	C7H8O2	Benzene (Alcohol)
130	69,706	73; 60; 41	73	77	RS; MS	heptanoic Acid	Heptanoic Acid	HPTOIC	111-14-8	C7H14O2	Carboxylic Acid
131	69,883	126; 71; 43	126	95	RS; MS	3-hydroxy-2-methylpyran-4-one	Maltol	MALTOL	118-71-8	C6H6O3	Pyran
132	70,149	94; 109; 66	94	95	MS	1-(1H-pyrrol-2-yl)ethanone	2-Acetylpyrrole	ACTLPYROLE	1072-83-9	C6H7NO	Nitrogen (Pyrrole)
133	70,504	124; 123; 95	124	91	MS	furan-2,5-dicarbaldehyde	2,5-Furandicarboxaldehyde	FUR25DIAL	823-82-5	C6H4O3	Furan (Aldehyde)
134	70,953	112; 55; 84	112	80	MS	3H-pyran-2,6-dione	2H-Pyran-2,6(3H)-Dione	PYR26DIONE	5926-95-4	C5H4O3	Pyran
135	71,427	95; 126; 67	95	84	MS	1,3-bis(furan-2-yl)-1,3-dihydroxypropan-2-one	1-(2-Furanyl)-2-Hydroxy-Ethanone	H2FURYLONE	17678-19-2	C6H6O3	Furan (Ketone)
136	71,638	94; 66; 65	94	89	RS; MS	phenol	Phenol	PHEOL	108-95-2	C6H6O	Phenol
137	72,019	95; 94; 66	95	84	MS	1H-pyrrole-2-carbaldehyde	1H-Pyrrole-2-Carboxaldehyde	PYRLE2AL	1003-29-8	C5H5NO	Nitrogen (Pyrrole)
138	72,070	126; 69; 85	126	79	MS	benzene-1,3,5-triol	Phloroglucinol	PHLOGLNOL	108-73-6	C6H6O3	Phenol
139	72,219	43; 57; 128	43	91	MS	4-hydroxy-2,5-dimethylfuran-3-one	Furaneol	FUREOL	3658-77-3	C6H8O3	Furan (Ketone)
140	72,823	60; 73; 43	60	84	RS; MS	octanoic acid	Octanoic Acid	OCTOIC	124-07-2	C8H16O2	Carboxylic Acid
141	73,300	85; 43; 57	85	78	MS	5-acetyloxolan-2-one	5-Acetyl-Dihydro-2(3H)-Furanone	ACYLDHFURONE	29393-32-6	C6H8O3	Furan (Lactone)
142	73,838	81; 140; 53	81	80	MS	3-(furan-2-yl)propanoic acid	2-Furanpropionic Acid	FURPPIONIC	935-13-7	C7H8O3	Furan (Acid)
143	74,038	31; 43; 42, 60	31	83	MS	1,3-dihydroxypropan-2-one	1,3-Dihydroxy-2-Propanone	DHYPPAONE	96-26-4	C3H6O3	Ketone (Alcohol)
144	74,258	85; 43; 41	85	82	MS	[(2S,5R)-5-methyloxolan-2-yl]methanol	Tetrahydro-5-Methyl-2-Furanmethanol	TEHYMFUROL	6126-49-4	C6H12O2	Furan (Alcohol)
145	74,965	43; 55; 114	55	79	MS	4,5-dimethyl-1,3-dioxol-2-one	4,5-Dimethyl Cyclic Vinylene Ester Carbonic Acid	DM45CVYLESTCA	37830-90-3	C5H6O3	Ester (Ketone)
146	76,832	57; 58; 44	57	83	MS	3-hydroxyoxolan-2-one	2-Hydroxy-Dihydro-2(3H)-Furanone	HY2DHFURONE	19444-84-9	C4H6O3	Furan (Lactone)
147	77,493	126; 43; 79	126	87	MS	(5-formylfuran-2-yl)methyl acetate	5-Acetoxymethyl-2-Furfural A	ACTYMFURALA	10551-58-3	C8H8O4	Furan (Aldehyde / Ether)

148	77,713	126; 43; 79	126	90	MS	(5-formylfuran-2-yl)methyl acetate	5-Acetoxymethyl-2-Furfural B	ACTYMFURALB	10551-58-3	C8H8O4	Furan (Aldehyde / Ether)
149	77,842	135; 150; 77	135	86	MS	4-ethenyl-2-methoxyphenol	2-Methoxy-4-Vinyl-Phenol	MXY2VYL4PHEOL	7786-61-0	C9H10O2	Phenol (Ether)
150	78,094	56; 114; 42	56	82	MS	3-methyloxolane-2,4-dione	3-Methyl-2,4(3H,5H)-Furandione	M3FURDIONE	616-02-4	C5H4O3	Furan (Lactone)
151	79,890	43; 42; 144	43	94	MS	3,5-dihydroxy-6-methyl-2,3-dihydropyran-4-one	3-Hydroxy-2,3-Dihydro-Maltol	HX3DH23MALTOL	28564-83-2	C6H8O4	Pyran (Alcohol / Ether)
152	80,260	60; 73; 41	60	91	RS; MS	decanoic acid	Decanoic Acid	DECOIC	334-48-5	C10H20O2	Carboxylic Acid
153	80,469	142; 43; 68	142	83	MS	3,5-dihydroxy-2-methylpyran-4-one	5-Hydroxy-Maltol	HX5MALTOL	1073-96-7	C6H6O4	Pyran (Ether)
154	83,557	120; 91; 119	120	82	MS	2,3-dihydro-1-benzofuran	2,3-Dihydro-Benzofuran	DHBNZFUR	496-16-2	C8H8O	Benzofuran
155	83,819	69; 57; 41	69	97	MS	(1R,3R,6R,7R,9R)-2,5,8-Trioxatricyclo[4.2.1.0~3,7~]nonan-9-ol	1,4:3,6-Dianhydro-.alpha.-d-Glucopyranose	GLUPYROSE	4451-31-4	C6H8O4	Pyran (Derivative)
156	84,222	95; 39; 68	95	90	MS	1H-pyridin-4-one	4-Pyridinol	PYRDINOL	626-64-2	C5H5NO	Nitrogen
157	84,511	109; 80; 53	109	80	MS	4-aminophenol	4-Amino-Phenol	AMIPHEOL	123-30-8	C6H7NO	Phenol (Amine)
158	85,024	105; 122; 77	105	95	RS; MS	benzoic acid	Benzoic Acid	BNZOIC	65-85-0	C7H6O2	Benzene (Acid)
159	85,202	55; 84; 128	55	78	MS	N-methyl-N-(pentan-3-ylideneamino)methanamine	Dimethylhydrazone 3-Pentanone	DMHDZPTONE	16795-73-6	C7H16N2	Nitrogen (Amine)
160	85,552	60; 73; 43	60	82	RS; MS	undecanoic acid	Undecanoic Acid	UNDECOIC	112-37-8	C11H22O2	Carboxylic Acid
161	86,034	85; 86; 57	85	91	MS	5-(hydroxymethyl)oxolan-2-one	5-(Hydroxymethyl)-Dihydro-2(3H)-Furanone	HM5FURONE	10374-51-3	C5H8O3	Furan (Lactone)
162	86,481	60; 73; 43	60	82	RS; MS	dodecanoic acid	Dodecanoic Acid	DODECOIC	143-07-7	C12H24O2	Carboxylic Acid
163	86,680	97; 126; 41	97	98	RS; MS	5-(hydroxymethyl)furan-2-carbaldehyde	5-(Hydroxymethyl)-2-Furfural	HM5FURAL	67-47-0	C6H6O3	Furan (Aldehyde)
164	87,210	140; 125; 97	140	80	MS	3-methoxybenzene-1,2-diol	3-Methoxy-1,2-Benzenediol	M3BNZDIOL	934-00-9	C7H8O3	Benzene (Alcohol / Ether)
165	89,065	97; 41; 128	97	84	MS	1-(furan-2-yl)ethane-1,2-diol	1-(2-Furanyl)-1,2-Ethanediol	FURYLETDIOL	19377-75-4	C6H8O3	Furan (Alcohol)
166	89,906	44; 74; 102	44	82	MS	4-hydroxyoxolan-2-one	4-Hydroxy-Dihydro-2(3H)-Furanone	DHYHYFURONE	5469-16-9	C4H6O	Furan (Lactone)

[a]RT - Retention Time in minutes.

[b]TI - Target Ion.

[c]MP - Match Percent.

[d]ID - Identification type method used: RS (identified by Reference Standard) and MS (tentatively identified by NIST14 Mass Spectral Library).

ID Number	RT (min)	m/z	Target Ion	Match Percent (%)	IUPAC Name	NIST Database Name	Abbreviations	CAS Number	Molecular Formula	Main Chemical Class
1	4.253	43; 42; 41	43	86	pentane	Pentane	PTANE	109-66-0	C5H12	Hydrocarbon
2	4.518	57; 41; 56	57	84	hexane	Hexane	HXANE	110-54-3	C6H14	Hydrocarbon
3	4.586	67; 68; 53	67	90	penta-1,4-diene	1,4-Pentadiene	PT14DIENE	591-93-5	C5H8	Hydrocarbon
4	4.709	47; 48; 45	47	87	methanethiol	Methanethiol	METHIOL	74-93-1	CH4S	Sulfur
5	4.903	44; 43; 42	44	88	acetaldehyde	Ethanal	ETAL	75-07-0	C2H4O	Aldehyde
6	5.282	47; 62; 45	47	97	methylsulfanylmethane	Dimethyl Sulfide	DMSULFI	75-18-3	C2H6S	Sulfur
7	5.713	58; 57; 59	58	79	propanal	Propanal	PPAL	123-38-6	C3H6O	Aldehyde
8	5.830	68; 39; 40	68	91	furan	Furan	FUR	110-00-9	C4H4O	Furan
9	6.041	43; 41; 72	72	86	2-methylpropanal	2-Methyl-Propanal	MPPAL	78-84-2	C4H8O	Aldehyde
10	6.255	31; 45; 74	31	80	ethyl formate	Ethyl Formate	EESTFA	109-94-4	C3H6O2	Ester
11	6.632	56; 55; 37	56	78	prop-2-enal	2-Propenal	PPENAL	107-02-8	C3H4O	Aldehyde
12	7.199	82; 53; 81	82	94	2-methylfuran	2-Methyl-Furan	M2FUR	534-22-5	C5H6O	Furan
13	7.591	43; 61; 70	43	90	ethyl acetate	Ethyl Acetate	EESTAA	141-78-6	C4H8O2	Ester
14	7.906	43; 72; 57	72	78	butan-2-one	2-Butanone	BT2ONE	78-93-3	C4H8O	Ketone
15	8.338	57; 41; 58	57	86	2-methylbutanal	2-Methyl-Butanal	M2BTAL	96-17-3	C5H10O	Aldehyde
16	8.494	44; 41, 43	41	93	3-methylbutanal	3-Methyl-Butanal	M3BTAL	590-86-3	C5H10O	Aldehyde
17	8.946	31; 45; 46	31	78	ethanol	Ethanol	ETOL	64-17-5	C2H6O	Alcohol
18	9.379	78; 77; 52	78	83	Benzene	Benzene	BNZ	71-43-2	C6H6	Benzene
19	9.569	55; 43; 70	55	80	but-3-en-2-one	3-Buten-2-one	BT3ONE	78-94-4	C4H6O	Ketone
20	10.058	96; 95; 43	96	94	2,5-dimethylfuran	2,5-Dimethyl-Furan	DM25FUR	625-86-5	C6H8O	Furan
21	10.190	57; 75; 102	57	82	ethyl propanoate	Ethyl Propanoate	EESTPA	105-37-3	C5H10O2	Ester
22	11.412	43; 86; 41	86	80	butane-2,3-dione	2,3-Butanedione	BT23DONE	431-03-8	C4H6O2	Ketone
23	12.557	43; 56; 73	56	82	2-methylpropyl acetate	2-Methylpropyl Acetate	MP2ESTAA	110-19-0	C6H12O2	Ester
24	14.801	31; 59; 42	31	80	propan-1-ol	1-Propanol	P1POL	71-23-8	C3H8O	Alcohol
25	14.903	95; 110; 43	95	86	2-ethyl-5-methylfuran	2-Ethyl-5-Methyl-Furan	E2M5FUR	1703-52-2	C7H10O	Furan
26	15.111	91; 92; 65	91	94	toluene	Toluene	TOLNE	108-88-3	C7H8	Benzene

27	15.268	43; 71; 88	88	84	ethyl butanoate	Ethyl Butanoate	EESTBA	105-54-4	C6H12O2	Ester
28	15.327	71; 43; 59	71	78	2-methylbut-3-en-2-ol	2-Methyl-3-Buten-2-ol	M3BT2OL	115-18-4	C5H10O	Alcohol
29	16.166	101; 55; 73	101	82	nonomethyl succinate	Nonomethyl Succinate	METESTBA	3878-55-5	C5H8O4	Ester
30	17.382	43; 57; 100	57	83	pentane-2,3-dione	2,3-Pentanedione	PTDONE	600-14-6	C5H8O2	Ketone
31	18.081	94; 45; 79	94	90	(methyldisulfanyl)methane	Dimethyl Disulfide	DMDSFD	624-92-0	C2H6S2	Sulfur
32	18.284	94; 65; 39	94	78	2-ethenylfuran	2-Ethenyl-Furan	ENYL2FUR	1487-18-9	C6H6O	Furan
33	18.723	81; 99; 55	81	83	2,5-diethyloxolane	2,5-Diethyltetrahydro-Furan	DE25TTHYFUR	41239-48-9	C8H16O	Furan
34	18.905	44; 56; 43	44	94	hexanal	Hexanal	HXAL	66-25-1	C6H12O	Aldehyde
35	21.231	43; 42; 41	43	90	2-methylpropan-1-ol	2-Methyl-1-Propanol	M2PP1OL	78-83-1	C4H10O	Alcohol
36	25.551	85; 41; 56	85	80	5-butyloxolan-2-one	5-Butyl-Dihydro-2(3H)-Furanone	BDH2FURONE	104-50-7	C8H14O2	Furan
37	27.262	108; 107; 77	108	80	3-methylphenol	m-Cresol	MCREOL	108-39-4	C7H8O	Phenol
38	27.966	56; 31; 41	56	80	butan-1-ol	1-Butanol	BT1OL	71-36-3	C4H10O	Alcohol
39	28.085	85; 56; 41	85	82	5-propyloxolan-2-one	5-Propyl-Dihydro-2(3H)-Furanone	PDH2FURONE	105-21-5	C7H12O2	Furan
40	29.054	94; 39; 65	94	87	3-methylpyridazine	3-Methyl-Pyridazine	M3PYRDZNE	1632-76-4	C5H6N2	Nitrogen
41	29.851	43; 58; 71	58	91	heptan-2-one	2-Heptanone	HPT2ONE	110-43-0	C7H14O	Ketone
42	32.080	95; 138; 67	95	85	1-methyl-3-propan-2-ylcyclohexane	m-Menthane	MMTHANE	16580-24-8	C10H20	Terpenoid
43	32.436	80; 53; 52	80	80	pyrimidine	1,3-Diazine	D13ZINE	289-95-2	C4H4N2	Nitrogen
44	32.630	57; 41; 56	57	83	2-methylbutan-1-ol	2-Methyl-1-Butanol	M2BT1OL	137-32-6	C5H12O	Alcohol
45	33.174	82; 53; 81	82	90	2-propylfuran	2-Propyl-Furan	PP2FUR	4229-91-8	C7H10O	Furan
46	34.163	88; 43; 99	88	83	ethyl hexanoate	Ethyl Hexanoate	EESTHA	123-66-0	C8H16O2	Ester
47	34.394	81; 82; 138	81	94	2-pentylfuran	2-Pentyl-Furan	PT2FUR	3777-69-3	C9H14O	Furan
48	36.565	94; 67; 53	94	81	2-methylpyrazine	2-Methyl-Pyrazine	MPYZNE	109-08-0	C5H6N	Nitrogen
49	37.622	43; 72; 100	72	91	2-methyloxolan-3-one	2-Methyl-Dihydro-2(3H)-Furanone	MDH2FURONE	3188-00-9	C5H8O2	Furan
50	37.261	119; 134; 91	119	84	1-methyl-4-propan-2-ylbenzene	p-Cymene	PCYMNE	99-87-6	C10H14	Terpenoid
51	37.369	105; 120; 77	105	95	1,3,5-trimethylbenzene	Mesitylene	MESTLNE	108-67-8	C9H12	Benzene
52	38.007	122; 107; 121	122	80	3,5-dimethylphenol	3,5-Xylenol	XYL35NOL	108-68-9	C8H10O	Phenol
53	38.238	45; 43; 88	45	90	3-hydroxybutan-2-one	3-Hydroxy-2-Butanone	HXY3BT2ONE	513-86-0	C4H8O2	Ketone (Alcohol)
54	38.261	86; 42; 58	86	82	1,3-dioxol-2-one	Vinylene Carbonate	VYLESTCA	96-49-1	C3H4O3	Ester (Ketone)
55	38.738	122; 107; 121	122	82	2,6-dimethylphenol	2,6-Xylenol	XYL26NOL	576-26-1	C8H10O	Phenol

56	39.324	43; 31; 74	43	90	1-hydroxypropan-2-one	1-Hydroxy-2-Propanone	HXY1PP2ONE	116-09-6	C3H6O2	Ketone (Alcohol)
57	40.500	108; 42; 40	108	80	2,5-dimethylpyrazine	2,5-Dimethyl-Pyrazine	DM25PYZNE	123-32-0	C6H8N2	Nitrogen
58	40.910	108; 42; 40	108	90	2,6-dimethylpyrazine	2,6-Dimethyl-Pyrazine	DM26PYZNE	108-50-9	C6H8N2	Nitrogen
59	42.422	82; 39; 54	82	91	cyclopent-2-en-1-one	2-Cyclopenten-1-one	CY2PT1EONE	930-30-3	C5H6O	Ketone
60	41.788	159; 174; 144	159	82	1,2,3,4-tetramethyl-5-prop-1-en-2-ylbenzene	1,2,3,4-Tetramethyl-4-(1-methylethenyl)-Benzene	TM4MEYL1BNZ	61142-76-5	C13H20	Benzene
61	43.239	67; 96; 53	67	83	2-methylcyclopent-2-en-1-one	2-Methyl-2-Cyclopenten-1-one	M2CY2PT1EONE	1120-73-6	C6H8O	Ketone
62	43.444	95; 43; 96	95	80	(5-methylfuran-2-yl)methanethiol	5-Methyl-2-Furanmethanethiol	M2FURTHOL	59303-05-8	C6H8OS	Furan (Sulfur)
63	43.682	57; 88; 56	57	79	1-hydroxybutan-2-one	1-Hydroxy-2-Butanone	H1BT2ONE	5077-67-8	C4H8O2	Ketone (Alcohol)
64	44.389	121; 122; 39	121	80	2-ethyl-6-methylpyrazine	2-Ethyl-6-Methyl-Pyrazine	E6MPYZNE	13925-03-6	C7H10N2	Nitrogen
65	44.691	121; 122; 39	121	77	2-ethyl-5-methylpyrazine	2-Ethyl-5-Methyl-Pyrazine	E5MPYZNE	13360-64-0	C7H10N2	Nitrogen
66	45.234	57; 41; 43	57	93	nonanal	Nonanal	NONAL	124-19-6	C9H18O	Aldehyde
67	45.745	42; 122; 81	122	78	2,3,5-trimethylpyrazine	Trimethyl-Pyrazine	TMPYZNE	14667-55-1	C7H10N2	Nitrogen
68	45.956	67; 95; 124	67	81	(3E)-3-ethyl-2-methylhexa-1,3-diene	3-Ethyl-2-Methyl-1,3-Hexadiene	E3M2HX13DENE	61142-36-7	C9H16	Terpene
69	46.726	68; 96; 40	68	82	cyclohex-2-en-1-one	2-Cyclohexen-1-one	C2HEXONE	930-68-7	C6H8O	Ketone
70	46.901	55; 43; 98	55	87	2-methyl-2H-furan-5-one	5-Methyl-2(3H)-Furanone	M5FURONE	591-12-8	C5H6O2	Furan
71	47.214	98; 42; 69	98	84	4-methyl-1,3-dihydroimidazol-2-one	1,3-Dihydro-4-Methyl-2H-Imidazol-2-one	M4IMDZONE	214-751-9	C4H6N2O	Nitrogen (Ketone)
72	47.569	88; 101; 57	88	89	ethyl octanoate	Ethyl Octanoate	EESTOA	106-32-1	C10H20O2	Ester
73	47.861	159; 174; 131	159	94	1,2,3,4-tetrahydro-1,1,6-trimethylnaphthalene	1,2,3,4-Tetrahydro-1,1,6-Trimethyl-Naphthalene	T116TNP	475-03-6	C13H18	Naphthalene
74	48.559	96; 95; 39	96	96	2-furanaldehyde	Furfural	FURAL	98-01-1	C5H4O2	Furan (Aldehyde)
75	49.502	43; 45; 60	43	90	Acetic Acid	Ethanoic Acid	ETNOIC	64-19-7	C2H4O2	Carboxylic Acid
76	49.783	159; 174; 131	159	96	1,2,3,4-tetrahydro-1,6,8-trimethylnaphthalene	1,2,3,4-Tetrahydro-1,6,8-Trimethyl-Naphthalene	T168TNP	30316-36-0	C13H18	Naphthalene
77	50.004	57; 41; 55	57	82	2-ethylhexan-1-ol	2-Ethyl-1-Hexanol	E2HX1OL	104-76-7	C8H18O	Alcohol
78	50.131	110; 109; 53	110	92	3-methyl-2-furanaldehyde	3-Methyl-Furfural	M3FURAL	33342-48-2	C6H6O2	Furan (Aldehyde)
79	50.259	81; 53; 126	81	82	furan-2-ylmethyl formate	Furfuryl Formate	FURYLFMTE	13493-97-5	C6H6O3	Furan (Ester)
80	50.655	57; 43; 55	57	93	decanal	Decanal	DECAL	112-31-2	C10H2O	Aldehyde
81	50.895	95; 110; 39	95	91	1-(furan-2-yl)ethanone	1-(2-Furanyl)-Ethanone	FURYLONE	1192-62-7	C6H6O2	Furan (Ketone)

82	51.329	106; 105; 77	106	95	benzaldehyde	Benzaldehyde	BENZAL	100-52-7	C7H6O	Benzene (Aldehyde)
83	51.771	81; 98; 52	81	95	furan-2-ylmethyl acetate	Furfuryl Acetate	FURYLACTE	623-17-6	C7H8O3	Furan (Ester)
84	52.336	74; 45; 73	74	90	propanoic acid	Propanoic Acid	PPANOIC	79-09-4	C3H6O2	Carboxylic Acid
85	53.078	63; 78; 45	63	91	methylsulfinylmethane	Dimethyl Sulfoxide	DMSULFO	67-68-5	C2H6OS	Sulfur
86	53.245	159; 174; 131	159	94	1,2,3,4-tetrahydro-1,5,8-trimethylnaphthalene	1,2,3,4-Tetrahydro-1,5,8-Trimethyl-Naphthalene	T158TNP	21693-51-6	C13H18	Naphthalene
87	53.657	110; 109; 53	110	94	5-methyl-2-furanaldehyde	5-Methyl-Furfural	M5FURAL	620-02-0	C6H6O2	Furan (Aldehyde)
88	54.207	96; 42; 68	96	91	cyclopent-4-ene-1,3-dione	4-Cyclopentene-1,3-dione	CPT4E13DONE	930-60-9	C5H4O2	Ketone
89	54.861	131; 132; 103	131	97	2-methyl-1-benzofuran	2-Methyl-Benzofuran	M2BNZFUR	4265-25-2	C9H8O	Benzofuran
90	55.678	109; 124; 81	109	80	1-(5-methylfuran-2-yl)ethanone	1-(5-Methyl-2-Furanyl)-Ethanone	M5FURYLONE	1193-79-9	C7H8O2	Furan (Ketone)
91	55.835	45; 59; 72	45	90	2-(2-ethoxyethoxy)ethanol	Diethylene Glycol Ethyl Ether	DENGOLEETHR	111-90-0	C6H14O3	Ether (Alcohol)
92	56.251	42; 41; 56	42	82	3-methyloxolan-3-one	3-Methyl-Dihydro-2(3H)-Furanone	M3DHFURONE	1679-47-6	C5H8O2	Furan (Lactone)
93	56.693	159; 174; 128	159	86	3,3,5,7-tetramethyl-1,2-dihydroindene	2,3-Dihydro-1,1,4,6-Tetramethyl-1H-Indene	DHT1146MIDNE	941-60-6	C13H18	Indene
94	57.065	91; 92; 120	91	90	2-phenylacetaldehyde	Benzeneacetaldehyde	BENZACETAL	122-78-1	C8H8O	Benzene (Aldehyde)
95	57.117	69; 41; 112	91	87	2,4-dimethyl-2H-furan-5-one	3,5-Dimethyl-Dihydro-2-Furanone	DM35DHFURONE	5584-69-0	C6H8O2	Furan (Lactone)
96	57.323	72; 55; 45	72	78	prop-2-enoic acid	2-Propenoic Acid	PPE2NOIC	79-10-7	C3H4O2	Carboxylic Acid
97	57.337	88; 101; 73	88	89	ethyl decanoate	Ethyl Decanoate	EESTDA	110-38-3	C12H24O2	Ester
98	57.354	105; 77; 51	105	87	1-phenylethanone	Acetophenone	ACETPHONE	98-86-2	C8H8O	Benzene (Ketone)
99	57.744	98; 41; 81	98	98	furan-2-ylmethanol	2-Furanmethanol	FUR2OL	98-00-0	C5H6O2	Furan (Alcohol)
100	57.981	98; 41; 97	97	93	furan-3-ylmethanol	3-Furanmethanol	FUR3OL	4412-91-3	C5H6O2	Furan (Alcohol)
101	58.675	159; 128; 144	159	86	3,3,5,6-tetramethyl-1,2-dihydroindene	2,3-Dihydro-1,1,5,6-Tetramethyl-1H-Indene	DHT1156MIDNE	942-43-8	C13H18	Indene
102	58.897	162; 91; 43	162	91	2-(2-furylmethyl)-5-methylfuran	2-(2-Furanylmethyl)-5-Methyl-Furan	FURYLMFUR	13678-51-8	C10H10O2	Furan
103	60.229	41; 69; 98	41	83	4-methyl-2H-furan-5-one	3-Methyl-2(5H)-Furanone	M3FURONE	22122-36-7	C5H6O2	Furan (Lactone)
104	60.456	95; 112; 43	95	93	(5-methylfuran-2-yl)methanol	5-Methyl-2-Furanmethanol	M5FUR2OL	3857-25-8	C6H8O2	Furan (Alcohol)
105	60.947	108; 91; 90	108	75	benzyl acetate	Benzyl Acetate	PMESTAA	140-11-4	C9H10O2	Benzene (Ester)

106	61.749	55; 84; 54	55	80	2H-furan-5-one	2(5H)-Furanone	FURONE	497-23-4	C4H4O2	Furan (Lactone)
107	62.073	176; 43; 161	176	96	2-methyl-5-[(5-methylfuran-2-yl)methyl]furan	2,2'-Methylenebis[5-Methyl-Furan	MNEB5MFUR	13679-43-1	C10H10O2	Furan
108	62.421	98; 55; 42	98	90	cyclopentane-1,2-dione	1,2-Cyclopentanedione	CPT12DONE	3008-40-0	C5H6O2	Ketone
109	63.971	70; 39; 41	70	84	cyclohex-2-en-1-ol	2-Cyclohexenol	CHEX2E1OL	822-67-3	C6H10O	Alcohol
110	64.539	104; 43; 91	104	84	2-phenylethyl acetate	2-Phenylethyl Acetate	PEESTAA	103-45-7	C10H12O2	Benzene (Ester)
111	64.803	112; 69; 41	112	93	3-methylcyclopentane-1,2-dione	3-Methyl-1,2-Cyclopentanedione	M3CPT12DONE	765-70-8	C6H8O2	Ketone
112	64.824	73; 88; 41	73	89	2-ethylhexanoic acid	2-Ethyl-Hexanoic Acid	E2HXNOIC	149-57-5	C8H16O2	Carboxylic Acid
113	64.922	69; 121; 41	69	96	1-(2,6,6-trimethylcyclohexa-1,3-dien-1-yl)but-2-en-1-one	β-Damascenone	DAMSNONE	23726-93-4	C13H18O	Terpenoid (Ketone)
114	65.265	88; 101; 73	88	80	ethyl undecanoate	Ethyl Undecanoate	EESTUNDA	627-90-7	C13H26O2	Ester
115	65.660	43; 41; 73	43	79	2-methylpropanoic acid	2-Methyl-Propanoic Acid	M2PPOICA	79-31-2	C4H8O2	Carboxylic Acid
116	65.986	128; 57; 85	128	77	1,3-diazinane-2,4,6-trione	2,4,6-Trihydroxypyrimidine	THDXYPYMNE	67-52-7	C4H4N2O3	Nitrogen
117	66.118	109; 124, 81	109	90	4-methoxyphenol	Mequinol	MEQNOL	150-76-5	C7H8O2	Phenol (Ether)
118	66.384	114; 58; 57	114	84	2,2-diethyl-3-methyl-1,3-oxazolidine	2,2-Diethyl-3-Methyl-Oxazolidine	DEMOXZDNE		C8H17NO	Nitrogen
119	66.461	109; 152, 52	109	82	1,7-dihydropyrazolo[3,4-d]pyrimidine-4,6-dione	Oxypurinol	OXYPUROL	2465-59-0	C5H4N4O2	Nitrogen
120	66.688	79; 108; 77	79	92	phenylmethanol	Benzenemethanol	BENZMTOL	100-51-6	C7H8O	Benzene (Alcohol)
121	67.106	69; 41; 39	69	87	3-methyl-2H-furan-5-one	4-Methyl-2(5H)-Furanone	M4FUR2ONE	6124-79-4	C5H6O2	Furan (Lactone)
122	67.293	126; 55; 83	126	95	3-ethyl-2-hydroxycyclopent-2-en-1-one	3-Ethyl-2-Hydroxy-2-Cyclopenten-1-one	E3H2C2PT1ONE	21835-01-8	C7H10O2	Ketone (Alcohol)
123	68.036	91; 92; 122	91	78	2-phenylethanol	Benzeneethanol	BENZETOL	60-12-8	C8H10O	Benzene (Alcohol)
124	68.580	44; 71; 55	44	80	prop-2-enamide	Acrylamide	ACRYLMDE	79-06-1	C3H5NO	Nitrogen
125	68.698	61; 44; 43	61	79	(2S,3R)-butane-1,2,3,4-tetrol	Erythritol	ERYTOL	149-32-6	C4H10O4	Alcohol (Polyol)
126	68.890	112; 111; 39	112	80	2,3-dimethyl-1H-pyrazol-5-one	2,3-Dimethyl-3-Pyrazolin-5-one	DM23PYZLONE	3201-28-3	C5H8N2O	Nitrogen
127	69.323	136; 121; 65	136	87	(E)-4-(furan-2-yl)but-3-en-2-one	Furfural Acetone	FURALTONE	623-15-4	C8H8O2	Furan (Ketone)
128	69.469	112; 69; 55	112	85	2-hydroxy-3-methylcyclopent-2-en-1-one	Cyclotene	CYTENE	80-71-7	C6H8O2	Ketone (Alcohol)
129	69.557	124; 95; 107	124	80	2-methylbenzene-1,4-diol	2-Methyl-1,4-Benzenediol	M2BNZ14DIOL	95-71-6	C7H8O2	Benzene (Alcohol)

130	69.706	73; 60; 41	73	77	heptanoic Acid	Heptanoic Acid	HPTOIC	111-14-8	C7H14O2	Carboxylic Acid
131	69.883	126; 71; 43	126	95	3-hydroxy-2-methylpyran-4-one	Maltol	MALTOL	118-71-8	C6H6O3	Pyran
132	70.149	94; 109; 66	94	95	1-(1H-pyrrol-2-yl)ethanone	2-Acetylpyrrole	ACTLPYROLE	1072-83-9	C6H7NO	Nitrogen (Pyrrole)
133	70.504	124; 123; 95	124	91	furan-2,5-dicarbaldehyde	2,5-Furandicarboxaldehyde	FUR25DIAL	823-82-5	C6H4O3	Furan (Aldehyde)
134	70.953	112; 55; 84	112	80	3H-pyran-2,6-dione	2H-Pyran-2,6(3H)-Dione	PYR26DIONE	5926-95-4	C5H4O3	Pyran
135	71.427	95; 126; 67	95	84	1,3-bis(furan-2-yl)-1,3-dihydroxypropan-2-one	1-(2-Furanyl)-2-Hydroxy-Ethanone	H2FURYLONE	17678-19-2	C6H6O3	Furan (Ketone)
136	71.638	94; 66; 65	94	89	phenol	Phenol	PHEOL	108-95-2	C6H6O	Phenol
137	72.019	95; 94; 66	95	84	1H-pyrrole-2-carbaldehyde	1H-Pyrrole-2-Carboxaldehyde	PYRLE2AL	1003-29-8	C5H5NO	Nitrogen (Pyrrole)
138	72.070	126; 69; 85	126	79	benzene-1,3,5-triol	Phloroglucinol	PHLOGLNOL	108-73-6	C6H6O3	Phenol
139	72.219	43; 57; 128	43	91	4-hydroxy-2,5-dimethylfuran-3-one	Furaneol	FUREOL	3658-77-3	C6H8O3	Furan (Ketone)
140	72.823	60; 73; 43	60	84	octanoic acid	Octanoic Acid	OCTOIC	124-07-2	C8H16O2	Carboxylic Acid
141	73.300	85; 43; 57	85	78	5-acetyloxolan-2-one	5-Acetyl-Dihydro-2(3H)-Furanone	ACYLDHFURONE	29393-32-6	C6H8O3	Furan (Lactone)
142	73.838	81; 140; 53	81	80	3-(furan-2-yl)propanoic acid	2-Furanpropionic Acid	FURPPIONIC	935-13-7	C7H8O3	Furan (Acid)
143	74.038	31; 43; 42, 60	31	83	1,3-dihydroxypropan-2-one	1,3-Dihydroxy-2-Propanone	DHYPPAONE	96-26-4	C3H6O3	Ketone (Alcohol)
144	74.258	85; 43; 41	85	82	[(2S,5R)-5-methyloxolan-2-yl]methanol	Tetrahydro-5-Methyl-2-Furanmethanol	TEHYMFUROL	6126-49-4	C6H12O2	Furan (Alcohol)
145	74.965	43; 55; 114	55	79	4,5-dimethyl-1,3-dioxol-2-one	4,5-Dimethyl Vinylene Carbonate	DM45VYLESTCA	37830-90-3	C5H6O3	Ester (Ketone)
146	76.832	57; 58; 44	57	83	3-hydroxyoxolan-2-one	2-Hydroxy-Dihydro-2(3H)-Furanone	HY2DHFURONE	19444-84-9	C4H6O3	Furan (Lactone)
147	77.493	126; 43; 79	126	87	(5-formylfuran-2-yl)methyl acetate	5-Acetoxymethyl-2-Furfural A	ACTYMFURALA	10551-58-3	C8H8O4	Furan (Aldehyde / Ether)
148	77.713	126; 43; 79	126	90	(5-formylfuran-2-yl)methyl acetate	5-Acetoxymethyl-2-Furfural B	ACTYMFURALB	10551-58-3	C8H8O4	Furan (Aldehyde / Ether)
149	77.842	135; 150; 77	135	86	4-ethenyl-2-methoxyphenol	2-Methoxy-4-Vinyl-Phenol	MXY2VYL4PHEOL	7786-61-0	C9H10O2	Phenol (Ether)
150	78.094	56; 114; 42	56	82	3-methyloxolane-2,4-dione	3-Methyl-2,4(3H,5H)-Furandione	M3FURDIONE	616-02-4	C5H4O3	Furan (Lactone)
151	79.890	43; 42; 144	43	94	3,5-dihydroxy-6-methyl-2,3-dihydropyran-4-one	3-Hydroxy-2,3-Dihydro-Maltol	HX3DH23MALTOL	28564-83-2	C6H8O4	Pyran (Alcohol / Ether)
152	80.260	60; 73; 41	60	91	decanoic acid	Decanoic Acid	DECOIC	334-48-5	C10H20O2	Carboxylic Acid

153	80.469	142; 43; 68	142	83	3,5-dihydroxy-2-methylpyran-4-one	5-Hydroxy-Maltol	HX5MALTOL	1073-96-7	C6H6O4	Pyran (Ether)
154	83.557	120; 91; 119	120	82	2,3-dihydro-1-benzofuran	2,3-Dihydro-Benzofuran	DHBNZFUR	496-16-2	C8H8O	Benzofuran
155	83.819	69; 57; 41	69	97	(1R,3R,6R,7R,9R)-2,5,8-Trioxatricyclo[4.2.1.0~3,7~]nonan-9-ol	1,4:3,6-Dianhydro-.alpha.-d-Glucopyranose	GLUPYROSE	4451-31-4	C6H8O4	Pyran (Derivative)
156	84.222	95; 39; 68	95	90	1H-pyridin-4-one	4-Pyridinol	PYRDINOL	626-64-2	C5H5NO	Nitrogen
157	84.511	109; 80; 53	109	80	4-aminophenol	4-Amino-Phenol	AMIPHEOL	123-30-8	C6H7NO	Phenol (Amine)
158	85.024	105; 122; 77	105	95	benzoic acid	Benzoic Acid	BNZOIC	65-85-0	C7H6O2	Benzene (Acid)
159	85.202	55; 84; 128	55	78	N-methyl-N-(pentan-3-ylideneamino)methanamine	Dimethylhydrazone 3-Pentanone	DMHDZPTONE	16795-73-6	C7H16N2	Nitrogen (Amine)
160	85.552	60; 73; 43	60	82	undecanoic acid	Undecanoic Acid	UNDECOIC	112-37-8	C11H22O2	Carboxylic Acid
161	86.034	85; 86; 57	85	91	5-(hydroxymethyl)oxolan-2-one	5-(Hydroxymethyl)-Dihydro-2(3H)-Furanone	HM5FURONE	10374-51-3	C5H8O3	Furan (Lactone)
162	86.481	60; 73; 43	60	82	dodecanoic acid	Dodecanoic Acid	DODECOIC	143-07-7	C12H24O2	Carboxylic Acid
163	86.680	97; 126; 41	97	98	5-(hydroxymethyl)furan-2-carbaldehyde	5-(Hydroxymethyl)-2-Furfural	HM5FURAL	67-47-0	C6H6O3	Furan (Aldehyde)
164	87.210	140; 125; 97	140	80	3-methoxybenzene-1,2-diol	3-Methoxy-1,2-Benzenediol	M3BNZDIOL	934-00-9	C7H8O3	Benzene (Alcohol / Ether)
165	89.065	97; 41; 128	97	84	1-(furan-2-yl)ethane-1,2-diol	1-(2-Furanyl)-1,2-Ethanediol	FURYLETDIOL	19377-75-4	C6H8O3	Furan (Alcohol)
166	89.906	44; 74; 102	44	82	4-hydroxyoxolan-2-one	4-Hydroxy-Dihydro-2(3H)-Furanone	DHYHYFURONE	5469-16-9	C4H6O	Furan (Lactone)

Table S3A. Mean and relative standard deviation values of identified VOCs in sugarcane-based syrups samples from CERT group.

Volatile Organic Compound	Relative Peak Areas (x 10^2)													
	CERT													
	FRS07		FRS13		FRS14		FRS15		FRS16		FRS17		FRS18	
	Mean	RSD (%)	Mean	RSD (%)	Mean	RSD (%)	Mean	RSD (%)	Mean	RSD (%)	Mean	RSD (%)	Mean	RSD (%)
Pentane	98.7	1.3	28.9	11.0	21.0	0.8	19.0	11.8	52.2	1.8	47.9	6.2	60.5	4.4
Hexane	259.4	2.2	105.1	9.9	61.8	2.3	77.8	1.6	142.7	2.6	99.3	5.7	167.5	1.9
1,4-Pentadiene	0.0		0.0		0.0		0.0		0.0		0.0		0.0	
Methanethiol	1022.5	6.5	134.3	12.0	123.2	2.8	208.9	11.3	163.4	2.9	448.0	7.4	318.0	3.3
Ethanal	13128.8	6.6	1904.3	10.5	3153.9	7.1	3942.1	1.7	8786.9	3.9	8939.0	9.4	12257.1	9.3
Dimethyl Sulfide	7542.8	8.6	643.3	10.5	1135.7	2.1	894.2	5.3	1378.0	7.3	2272.4	8.0	1569.8	6.2
Propanal	412.8	2.4	334.8	11.7	424.8	8.2	293.4	11.0	753.7	4.3	495.0	12.5	1390.6	11.5
Furan	1810.2	2.7	1160.9	6.5	530.8	1.0	593.1	1.0	2149.9	12.7	6029.2	0.2	4240.4	7.2
2-Methyl-Propanal	34308.8	11.7	8319.7	2.3	8395.9	5.3	6454.5	3.5	11410.9	5.8	17659.5	7.9	21889.5	8.7
Ethyl Formate	2461.8	17.7	1922.9	2.3	757.1	14.2	894.4	5.3	7155.3	5.1	2667.3	2.1	0.0	
2-Propenal	234.5	2.1	245.3	12.8	149.8	3.1	400.3	0.3	572.5	1.0	310.2	6.9	353.4	13.2
2-Methyl-Furan	5413.8	8.0	1691.1	1.3	701.9	11.0	708.2	9.7	2031.0	11.0	2646.0	10.7	6988.8	6.3
Ethyl Acetate	7851.5	10.5	7412.5	1.4	5835.8	0.3	2801.0	5.8	25389.2	1.8	13187.9	7.0	280.0	4.6
2-Butanone	4215.0	9.4	231.7	13.3	540.0	9.1	404.9	3.7	217.2	12.9	1133.4	7.4	988.7	1.6
2-Methyl-Butanal	33453.8	15.1	21469.2	0.5	15687.0	0.7	12966.1	3.7	19612.8	1.2	30163.3	8.7	37630.2	9.5
3-Methyl-Butanal	26013.0	6.9	12150.8	2.8	7858.3	8.9	7073.4	9.5	14668.8	5.7	31870.8	4.7	41914.2	13.0
Ethanol	202215.3	7.3	121001.6	1.0	99323.0	0.3	107855.0	4.4	115261.9	3.6	145024.3	1.6	159606.9	2.9
Benzene	161.1	5.5	73.5	0.7	18.0	5.4	53.8	1.5	101.2	9.1	84.4	5.9	200.4	4.3
3-Buten-2-one	570.3	17.3	894.1	8.3	267.5	1.5	341.5	3.4	872.8	5.1	5111.2	3.4	5047.9	5.4
2,5-Dimethyl-Furan	515.4	6.3	907.0	2.6	824.3	10.6	880.9	6.2	3919.7	9.0	2021.6	6.6	1956.1	4.6
Ethyl Propanoate	594.9	12.1	361.7	1.9	28.0	11.4	132.7	11.3	1881.4	4.4	307.1	8.6	0.0	
2,3-Butanedione	31364.2	6.8	16140.5	2.6	1754.9	3.4	2814.3	7.2	1397.2	13.2	33123.4	11.0	49665.1	11.3
2-Methylpropyl Acetate	0.0		0.0		0.0		0.0		0.0		0.0		0.0	
1-Propanol	17.5	7.8	55.0	13.7	54.5	1.9	89.0	2.2	38.8	13.4	92.1	7.1	0.0	
2-Ethyl-5-Methyl-Furan	45.8	7.6	89.6	7.6	28.0	12.1	240.6	10.8	544.9	9.3	350.0	11.4	340.4	0.4
Toluene	2074.3	11.4	5078.0	8.1	4285.8	10.8	4721.7	9.8	19603.0	8.9	53948.0	9.8	651.6	2.2

Ethyl Butanoate	0.0		0.0		0.0		0.0		0.0		0.0		0.0	
2-Methyl-3-Buten-2-ol	2976.6	7.4	0.0		0.0		0.0		0.0		0.0		0.0	
Nonomethyl Succinate	3520.9	7.4	332.6	9.3	45.6	14.0	30.5	16.3	477.6	11.6	1202.6	5.2	0.0	
2,3-Pentanedione	4322.8	13.0	4040.4	1.0	5621.8	13.2	2025.1	11.2	886.0	6.1	3204.1	12.5	17911.1	2.4
Dimethyl Disulfide	0.0		37.5	13.6	250.0	13.9	46.6	0.8	322.5	1.0	302.9	1.5	0.0	
2-Ethenyl-Furan	267.2	8.6	48.3	7.9	141.6	9.0	27.9	9.2	106.8	4.6	263.5	5.5	185.8	1.6
2,5-Diethyltetrahydro-Furan	1238.2	15.7	0.0		0.0		0.0		0.0		0.0		0.0	
Hexanal	0.0		0.0		0.0		0.0		0.0		0.0		0.0	
2-Methyl-1-Propanol	83.9	7.0	2613.3	0.2	1033.2	10.7	23.1	13.4	19137.9	10.8	2101.6	1.1	0.0	
5-Butyl-Dihydro-2(3H)-Furanone	9084.9	11.1	1375.7	4.4	975.3	1.0	506.1	1.7	1645.1	1.4	1942.4	6.1	1114.9	9.6
m-Cresol	2042.9	4.7	169.2	12.8	238.8	14.6	269.3	4.1	368.4	15.0	2300.9	5.2	3490.7	8.4
1-Butanol	125.1	8.8	18.5	9.5	40.6	12.3	34.9	1.0	1044.9	5.3	408.2	14.4	105.6	4.6
5-Propyl-Dihydro-2(3H)-Furanone	2635.8	5.8	642.2	10.6	300.4	9.9	274.4	0.8	1009.7	1.5	1047.0	7.3	536.6	5.0
3-Methyl-Pyridazine	3329.2	5.0	1590.5	4.1	2096.3	0.8	2183.3	13.2	3201.6	8.1	4764.7	11.2	8754.7	4.1
2-Heptanone	0.0		0.0		0.0		0.0		0.0		0.0		0.0	
m-Menthane	139.5	11.6	3.5	11.3	9.0	14.4	7.1	9.7	16.9	2.2	27.3	16.0	63.7	10.2
1,3-Diazine	171.6	7.7	23.2	13.5	22.1	6.1	27.2	11.1	325.7	12.0	62.5	12.3	150.1	2.9
2-Methyl-1-Butanol	1953.7	11.5	8135.5	4.9	5740.1	6.8	4709.4	1.8	21874.3	8.6	14512.1	11.9	0.0	
2-Propyl-Furan	1425.1	6.4	397.5	2.0	552.3	16.2	83.9	8.0	2172.7	9.4	105.7	11.8	3291.2	11.8
Ethyl Hexanoate	0.0		0.0		0.0		0.0		0.0		0.0		0.0	
2-Pentyl-Furan	12.9	6.6	2.6	10.8	3.7	8.9	110.9	5.8	13.0	5.8	19.4	1.9	12.0	10.3
2-Methyl-Pyrazine	1015.8	7.6	213.5	12.0	1265.2	15.5	1014.2	6.9	1080.1	3.1	2077.3	10.6	454.2	2.2
2-Methyl-Dihydro-2(3H)-Furanone	34004.1	1.5	17299.6	1.8	13953.8	13.9	11415.6	8.6	15831.7	7.7	21675.5	7.5	19081.9	11.8
p-Cymene	39.2	2.5	49.7	10.1	57.7	2.3	49.6	10.4	120.3	0.5	20.7	5.8	58.5	7.3
Mesitylene	22.6	1.9	6.4	2.1	3.2	13.9	3.5	9.4	3436.8	11.6	14.9	9.7	10.2	5.3
3,5-Xylenol	148.7	6.8	49.1	11.6	81.3	6.8	97.3	8.5	158.9	5.7	286.1	9.3	350.1	13.2
3-Hydroxy-2-Butanone	4692.4	4.1	1264.0	1.1	1671.9	8.8	1477.8	7.2	2171.8	13.4	3354.1	8.4	5088.3	6.7
Vinylene Carbonate	25513.2	6.7	4045.3	1.3	7828.1	12.9	9172.5	9.7	16120.0	4.0	19420.9	12.7	21659.4	10.7
2,6-Xylenol	137.0	13.8	217.9	7.2	265.8	13.9	480.3	4.2	648.1	3.0	1249.9	8.2	1624.2	3.4
1-Hydroxy-2-Propanone	14507.6	11.3	5406.5	0.9	10358.2	0.4	6411.6	1.6	13091.7	5.4	28531.5	7.0	21972.9	10.8
2,5-Dimethyl-Pyrazine	64.0	12.1	45.8	15.1	1523.4	0.4	41.8	12.8	1053.3	4.8	149.4	2.2	1178.3	13.8
2,6-Dimethyl-Pyrazine	3332.6	3.5	1301.8	12.5	1474.2	4.0	1374.8	1.1	1075.6	5.0	1368.4	5.3	1308.0	2.8
2-Cyclopenten-1-one	5688.4	4.8	1141.1	3.1	1669.0	10.2	1692.7	1.4	1387.8	1.8	3467.5	0.5	3582.4	12.3
1,2,3,4-Tetramethyl-4-(1-methylethenyl)-Benzene	0.0		0.0		0.0		0.0		0.0		0.0		0.0	

2-Methyl-2-Cyclopenten-1-one	2219.1	1.8	332.6	2.9	624.1	2.7	485.5	2.9	1094.2	11.9	1534.2	7.8	1830.1	5.4
5-Methyl-2-Furanmethanethiol	695.8	14.2	3004.1	10.5	1084.2	6.4	1428.4	1.3	8526.8	0.9	2656.3	5.7	27.9	9.2
1-Hydroxy-2-Butanone	7661.4	12.3	1482.4	3.8	2305.3	4.1	3674.1	1.9	656.2	4.7	7972.4	1.6	9836.8	10.8
2-Ethyl-6-Methyl-Pyrazine	386.2	12.1	128.0	8.9	177.9	2.6	138.2	2.5	130.3	11.7	181.4	6.5	144.0	6.9
2-Ethyl-5-Methyl-Pyrazine	0.0		0.0		0.0		0.0		0.0		0.0		0.0	
Nonanal	984.8	13.5	637.1	1.0	643.0	10.8	508.3	7.2	681.2	5.2	1376.7	15.2	2511.5	5.6
Trimethyl-Pyrazine	0.0		0.0		0.0		0.0		0.0		0.0		0.0	
3-Ethyl-2-Methyl-1,3-Hexadiene	0.0		0.0		0.0		0.0		0.0		0.0		0.0	
2-Cyclohexen-1-one	41.6	13.4	15.0	13.4	10.0	14.5	56.4	6.1	587.2	3.1	746.9	6.2	31.8	13.7
5-Methyl-2(3H)-Furanone	12211.5	6.5	3164.5	9.5	5377.5	10.8	5683.0	3.9	8085.5	2.0	10002.6	1.5	8980.7	5.3
1,3-Dihydro-4-Methyl-2H-Imidazol-2-one	4383.4	0.3	1450.9	7.8	1885.4	1.0	1816.1	7.9	3910.9	1.8	4005.1	1.2	6696.1	9.9
Ethyl Octanoate	0.0		0.0		0.0		0.0		0.0		0.0		0.0	
1,2,3,4-Tetrahydro-1,1,6-Trimethyl-Naphthalene	16.2	5.3	267.2	6.6	246.3	14.9	192.8	2.0	483.1	2.7	694.6	4.7	370.7	8.4
Furfural	40114.7	3.1	25043.5	2.4	26413.9	1.6	24513.0	6.4	26387.8	10.5	32952.1	2.7	34806.3	14.1
Ethanoic Acid	622455.5	2.2	317668.4	3.2	349871.7	2.9	66429.6	5.0	76709.6	9.1	191576.4	0.8	194203.0	2.0
1,2,3,4-Tetrahydro-1,6,8-Trimethyl-Naphthalene	830.2	4.8	555.3	2.4	794.6	4.9	447.6	1.0	670.1	2.8	1265.3	15.7	606.5	5.1
2-Ethyl-1-Hexanol	783.3	7.0	593.1	5.6	552.3	4.6	392.4	12.2	1777.5	0.0	2997.0	4.4	855.9	8.7
3-Methyl-Furfural	569.9	4.2	168.2	0.2	230.6	14.0	275.2	5.0	514.6	4.3	455.5	10.0	848.6	7.2
Furfuryl Formate	1604.2	9.3	869.2	13.0	1344.4	14.4	1820.2	10.4	3999.8	8.9	2991.8	0.5	4359.7	11.4
Decanal	1724.8	7.3	922.2	1.1	78.0	4.5	250.3	0.8	1242.0	8.6	2215.3	13.5	3199.5	10.7
1-(2-Furanyl)-Ethanone	15678.7	3.8	10543.7	2.3	11113.3	8.1	10890.1	3.1	14842.7	4.5	14116.0	5.9	15542.0	13.8
Benzaldehyde	1868.9	2.8	895.8	5.2	562.4	17.7	611.7	7.8	675.0	1.2	1375.2	5.9	1623.6	1.8
Furfuryl Acetate	2230.8	4.5	592.7	12.7	938.8	16.7	1123.8	6.1	2237.2	8.4	2016.1	5.0	3456.7	1.1
Propanoic Acid	734.6	4.6	226.4	3.3	220.6	6.2	2314.0	6.0	6815.4	7.8	299.8	5.8	701.6	13.0
Dimethyl Sulfoxide	659.5	16.5	49.1	12.5	306.0	9.7	435.3	3.3	441.0	4.4	514.9	8.1	267.6	12.3
1,2,3,4-Tetrahydro-1,5,8-Trimethyl-Naphthalene	444.0	3.5	215.7	13.5	293.5	8.8	169.3	1.5	417.8	11.7	571.3	10.9	118.3	4.3
5-Methyl-Furfural	21904.3	10.5	12916.4	9.7	14819.6	1.1	17003.7	4.2	17848.8	5.6	18869.5	2.1	22090.1	11.1
4-Cyclopentene-1,3-dione	20612.7	13.7	12474.9	10.2	15726.9	2.6	13308.5	8.6	17196.2	6.4	20881.1	2.1	21389.0	8.8
2-Methyl-Benzofuran	13073.7	14.1	4250.3	8.3	2762.1	5.9	2345.0	6.0	8254.9	1.6	9803.4	6.9	8989.3	0.6
1-(5-Methyl-2-Furanyl)-Ethanone	3859.6	13.6	558.6	10.6	662.8	8.1	1012.7	5.4	2278.6	3.5	2243.6	8.0	2693.8	5.3
Diethylene Glycol Ethyl Ether	3325.1	0.3	1713.8	3.6	1815.2	5.9	2017.9	5.4	1868.5	11.4	5243.0	10.1	5155.3	13.4
3-Methyl-Dihydro-2(3H)-Furanone	1730.1	10.1	765.8	13.5	869.4	14.4	863.8	7.4	1738.1	2.1	1413.1	8.4	1932.1	7.7
2,3-Dihydro-1,1,4,6-Tetramethyl-1H-Indene	48.6	13.4	44.2	10.6	62.5	2.6	31.7	7.2	81.9	4.4	130.8	4.4	81.7	7.6
Benzeneacetaldehyde	17699.7	8.2	776.3	6.4	972.7	5.3	3591.8	4.3	8725.4	5.7	9758.5	6.4	12558.9	11.9

3,5-Dimethyl-Dihydro-2-Furanone	1745.3	12.9	512.9	17.0	662.5	2.1	614.7	10.6	540.0	3.0	3382.9	1.0	1552.4	8.3
2-Propenoic Acid	2598.8	7.4	45.3	11.4	3912.6	13.2	3090.4	8.7	13197.2	2.3	6314.2	1.4	11803.3	8.6
Ethyl Decanoate	0.0		0.0		0.0		0.0		0.0		0.0		0.0	
Acetophenone	577.9	0.2	121.0	8.4	123.7	12.7	184.2	12.3	313.8	12.7	300.7	3.0	786.4	12.5
2-Furanmethanol	28914.3	9.8	21515.7	0.3	24661.9	3.0	18617.5	2.7	21685.3	7.5	23273.4	11.9	52489.4	8.4
3-Furanmethanol	49757.8	1.0	27453.7	11.7	35307.1	13.4	22481.4	7.7	16348.3	6.7	18357.0	11.2	64212.9	7.2
2,3-Dihydro-1,1,5,6-Tetramethyl-1H-Indene	274.2	11.5	91.9	9.9	223.7	11.2	287.5	1.9	271.4	11.2	469.1	4.3	529.9	7.7
2-(2-Furanylmethyl)-5-Methyl-Furan	872.0	14.8	129.6	13.9	184.9	15.9	353.0	1.7	708.5	2.5	479.6	10.0	995.9	12.7
3-Methyl-2(5H)-Furanone	594.7	13.9	265.6	7.1	595.2	10.6	579.3	3.8	681.4	9.7	976.3	4.6	985.7	3.5
5-Methyl-2-Furanmethanol	3490.5	10.2	1341.8	7.3	2427.7	18.6	1929.1	6.4	2637.3	11.0	4174.9	0.4	5391.7	12.7
Benzyl Acetate	268.3	8.1	219.5	6.9	44.5	1.7	366.5	7.7	289.6	10.6	236.9	15.0	0.0	
2(5H)-Furanone	32145.9	12.9	14215.7	0.6	14375.6	12.8	19058.4	5.4	7999.2	4.7	37096.8	2.6	27152.0	3.0
2,2'-Methylenebis[5-Methyl-Furan	728.7	11.2	333.1	12.1	505.7	14.5	377.1	2.2	681.3	7.6	1462.2	8.4	1018.0	10.4
1,2-Cyclopentanedione	39093.3	3.2	16481.8	12.7	23452.4	4.2	26782.4	0.9	24006.4	5.9	33818.8	4.7	39417.0	8.6
2-Cyclohexenol	17588.4	6.9	5266.8	3.7	6902.3	4.9	7666.5	5.0	9665.4	2.5	15789.5	3.1	16990.4	4.8
2-Phenylethyl Acetate	597.1	13.8	1309.2	16.8	512.0	11.1	2664.2	6.6	6220.2	1.5	2687.3	12.4	364.5	4.9
3-Methyl-1,2-Cyclopentanedione	24451.5	11.2	6196.1	0.5	7471.7	9.8	9727.3	4.3	11541.9	7.6	18549.0	0.9	21664.8	11.6
2-Ethyl-Hexanoic Acid	0.0		0.0		0.0		0.0		0.0		0.0		0.0	
β-Damascenone	10111.1	8.0	339.6	2.4	201.6	3.2	3300.4	9.6	1127.0	5.7	292.9	4.0	10703.7	5.0
Ethyl Undecanoate	0.0		0.0		0.0		0.0		0.0		0.0		0.0	
2-Methyl-Propanoic Acid	9201.1	16.0	2211.9	1.7	2846.1	4.9	3305.0	7.8	6630.5	9.8	7936.8	6.8	12086.5	10.5
2,4,6-Trihydroxypyrimidine	31204.5	10.5	16976.0	3.4	18202.4	6.5	17663.8	8.2	27315.9	11.7	25867.3	3.7	26105.2	7.7
Mequinol	3005.5	12.2	556.4	3.1	756.6	3.2	931.4	3.3	1039.0	3.1	1573.3	3.2	2222.2	9.7
2,2-Diethyl-3-Methyl-Oxazolidine	6418.8	8.6	2199.8	11.0	2857.8	17.2	3790.2	1.9	7490.0	11.4	9522.3	4.3	13662.4	2.5
Oxypurinol	13727.5	12.7	4555.7	3.7	4692.5	6.5	7792.4	12.8	8884.9	6.7	12480.4	4.6	12342.7	8.5
Benzenemethanol	9833.6	11.0	1495.9	10.8	301.1	6.0	805.1	10.2	704.4	10.5	604.8	0.1	261.2	9.7
4-Methyl-2(5H)-Furanone	5819.4	7.7	1480.4	5.2	2186.2	5.7	2280.4	11.8	3179.6	9.7	4120.6	7.5	6962.8	10.5
3-Ethyl-2-Hydroxy-2-Cyclopenten-1-one	6395.2	14.3	936.9	8.4	1288.2	5.9	1543.9	9.2	2151.0	12.2	3124.1	9.3	3729.0	10.2
Benzeneethanol	58.8	9.4	287.9	2.3	72.7	13.6	194.6	5.4	464.1	12.7	318.8	5.0	178.5	4.3
Acrylamide	5432.4	11.3	1208.8	5.0	2462.7	9.7	2951.1	8.9	6052.4	5.0	6188.3	6.0	6777.6	10.7
Erythritol	13181.9	8.0	3805.6	5.8	4221.0	6.5	4625.2	11.4	8245.1	5.2	13061.5	1.1	17525.1	7.7
2,3-Dimethyl-3-Pyrazolin-5-one	2201.5	4.6	519.9	3.5	725.2	13.1	838.8	5.9	1175.7	6.9	1585.9	12.1	1934.6	12.1
Furfural Acetone	1677.6	13.2	504.4	9.8	683.7	6.7	782.5	10.6	1097.1	10.8	1524.1	0.8	2071.1	7.9
Cyclotene	4710.4	12.4	1578.7	10.1	2362.1	17.5	3217.2	5.3	4823.6	8.3	5438.5	5.0	7902.0	4.7

2-Methyl-1,4-Benzenediol	9547.5	6.0	907.8	4.6	1155.0	11.6	1131.4	4.6	2171.6	11.0	2983.8	3.5	6209.2	5.4
Heptanoic Acid	0.0		0.0		0.0		0.0		0.0		0.0		0.0	
Maltol	23760.6	7.6	8143.1	13.2	10318.0	5.4	12514.7	6.2	15762.4	7.3	17409.2	5.4	20498.4	10.8
2-Acetylpyrrole	28102.8	10.5	7518.7	6.9	8496.4	2.7	7404.8	5.7	9360.2	7.7	14430.4	5.7	14092.6	9.3
2,5-Furandicarboxaldehyde	44213.2	8.7	13933.4	6.3	12905.7	14.9	16453.0	3.7	20916.9	14.1	31055.1	1.6	33001.5	6.5
2H-Pyran-2,6(3H)-Dione	13931.4	7.1	2037.0	6.7	3432.3	18.1	6873.8	11.2	14810.2	5.9	13451.8	6.9	16447.7	6.4
1-(2-Furanyl)-2-Hydroxy-Ethanone	28012.7	11.6	12311.1	4.3	20375.4	8.8	20822.3	12.7	57419.4	0.3	17626.4	1.2	29062.9	6.3
Phenol	11182.0	14.6	1877.7	4.5	2431.7	9.6	2987.2	8.8	4626.4	8.3	5719.0	12.4	8204.7	11.4
1H-Pyrrole-2-Carboxaldehyde	5622.0	10.5	637.0	5.9	872.0	4.2	911.5	3.4	835.0	11.1	5732.3	9.3	5897.6	4.1
Phloroglucinol	13128.8	5.7	2680.8	9.8	4080.1	13.0	5238.3	12.2	14692.0	6.2	12492.0	0.3	16640.6	7.7
Furaneol	17628.1	0.4	14166.2	2.1	15189.2	0.8	18232.8	7.9	13267.4	1.5	15414.6	0.6	15728.2	3.8
Octanoic Acid	189.7	10.0	261.9	8.2	344.2	17.5	403.5	3.0	203.2	14.5	330.1	7.0	383.4	8.7
5-Acetyl-Dihydro-2(3H)-Furanone	17646.6	1.3	11472.5	0.5	12424.4	0.1	11865.3	5.9	11931.0	7.9	17076.3	7.3	15308.2	7.6
2-Furanpropionic Acid	10673.2	10.1	2355.8	6.6	3286.1	11.3	4640.8	5.7	10089.4	8.5	9669.9	1.3	11266.7	6.0
1,3-Dihydroxy-2-Propanone	456536.2	4.1	285396.7	2.8	355341.2	6.1	325658.0	3.0	350592.8	3.7	417470.4	1.4	432011.6	0.0
Tetrahydro-5-Methyl-2-Furanmethanol	148.2	4.4	12061.3	6.8	56.7	2.9	13027.6	3.1	9247.3	1.3	16415.9	2.4	588.9	13.2
4,5-Dimethyl Vinylene Carbonate	19934.1	5.9	6180.3	9.3	8154.2	13.7	9842.0	5.9	6807.3	2.1	16353.7	2.5	16034.3	10.2
2-Hydroxy-Dihydro-2(3H)-Furanone	4070.6	8.5	31046.0	2.2	35819.7	0.7	30587.2	1.2	22563.2	3.5	5312.8	1.4	5574.3	9.1
5-Acetoxymethyl-2-Furfural A	13502.8	7.5	11357.1	6.0	12593.0	7.0	12051.8	10.6	9644.0	2.8	17759.4	6.7	16129.7	11.1
5-Acetoxymethyl-2-Furfural B	19318.5	3.7	12399.1	1.5	13399.5	3.7	15313.4	5.4	17830.9	5.1	21514.3	2.0	18481.1	9.1
2-Methoxy-4-Vinyl-Phenol	4120.0	8.6	817.6	10.5	1235.9	5.1	1441.2	1.6	3060.6	13.6	3382.6	12.2	6340.0	9.2
3-Methyl-2,4(3H,5H)-Furandione	10561.0	9.7	3018.8	3.1	4239.9	8.6	5296.3	3.7	6246.8	9.6	9340.2	6.1	10253.6	10.0
3-Hydroxy-2,3-Dihydro-Maltol	11035.1	7.4	7496.4	11.0	9867.8	8.4	10080.8	9.0	9991.1	8.6	13316.2	13.9	13223.8	0.8
Decanoic Acid	3604.8	0.7	959.2	5.3	884.3	7.9	1697.1	3.0	408.7	12.0	211.6	9.6	799.9	1.9
5-Hydroxy-Maltol	46789.7	13.0	10577.0	10.9	15988.5	14.0	22443.4	3.7	20736.5	8.9	34672.7	4.3	38102.8	9.9
2,3-Dihydro-Benzofuran	3548.8	11.3	852.6	3.7	1228.6	3.3	1486.6	1.8	1690.0	2.3	2987.3	6.2	5430.7	9.4
1,4:3,6-Dianhydro-.alpha.-d-Glucopyranose	8562.3	3.9	2663.7	3.9	2815.7	0.8	4890.1	3.8	4620.5	9.6	8321.9	4.2	7118.6	6.5
4-Pyridinol	16057.9	6.9	3758.5	0.4	5495.9	12.7	6563.0	3.9	8979.0	4.2	12385.3	8.3	13378.4	8.4
4-Amino-Phenol	11355.1	7.5	2040.5	2.2	3431.0	7.8	3681.8	4.6	4643.1	0.4	7835.5	6.7	10392.8	11.8
Benzoic Acid	14984.6	6.5	44.6	7.1	15.6	5.1	1746.9	3.7	1495.7	5.6	2990.6	2.7	2896.9	2.6
Dimethylhydrazone 3-Pentanone	14574.4	6.0	5343.2	9.5	7146.6	3.9	7770.4	5.0	5269.3	0.2	22368.8	0.3	15247.3	12.2
Undecanoic Acid	10566.7	6.6	79.7	9.7	228.5	5.5	1775.2	3.1	161.6	4.5	766.0	1.6	221.6	13.9
5-(Hydroxymethyl)-Dihydro-2(3H)-Furanone	34037.4	12.5	27394.2	5.2	30405.2	6.3	31683.7	8.0	41433.5	3.5	20401.6	4.4	75799.6	11.3
Dodecanoic Acid	3489.1	10.2	74.7	7.9	194.0	4.4	1125.4	3.8	1307.2	6.3	3671.4	5.8	2390.5	13.6

5-(Hydroxymethyl)-2-Furfural	159244.2	5.2	178092.7	0.4	207323.6	4.9	164599.0	4.5	198276.1	10.3	165006.0	1.3	172268.3	5.2
3-Methoxy-1,2-Benzenediol	6999.2	4.2	2006.7	6.8	2443.1	14.8	3640.6	1.4	5633.7	9.9	7682.9	3.9	14099.0	6.8
1-(2-Furanyl)-1,2-Ethanediol	42992.2	1.4	14002.0	10.1	11221.4	8.4	12514.4	2.6	14995.6	10.9	15521.4	0.2	22502.6	10.8
4-Hydroxy-Dihydro-2(3H)-Furanone	27068.9	10.8	6722.6	6.9	9676.9	13.5	13815.3	4.1	19296.8	4.3	23371.3	0.8	29163.2	13.9

[1]RSD - Relative Standard Deviation values.

Table S3B. Mean and relative standard deviation values of identified VOCs in sugarcane-based syrups samples from NCERT group.

Volatile Organic Compound	Relative Peak Areas (x 10^3)					
	NCERT					
	ECAL14		ENCAL14		GLA14	
	Mean	RSD (%)	Mean	RSD (%)	Mean	RSD (%)
Pentane	42.5	4.6	41.7	6.5	122.3	12.6
Hexane	112.2	5.2	69.7	0.5	235.8	3.0
1,4-Pentadiene	0.0		2.6	8.5	0.0	
Methanethiol	494.9	3.9	177.0	2.4	161.3	5.9
Ethanal	4952.9	12.1	48.2	0.2	10061.5	3.0
Dimethyl Sulfide	2901.7	9.6	7643.0	10.1	558.4	8.5
Propanal	827.2	5.1	315.0	6.8	1421.5	6.5
Furan	1982.9	0.9	2777.0	8.9	2118.5	4.8
2-Methyl-Propanal	12951.6	5.0	6195.3	0.7	9333.3	2.4
Ethyl Formate	3245.0	6.9	0.0		2067.2	5.7
2-Propenal	519.3	8.2	27.8	6.6	350.3	5.3
2-Methyl-Furan	2158.4	3.4	166.1	0.2	2450.4	1.6
Ethyl Acetate	10340.2	3.3	8.5	14.9	20056.1	6.9
2-Butanone	382.6	8.5	70.3	8.9	545.4	9.0
2-Methyl-Butanal	23157.1	1.3	8960.3	0.4	14780.3	3.4
3-Methyl-Butanal	28252.9	1.3	1592.0	7.7	8899.9	2.2
Ethanol	59701.7	5.1	47501.2	8.0	3150.3	5.7
Benzene	118.8	3.9	3.7	6.7	35.5	11.6
3-Buten-2-one	1046.7	4.7	46.2	10.5	3489.1	0.5

2,5-Dimethyl-Furan	1296.2	4.3	88.4	5.6	2696.3	9.4
Ethyl Propanoate	0.0		0.0		10327.3	1.0
2,3-Butanedione	11909.1	9.6	212.0	7.4	20966.1	0.9
2-Methylpropyl Acetate	58.4	5.6	0.0		1987.0	7.1
1-Propanol	1677.6	9.7	0.0		10237.8	2.5
2-Ethyl-5-Methyl-Furan	14.8	8.0	0.0		703.9	6.1
Toluene	3899.0	10.5	1258.9	9.0	5073.9	0.4
Ethyl Butanoate	0.0		0.0		1270.8	6.5
2-Methyl-3-Buten-2-ol	840.5	9.0	18.8	0.1	0.0	
Nonomethyl Succinate	324.2	1.2	24.0	8.9	0.0	
2,3-Pentanedione	5060.6	7.7	125.7	3.8	9459.5	6.0
Dimethyl Disulfide	2179.6	4.9	0.0		0.0	
2-Ethenyl-Furan	219.2	2.3	27.7	1.1	35.3	12.1
2,5-Diethyltetrahydro-Furan	210.2	10.2	0.0		0.0	
Hexanal	0.0		13.3	2.9	0.0	
2-Methyl-1-Propanol	18.9	3.9	7.6	4.1	41073.2	2.9
5-Butyl-Dihydro-2(3H)-Furanone	1079.8	11.1	33.4	12.2	15.0	4.4
m-Cresol	132.2	7.3	19.4	3.4	986.8	1.5
1-Butanol	68.4	12.7	0.0		1059.2	2.9
5-Propyl-Dihydro-2(3H)-Furanone	728.4	5.7	9.8	3.5	14.4	8.0
3-Methyl-Pyridazine	776.7	9.3	11.8	2.6	2473.6	3.2
2-Heptanone	8.6	1.9	0.0		0.0	
m-Menthane	0.0		0.0		0.0	
1,3-Diazine	364.4	6.3	1.6	2.5	988.6	4.9
2-Methyl-1-Butanol	5493.2	9.8	976.9	9.1	19225.4	1.0
2-Propyl-Furan	55.7	12.9	7.3	7.0	528.1	7.2
Ethyl Hexanoate	0.0		0.0		295.7	15.2
2-Pentyl-Furan	0.0		10.8	8.1	0.0	
2-Methyl-Pyrazine	1828.5	6.9	36.1	5.7	799.4	1.3
2-Methyl-Dihydro-2(3H)-Furanone	17829.4	5.2	69.7	14.3	2505.0	5.3
p-Cymene	53.9	3.4	0.0		212.6	8.6
Mesitylene	3.8	1.7	0.0		116.7	3.3
3,5-Xylenol	0.0		0.0		88.4	1.3
3-Hydroxy-2-Butanone	715.2	2.8	368.0	12.7	1920.1	2.2

Vinylene Carbonate	4192.5	9.8	83.3	4.5	12628.8	4.7
2,6-Xylenol	186.0	9.2	4.8	4.8	269.9	7.2
1-Hydroxy-2-Propanone	8601.1	13.8	3732.0	1.7	20944.0	2.3
2,5-Dimethyl-Pyrazine	108.5	4.9	94.8	4.8	127.6	4.2
2,6-Dimethyl-Pyrazine	1440.2	4.9	29.4	9.0	997.5	4.2
2-Cyclopenten-1-one	391.5	1.9	17.1	3.2	1074.8	5.6
1,2,3,4-Tetramethyl-4-(1-methylethenyl)-Benzene	0.0		0.0		0.0	
2-Methyl-2-Cyclopenten-1-one	168.8	2.6	21.9	16.7	419.5	3.9
5-Methyl-2-Furanmethanethiol	2029.3	1.3	11.5	3.6	13381.6	3.2
1-Hydroxy-2-Butanone	1318.8	14.3	102.7	3.7	4740.7	2.0
2-Ethyl-6-Methyl-Pyrazine	144.0	9.7	14.1	2.1	31.9	8.5
2-Ethyl-5-Methyl-Pyrazine	0.0		0.0		0.0	
Nonanal	2078.7	5.6	202.0	7.4	472.8	0.4
Trimethyl-Pyrazine	0.0		0.0		0.0	
3-Ethyl-2-Methyl-1,3-Hexadiene	0.0		0.0		0.0	
2-Cyclohexen-1-one	24.0	4.4	98.9	10.9	84.5	12.0
5-Methyl-2(3H)-Furanone	1670.7	4.8	157.5	0.3	4396.5	3.3
1,3-Dihydro-4-Methyl-2H-Imidazol-2-one	861.2	0.5	79.9	9.4	2433.8	0.5
Ethyl Octanoate	0.0		0.0		1247.6	4.7
1,2,3,4-Tetrahydro-1,1,6-Trimethyl-Naphthalene	111.7	4.8	3.4	8.4	70.1	3.6
Furfural	24065.2	7.8	26526.1	8.4	39888.4	7.6
Ethanoic Acid	351640.1	0.1	235969.8	9.2	436448.0	0.7
1,2,3,4-Tetrahydro-1,6,8-Trimethyl-Naphthalene	214.9	6.7	4.9	1.2	357.8	3.4
2-Ethyl-1-Hexanol	543.4	5.0	67.7	6.1	11461.3	2.6
3-Methyl-Furfural	4521.9	6.4	1949.6	0.9	232.6	5.9
Furfuryl Formate	344.6	10.6	54.9	0.6	1513.1	1.2
Decanal	150.9	0.9	82.7	7.8	447.3	11.5
1-(2-Furanyl)-Ethanone	10443.6	9.5	42.1	4.0	15861.4	5.5
Benzaldehyde	402.0	11.8	6.1	13.1	1237.3	1.5
Furfuryl Acetate	211.6	10.9	105.7	1.5	946.5	1.6
Propanoic Acid	905.5	9.3	0.0		6991.8	1.5
Dimethyl Sulfoxide	89.7	6.6	49.9	11.1	313.5	11.9
1,2,3,4-Tetrahydro-1,5,8-Trimethyl-Naphthalene	101.1	9.0	5.3	6.5	299.5	1.9
5-Methyl-Furfural	5231.7	10.3	371.9	3.4	18746.7	1.9

4-Cyclopentene-1,3-dione	4418.4	6.4	253.9	4.6	11639.3	11.8
2-Methyl-Benzofuran	3396.0	6.1	22.4	12.9	2444.4	7.3
1-(5-Methyl-2-Furanyl)-Ethanone	267.2	2.9	110.8	6.6	970.2	0.9
Diethylene Glycol Ethyl Ether	1027.6	14.2	24.3	7.5	2046.1	6.9
3-Methyl-Dihydro-2(3H)-Furanone	438.6	12.0	135.6	5.8	1905.2	2.6
2,3-Dihydro-1,1,4,6-Tetramethyl-1H-Indene	0.0		0.0		0.0	
Benzeneacetaldehyde	211.6	8.4	105.4	12.7	5725.5	2.6
3,5-Dimethyl-Dihydro-2-Furanone	467.1	15.0	15782.3	11.6	1210.6	2.1
2-Propenoic Acid	1392.2	4.8	876.8	5.8	7719.3	4.3
Ethyl Decanoate	0.0		0.0		2251.8	3.3
Acetophenone	0.0		0.0		0.0	
2-Furanmethanol	22314.2	1.7	26043.7	0.3	28262.0	4.2
3-Furanmethanol	36432.8	9.1	35767.9	7.5	29598.2	0.4
2,3-Dihydro-1,1,5,6-Tetramethyl-1H-Indene	0.0		6.5	15.1	298.0	0.3
2-(2-Furanylmethyl)-5-Methyl-Furan	0.0		14.6	4.1	20.1	6.7
3-Methyl-2(5H)-Furanone	341.1	4.9	107.5	6.2	729.4	2.6
5-Methyl-2-Furanmethanol	1525.8	4.2	156.8	1.4	1766.7	0.0
Benzyl Acetate	14.4	4.6	26.1	3.6	2030.0	2.1
2(5H)-Furanone	4672.0	11.5	8830.0	0.9	18121.2	1.3
2,2'-Methylenebis[5-Methyl-Furan	164.8	3.9	8.1	4.8	335.5	10.3
1,2-Cyclopentanedione	13747.0	11.4	636.5	8.3	23467.2	10.7
2-Cyclohexenol	2332.9	15.6	173.5	6.7	6820.4	7.8
2-Phenylethyl Acetate	728.0	3.7	12.1	1.3	8907.3	3.6
3-Methyl-1,2-Cyclopentanedione	3671.9	11.0	123.4	2.0	9352.2	6.2
2-Ethyl-Hexanoic Acid	0.0		0.0		0.0	
β-Damascenone	1788.6	4.8	420.2	6.1	335.3	6.1
Ethyl Undecanoate	0.0		0.0		4396.7	4.3
2-Methyl-Propanoic Acid	2987.5	8.4	200.4	8.4	7779.0	1.6
2,4,6-Trihydroxypyrimidine	115.1	8.1	110.2	4.6	183.7	13.9
Mequinol	568.3	4.2	87.9	6.4	1098.4	0.5
2,2-Diethyl-3-Methyl-Oxazolidine	1488.0	9.0	14.6	4.7	7218.6	1.3
Oxypurinol	2013.4	10.8	86.6	0.6	5598.4	9.5
Benzenemethanol	97.7	8.0	712.9	5.4	6035.1	5.0
4-Methyl-2(5H)-Furanone	345.8	6.1	180.4	4.6	1516.3	9.4

3-Ethyl-2-Hydroxy-2-Cyclopenten-1-one	57.2	8.1	147.9	2.0	58.7	9.5
Benzeneethanol	21.0	11.7	234.3	8.5	3935.6	1.3
Acrylamide	318.3	5.1	158.8	5.7	4892.5	1.6
Erythritol	4033.2	11.3	162.3	3.6	11590.7	2.5
2,3-Dimethyl-3-Pyrazolin-5-one	430.1	3.8	228.8	0.5	918.4	5.1
Furfural Acetone	275.0	3.3	25.7	8.8	680.4	3.0
Cyclotene	918.1	1.8	34.9	4.2	2451.7	0.7
2-Methyl-1,4-Benzenediol	607.3	4.7	51.6	4.8	2668.6	6.9
Heptanoic Acid	0.0		0.0		0.0	
Maltol	4193.9	6.7	452.2	0.4	18265.0	6.2
2-Acetylpyrrole	12048.1	11.9	162.2	1.0	6616.0	6.6
2,5-Furandicarboxaldehyde	2000.1	10.7	207.5	8.0	10560.1	8.4
2H-Pyran-2,6(3H)-Dione	3697.3	14.9	408.8	10.6	8833.4	0.6
1-(2-Furanyl)-2-Hydroxy-Ethanone	8757.6	12.3	322.7	16.5	22009.4	0.1
Phenol	1355.1	11.5	249.8	7.0	3044.0	7.3
1H-Pyrrole-2-Carboxaldehyde	465.0	5.0	271.3	2.9	764.6	1.5
Phloroglucinol	3482.9	5.4	197.4	6.4	11418.1	6.6
Furaneol	12225.3	5.2	210.8	10.9	17418.6	2.3
Octanoic Acid	0.0		0.0		0.0	
5-Acetyl-Dihydro-2(3H)-Furanone	10035.2	2.9	4429.2	10.3	13329.8	1.0
2-Furanpropionic Acid	1702.5	3.8	254.4	7.9	3792.1	9.9
1,3-Dihydroxy-2-Propanone	485174.0	3.9	388898.4	5.5	591962.9	2.8
Tetrahydro-5-Methyl-2-Furanmethanol	22011.4	0.6	23174.0	0.2	25808.6	6.7
4,5-Dimethyl Vinylene Carbonate	10237.0	4.3	849.0	9.7	20933.8	4.7
2-Hydroxy-Dihydro-2(3H)-Furanone	28684.2	1.6	1801.7	4.2	31450.3	15.9
5-Acetoxymethyl-2-Furfural A	3533.0	12.4	460.0	0.8	16472.2	3.0
5-Acetoxymethyl-2-Furfural B	6645.3	10.9	161.8	4.0	14550.9	9.3
2-Methoxy-4-Vinyl-Phenol	473.5	9.6	32.5	6.9	2040.3	8.3
3-Methyl-2,4(3H,5H)-Furandione	1946.3	0.4	108.9	8.7	4473.0	2.7
3-Hydroxy-2,3-Dihydro-Maltol	9598.6	4.6	379.0	9.4	17345.5	6.1
Decanoic Acid	1076.2	11.3	0.0		1723.3	9.2
5-Hydroxy-Maltol	10933.8	6.9	293.4	11.8	15669.2	2.4
2,3-Dihydro-Benzofuran	677.8	3.1	15.5	1.8	2176.2	6.0
1,4:3,6-Dianhydro-.alpha.-d-Glucopyranose	2210.5	12.9	224.5	15.1	5059.1	10.8

4-Pyridinol	209.9	5.6	167.5	7.9	764.5	2.0
4-Amino-Phenol	1906.5	8.1	329.2	2.5	5969.6	10.8
Benzoic Acid	412.8	2.9	83.8	1.8	4420.2	8.2
Dimethylhydrazone 3-Pentanone	7288.3	6.8	355.5	0.2	21524.1	7.6
Undecanoic Acid	0.0		750.8	2.7	0.0	
5-(Hydroxymethyl)-Dihydro-2(3H)-Furanone	27257.6	1.4	349.1	1.2	34394.5	7.4
Dodecanoic Acid	1030.8	8.8	0.0		1051.1	9.1
5-(Hydroxymethyl)-2-Furfural	140874.2	7.0	149808.3	4.0	132041.3	6.7
3-Methoxy-1,2-Benzenediol	111.4	4.7	167.7	5.6	586.3	0.2
1-(2-Furanyl)-1,2-Ethanediol	22317.1	10.3	28897.8	7.0	45811.6	10.0
4-Hydroxy-Dihydro-2(3H)-Furanone	7639.0	0.0	323.0	8.8	19828.1	4.9

[1]RSD - Relative Standard Deviation values.

Table S3C. Mean and relative standard deviation values of identified VOCs in sugarcane-based syrups samples from MED group.

Volatile Organic Compound	Relative Peak Areas (x 10^3)									
	MED									
	ESP16		EGP17		EGPA16		EGPB16		EGPC16	
	Mean	RSD (%)	Mean	RSD (%)	Mean	RSD (%)	Mean	RSD (%)	Mean	RSD (%)
Pentane	85.8	1.7	257.6	11.5	326.1	7.6	256.5	6.0	353.1	9.7
Hexane	293.5	9.6	410.6	5.5	470.4	2.7	63.2	0.8	161.9	4.5
1,4-Pentadiene	0.0		0.0		0.0		0.0		0.0	
Methanethiol	122.6	13.7	758.4	12.2	991.6	15.0	289.7	4.5	15.4	2.7
Ethanal	2100.1	4.5	6375.9	9.8	6589.4	14.4	2052.6	1.1	1209.7	2.8
Dimethyl Sulfide	31.0	2.5	14592.5	0.5	16372.3	4.1	13183.5	3.8	10944.4	3.1
Propanal	693.9	1.9	985.5	8.8	1310.8	4.2	345.4	8.6	263.6	2.3
Furan	892.0	1.6	2921.2	6.1	3175.7	2.4	1756.8	1.0	604.9	8.3
2-Methyl-Propanal	4577.4	4.4	8903.1	0.7	11437.7	3.8	8057.4	6.8	7306.5	3.0
Ethyl Formate	0.0		0.0		0.0		797.2	2.3	0.0	
2-Propenal	375.1	2.8	543.1	4.2	773.1	1.3	115.1	2.2	39.4	3.5
2-Methyl-Furan	1863.5	2.9	1991.1	5.5	5079.6	2.8	752.6	1.8	453.1	6.2
Ethyl Acetate	807.2	5.7	9272.9	4.4	368.7	3.6	643.5	5.4	78.1	9.7

2-Butanone	212.2	11.7	516.6	3.3	718.2	0.8	291.4	13.7	236.9	7.9
2-Methyl-Butanal	6177.6	8.6	11354.7	4.3	13906.9	3.0	11606.8	7.2	8844.6	3.3
3-Methyl-Butanal	3173.7	0.6	10858.7	4.0	27573.6	3.2	10669.6	9.7	4376.8	3.5
Ethanol	47770.4	5.9	104713.6	0.9	53733.8	5.3	37606.6	3.4	11995.2	1.6
Benzene	46.3	4.1	133.2	2.1	62.7	4.5	48.7	8.7	47.0	4.7
3-Buten-2-one	931.3	6.5	2533.4	5.6	1003.1	11.5	278.6	12.5	44.6	3.2
2,5-Dimethyl-Furan	829.2	12.9	1637.8	0.2	1041.0	1.0	826.6	7.7	144.5	5.6
Ethyl Propanoate	0.0		0.0		0.0		0.0		0.0	
2,3-Butanedione	8645.1	9.0	16239.7	10.6	15944.2	2.2	4665.0	10.4	1349.8	0.2
2-Methylpropyl Acetate	0.0		0.0		0.0		0.0		0.0	
1-Propanol	0.0		0.0		0.0		649.4	9.7	0.0	
2-Ethyl-5-Methyl-Furan	82.2	5.4	464.6	9.8	153.4	2.6	175.3	2.0	7.8	9.4
Toluene	19.7	0.2	1126.6	4.9	6926.8	1.1	1548.1	3.4	292.2	2.3
Ethyl Butanoate	0.0		144.3	0.7	7.5	10.4	0.0		0.0	
2-Methyl-3-Buten-2-ol	0.0		0.0		0.0		0.0		0.0	
Nonomethyl Succinate	0.0		3151.5	1.9	44.0	12.8	909.5	6.2	58.8	2.3
2,3-Pentanedione	3718.4	1.2	8015.3	3.5	7548.8	3.3	497.5	10.2	282.8	2.3
Dimethyl Disulfide	55.3	1.7	161.3	2.3	365.7	7.4	476.0	1.7	38.8	11.0
2-Ethenyl-Furan	57.5	5.1	454.0	2.3	629.7	2.4	104.6	8.7	34.8	6.1
2,5-Diethyltetrahydro-Furan	0.0		0.0		0.0		0.0		0.0	
Hexanal	0.0		417.7	0.1	612.9	10.7	27.7	8.2	50.2	12.7
2-Methyl-1-Propanol	31.0	12.8	140.1	6.4	41.2	6.9	71.1	4.0	14.2	3.0
5-Butyl-Dihydro-2(3H)-Furanone	88.1	3.3	95.3	4.3	98.1	5.0	7.0	6.5	10.8	2.3
m-Cresol	72.9	10.2	382.2	0.8	172.9	6.7	5.4	5.7	4.7	3.8
1-Butanol	18.1	4.9	0.0		0.0		0.0		18.0	5.7
5-Propyl-Dihydro-2(3H)-Furanone	11.2	6.2	131.2	1.4	671.5	3.0	15.8	2.1	10.4	1.6
3-Methyl-Pyridazine	66.2	7.7	535.5	1.8	1044.8	7.7	24.4	4.7	2.7	3.7
2-Heptanone	0.0		0.0		0.0		0.0		0.0	
m-Menthane	0.0		430.5	1.9	114.8	3.8	51.2	2.9	3.7	0.7
1,3-Diazine	20.3	0.8	1092.3	1.4	1773.1	2.2	30.1	16.3	11.4	3.5
2-Methyl-1-Butanol	539.1	2.1	0.0		0.0		4223.2	11.6	18.3	3.3
2-Propyl-Furan	15.2	4.3	72.3	1.2	296.4	3.1	31.2	10.1	8.1	5.8
Ethyl Hexanoate	0.0		0.0		0.0		0.0		0.0	
2-Pentyl-Furan	6.5	6.9	50.3	4.6	40.8	8.4	29.9	5.9	16.5	3.7

2-Methyl-Pyrazine	0.0		1562.7	2.6	1021.2	10.2	311.1	5.2	10.2	7.1
2-Methyl-Dihydro-2(3H)-Furanone	4119.1	5.3	2887.6	2.7	5045.5	9.2	3174.6	2.0	1562.8	4.8
p-Cymene	5.7	3.2	0.0		0.0		0.0		0.0	
Mesitylene	3.3	9.8	0.0		0.0		0.0		0.0	
3,5-Xylenol	10.7	2.7	0.0		0.0		0.0		0.0	
3-Hydroxy-2-Butanone	877.6	0.4	2151.9	1.3	1096.0	3.1	175.9	5.8	74.0	10.6
Vinylene Carbonate	5097.1	12.9	7713.3	5.4	7058.4	12.1	1616.0	1.5	895.7	13.9
2,6-Xylenol	257.1	7.3	15.5	2.5	163.2	3.9	14.6	0.8	4.7	12.9
1-Hydroxy-2-Propanone	1597.1	4.2	15435.8	1.9	11458.2	2.5	6837.4	1.8	2665.1	2.2
2,5-Dimethyl-Pyrazine	5.6	14.4	283.2	3.5	279.0	11.8	10.1	5.0	13.3	4.8
2,6-Dimethyl-Pyrazine	20.8	8.6	746.4	5.7	970.8	6.0	76.0	6.3	158.7	5.7
2-Cyclopenten-1-one	134.8	4.4	102.2	9.5	562.7	2.8	243.3	9.4	111.8	2.7
1,2,3,4-Tetramethyl-4-(1-methylethenyl)-Benzene	46.6	4.9	0.0		0.0		8.9	3.8	0.0	
2-Methyl-2-Cyclopenten-1-one	284.0	0.2	282.1	3.7	403.7	8.9	28.1	6.4	17.7	6.0
5-Methyl-2-Furanmethanethiol	230.6	3.8	2616.0	9.2	46.0	14.1	256.4	5.0	5.9	0.3
1-Hydroxy-2-Butanone	491.1	2.0	875.1	1.1	498.9	10.3	4493.7	2.7	1818.9	3.2
2-Ethyl-6-Methyl-Pyrazine	8.7	1.8	0.0		0.0		0.0		0.0	
2-Ethyl-5-Methyl-Pyrazine	0.0		0.0		0.0		0.0		0.0	
Nonanal	386.4	12.9	832.3	10.0	979.5	0.9	772.5	1.8	250.1	8.6
Trimethyl-Pyrazine	0.0		0.0		0.0		0.0		0.0	
3-Ethyl-2-Methyl-1,3-Hexadiene	0.0		0.0		0.0		21.4	11.3	0.0	
2-Cyclohexen-1-one	304.8	5.2	22.4	4.4	450.6	2.3	21.1	6.9	13.9	4.1
5-Methyl-2(3H)-Furanone	1383.9	5.9	2997.1	10.0	1720.9	2.4	1401.9	4.4	346.3	3.2
1,3-Dihydro-4-Methyl-2H-Imidazol-2-one	744.5	4.5	1906.7	6.2	1821.9	5.3	350.7	11.7	453.0	7.1
Ethyl Octanoate	0.0		0.0		0.0		0.0		0.0	
1,2,3,4-Tetrahydro-1,1,6-Trimethyl-Naphthalene	1344.5	1.4	1472.8	9.6	567.4	10.9	1486.2	2.9	9.5	8.2
Furfural	28145.9	9.6	34757.9	6.0	47378.6	8.3	36404.9	1.6	30413.7	2.1
Ethanoic Acid	204531.3	8.2	525881.1	3.0	663791.3	11.5	265425.9	9.8	199330.3	9.5
1,2,3,4-Tetrahydro-1,6,8-Trimethyl-Naphthalene	862.1	1.0	1009.5	0.8	353.3	15.7	806.7	0.8	40.2	8.0
2-Ethyl-1-Hexanol	451.8	3.3	786.3	5.0	327.4	6.2	300.8	1.9	297.1	1.6
3-Methyl-Furfural	127.8	0.7	175.5	0.7	161.1	3.6	56.2	12.2	29.0	10.8
Furfuryl Formate	352.6	2.4	1135.2	3.4	980.7	0.4	734.3	2.1	300.7	5.5
Decanal	169.1	2.1	105.9	5.7	72.1	2.5	94.9	8.4	48.1	4.6
1-(2-Furanyl)-Ethanone	4662.3	3.5	15360.0	2.6	12957.8	3.1	12403.3	3.1	5226.3	4.5

Benzaldehyde	435.4	2.4	355.7	13.9	288.3	16.1	90.9	6.9	64.0	8.8
Furfuryl Acetate	161.9	15.8	557.3	0.5	458.8	16.7	459.8	2.7	25.6	4.2
Propanoic Acid	1074.2	3.7	1837.2	1.0	1021.4	4.5	4673.1	4.4	4153.1	2.2
Dimethyl Sulfoxide	17.9	11.5	1654.7	10.8	1739.2	5.4	420.3	10.3	50.4	11.1
1,2,3,4-Tetrahydro-1,5,8-Trimethyl-Naphthalene	258.5	3.5	0.0		0.0		438.5	2.8	0.0	
5-Methyl-Furfural	6144.0	14.9	21002.4	3.5	16615.1	1.0	15673.5	2.9	10488.3	3.4
4-Cyclopentene-1,3-dione	4236.2	5.6	6329.9	7.0	2903.8	14.8	4060.6	9.0	4194.6	5.9
2-Methyl-Benzofuran	716.8	8.3	303.3	7.8	44.2	7.9	628.2	5.9	14.8	4.0
1-(5-Methyl-2-Furanyl)-Ethanone	388.0	5.3	1008.7	4.7	853.4	9.9	910.0	6.3	477.6	7.1
Diethylene Glycol Ethyl Ether	650.2	2.5	2312.5	12.3	3373.0	4.9	3420.0	6.9	1168.5	4.3
3-Methyl-Dihydro-2(3H)-Furanone	295.4	1.4	428.4	8.9	1144.2	10.5	393.5	13.1	1454.3	2.5
2,3-Dihydro-1,1,4,6-Tetramethyl-1H-Indene	175.8	5.7	0.0		0.0		132.6	1.7	6.2	12.6
Benzeneacetaldehyde	18.1	4.8	1667.0	6.8	2877.6	9.2	1090.0	9.9	239.3	14.5
3,5-Dimethyl-Dihydro-2-Furanone	359.6	6.4	940.5	10.2	936.1	10.1	890.8	1.7	901.1	3.3
2-Propenoic Acid	56.0	10.9	570.0	5.4	10367.4	10.4	331.0	9.1	1014.8	7.8
Ethyl Decanoate	0.0		0.0		0.0		0.0		0.0	
Acetophenone	0.0		0.0		0.0		0.0		0.0	
2-Furanmethanol	19541.0	1.2	30165.7	10.0	24400.6	3.4	24535.3	1.0	21311.4	5.7
3-Furanmethanol	15671.3	1.2	22768.3	0.5	25676.2	3.2	17544.2	1.5	11589.6	3.8
2,3-Dihydro-1,1,5,6-Tetramethyl-1H-Indene	479.3	7.2	1137.1	4.1	272.8	10.3	744.3	2.4	62.4	6.6
2-(2-Furanylmethyl)-5-Methyl-Furan	0.0		0.0		0.0		12.7	8.1	0.0	
3-Methyl-2(5H)-Furanone	103.2	5.2	836.5	5.2	848.2	1.0	192.6	6.6	147.3	2.7
5-Methyl-2-Furanmethanol	787.7	8.1	2193.5	7.6	0.0		727.6	8.9	404.8	2.6
Benzyl Acetate	0.0		0.0		0.0		0.0		0.0	
2(5H)-Furanone	4156.5	11.6	12488.8	5.9	7932.5	4.5	4913.4	2.2	3494.8	9.4
2,2'-Methylenebis[5-Methyl-Furan	49.5	0.1	124.6	6.5	85.3	3.1	86.4	14.5	6.6	10.5
1,2-Cyclopentanedione	18344.3	4.1	22941.3	13.5	24973.7	4.5	18434.2	1.8	16030.2	5.5
2-Cyclohexenol	704.8	0.6	5050.5	6.3	4231.7	6.7	3208.6	8.6	1076.5	3.3
2-Phenylethyl Acetate	109.0	5.1	12084.7	4.0	33.9	10.7	1504.0	1.4	6.1	3.6
3-Methyl-1,2-Cyclopentanedione	3391.4	2.1	7922.6	3.1	6026.4	3.7	3696.0	11.0	2952.7	9.4
2-Ethyl-Hexanoic Acid	0.0		1171.3	0.0	1670.2	12.7	2260.1	9.0	502.2	6.9
β-Damascenone	478.2	3.5	983.0	2.1	456.9	6.1	391.3	2.3	21.7	7.7
Ethyl Undecanoate	0.0		0.0		0.0		0.0		0.0	
2-Methyl-Propanoic Acid	676.0	1.1	5280.0	8.8	5265.2	8.9	1107.0	1.1	111.6	0.7

2,4,6-Trihydroxypyrimidine	164.2	10.9	377.1	8.3	396.7	4.1	111.6	9.5	91.4	1.7
Mequinol	744.8	3.0	1490.0	1.8	152.6	8.6	377.9	2.3	117.5	6.1
2,2-Diethyl-3-Methyl-Oxazolidine	908.3	3.2	6727.0	2.0	5941.0	4.4	317.1	6.0	1502.5	7.2
Oxypurinol	499.4	4.4	5320.4	10.7	4183.2	5.7	3530.7	9.5	2274.8	14.4
Benzenemethanol	21.9	13.8	84.0	2.2	97.4	10.4	43.1	9.6	67.9	7.0
4-Methyl-2(5H)-Furanone	428.4	10.0	968.8	6.4	1466.7	4.0	1025.2	3.4	529.3	14.3
3-Ethyl-2-Hydroxy-2-Cyclopenten-1-one	19.0	5.6	98.8	4.7	966.2	7.9	958.3	5.4	669.3	6.6
Benzeneethanol	867.2	1.4	1760.5	3.0	213.9	4.0	122.3	4.2	110.2	9.1
Acrylamide	22.0	6.8	4884.7	2.8	1039.9	2.1	163.4	1.4	496.7	7.9
Erythritol	1691.9	3.3	3848.6	3.7	7592.8	0.4	310.8	1.8	841.8	5.3
2,3-Dimethyl-3-Pyrazolin-5-one	0.0		605.2	6.9	596.9	6.1	195.9	4.5	23.9	12.9
Furfural Acetone	220.6	6.6	264.0	11.8	317.1	1.7	325.3	2.2	152.9	2.1
Cyclotene	368.6	0.7	2140.8	8.8	1375.6	6.0	222.2	5.1	206.9	6.4
2-Methyl-1,4-Benzenediol	201.9	5.8	912.0	6.3	1676.9	2.5	360.4	10.7	451.4	5.4
Heptanoic Acid	0.0		0.0		0.0		0.0		118.9	8.9
Maltol	2927.0	9.4	9132.6	1.6	9095.6	0.3	8504.1	11.0	5157.7	9.6
2-Acetylpyrrole	2100.4	5.8	3804.0	8.1	3154.5	11.5	2493.3	7.5	699.1	6.2
2,5-Furandicarboxaldehyde	2058.9	8.5	7971.6	1.2	10350.8	1.4	7332.6	5.8	3450.5	14.0
2H-Pyran-2,6(3H)-Dione	2561.5	7.3	7322.9	4.9	6084.3	5.7	4069.1	6.6	3737.3	3.7
1-(2-Furanyl)-2-Hydroxy-Ethanone	7511.3	9.9	28156.8	10.3	21112.8	3.2	8488.0	7.6	6400.2	2.0
Phenol	2100.0	7.3	4499.7	6.3	2457.0	8.1	3237.9	13.7	1658.8	11.1
1H-Pyrrole-2-Carboxaldehyde	369.0	9.2	1012.2	2.7	1899.6	3.1	429.6	11.1	275.4	2.3
Phloroglucinol	1682.2	5.4	7709.9	10.4	6758.4	11.5	1843.9	2.9	201.2	4.9
Furaneol	10011.8	6.9	14041.5	7.4	14676.6	1.4	10283.5	3.7	9742.6	8.3
Octanoic Acid	0.0		0.0		0.0		0.0		0.0	
5-Acetyl-Dihydro-2(3H)-Furanone	8964.3	6.8	12269.9	7.1	12879.9	13.8	7160.2	3.1	6083.8	9.7
2-Furanpropionic Acid	2146.9	9.7	4652.1	4.9	4047.3	3.9	2263.1	3.5	1877.3	6.6
1,3-Dihydroxy-2-Propanone	471324.1	1.0	585026.4	1.2	882133.4	9.8	516521.2	10.8	413356.1	5.7
Tetrahydro-5-Methyl-2-Furanmethanol	16541.6	1.9	24267.4	2.3	27213.4	7.3	22737.9	0.4	14263.5	3.6
4,5-Dimethyl Vinylene Carbonate	4719.2	0.1	17222.0	7.8	15798.9	1.7	1529.8	13.5	4095.9	2.3
2-Hydroxy-Dihydro-2(3H)-Furanone	29797.0	5.7	33121.2	4.6	28324.7	7.0	28439.7	4.8	20712.5	7.3
5-Acetoxymethyl-2-Furfural A	3560.9	7.8	14316.1	5.9	14914.9	4.2	7232.0	6.4	4774.4	6.4
5-Acetoxymethyl-2-Furfural B	5073.6	7.6	14739.2	2.5	15041.5	6.0	8248.8	5.0	5347.4	3.8
2-Methoxy-4-Vinyl-Phenol	455.4	4.9	1360.7	5.7	1262.1	6.7	590.6	0.9	169.7	7.0

3-Methyl-2,4(3H,5H)-Furandione	1624.7	1.5	2817.9	7.2	2723.2	0.8	1514.2	6.3	1344.5	2.0
3-Hydroxy-2,3-Dihydro-Maltol	8759.9	3.0	13181.7	14.5	5983.6	0.7	25737.7	2.8	19949.5	13.1
Decanoic Acid	607.5	3.8	1436.8	8.5	1303.6	7.0	0.0		0.0	
5-Hydroxy-Maltol	7607.8	11.9	14293.3	6.2	27699.0	8.1	9341.8	1.0	2268.1	10.2
2,3-Dihydro-Benzofuran	675.6	6.0	1714.6	9.2	1933.1	5.5	837.0	2.9	599.6	4.9
1,4:3,6-Dianhydro-.alpha.-d-Glucopyranose	3672.3	9.4	4346.6	8.3	6253.6	8.1	3581.6	4.0	1960.6	1.3
4-Pyridinol	419.3	6.9	926.0	3.6	662.8	12.6	388.8	2.9	143.7	4.0
4-Amino-Phenol	122.9	8.1	7602.0	7.3	6263.4	2.8	844.7	3.6	596.1	3.6
Benzoic Acid	435.2	1.1	1196.7	1.8	1773.2	3.9	128.6	5.4	253.7	12.6
Dimethylhydrazone 3-Pentanone	5390.8	4.9	18281.5	6.4	17348.6	9.5	4562.2	4.2	3431.6	11.6
Undecanoic Acid	0.0		2459.5	3.6	1318.0	4.7	230.9	2.3	220.9	11.6
5-(Hydroxymethyl)-Dihydro-2(3H)-Furanone	26678.3	13.4	33530.5	8.4	32799.5	9.7	24323.9	10.2	17277.8	5.3
Dodecanoic Acid	0.0		0.0		0.0		0.0		0.0	
5-(Hydroxymethyl)-2-Furfural	104330.5	3.7	178324.6	6.0	228281.1	2.1	188304.0	0.5	161590.9	5.2
3-Methoxy-1,2-Benzenediol	56.1	3.4	603.2	11.5	734.9	10.1	507.0	6.0	85.9	14.2
1-(2-Furanyl)-1,2-Ethanediol	21390.0	7.2	75845.7	14.2	67596.9	11.9	483.7	12.4	19537.8	6.0
4-Hydroxy-Dihydro-2(3H)-Furanone	30763.5	5.0	17737.7	9.7	15722.5	11.0	35188.3	8.4	4367.2	3.3

[1]RSD - Relative Standard Deviation values.

Table S3D. Mean and relative standard deviation values of identified VOCs in sugarcane-based syrups samples from STH group.

Volatile Organic Compound	Relative Peak Areas (x 10^3)					
	STH					
	MCBR14		MDBR14		AUS17	
	Mean	RSD (%)	Mean	RSD (%)	Mean	RSD (%)
Pentane	286.5	7.7	539.5	2.4	277.5	5.6
Hexane	47.5	11.2	281.7	0.7	312.7	12.4
1,4-Pentadiene	39.5	7.8	115.9	4.2	44.4	1.7
Methanethiol	124.4	5.7	691.5	1.8	361.8	5.7
Ethanal	1897.7	3.4	6446.1	0.8	4042.1	8.8
Dimethyl Sulfide	293.9	7.9	28601.0	2.1	11883.7	0.9
Propanal	420.1	2.9	2615.0	7.0	248.4	9.9

Furan	3045.0	9.5	2846.8	8.0	6643.8	12.7
2-Methyl-Propanal	7352.8	6.6	3278.2	7.0	8446.6	2.9
Ethyl Formate	1308.1	4.4	0.0		0.0	
2-Propenal	277.3	5.4	1413.5	1.6	406.4	5.2
2-Methyl-Furan	1572.6	2.9	5821.4	3.3	11228.2	2.9
Ethyl Acetate	731.4	2.8	54.9	6.2	92.5	6.1
2-Butanone	351.2	10.3	1282.1	1.4	961.2	7.2
2-Methyl-Butanal	9301.9	10.8	10566.0	0.3	11345.9	4.5
3-Methyl-Butanal	4848.3	6.5	6680.9	4.9	4338.9	4.8
Ethanol	37076.6	5.0	15898.0	4.1	66393.5	7.7
Benzene	51.8	5.1	379.6	1.8	32.9	8.4
3-Buten-2-one	893.9	9.3	4776.0	2.5	328.9	13.5
2,5-Dimethyl-Furan	2850.0	6.5	1794.1	4.7	3066.5	1.5
Ethyl Propanoate	0.0		0.0		0.0	
2,3-Butanedione	10220.2	6.1	31495.0	2.9	7161.8	8.3
2-Methylpropyl Acetate	0.0		0.0		0.0	
1-Propanol	0.0		0.0		0.0	
2-Ethyl-5-Methyl-Furan	418.7	3.7	413.2	4.6	363.3	13.5
Toluene	793.3	11.4	2311.5	7.3	8.2	2.8
Ethyl Butanoate	0.0		0.0		0.0	
2-Methyl-3-Buten-2-ol	427.9	8.4	1880.3	5.1	0.0	
Nonomethyl Succinate	846.3	5.4	0.0		0.0	
2,3-Pentanedione	3405.7	5.1	15461.8	4.9	1843.2	4.8
Dimethyl Disulfide	0.0		0.0		1860.0	7.7
2-Ethenyl-Furan	50.0	12.9	520.2	0.4	0.0	
2,5-Diethyltetrahydro-Furan	62.5	11.9	0.0		402.3	11.0
Hexanal	0.0		8030.0	3.4	0.0	
2-Methyl-1-Propanol	145.7	6.4	61.1	2.4	39.1	10.3
5-Butyl-Dihydro-2(3H)-Furanone	1125.9	4.4	22209.1	2.7	1823.3	4.7
m-Cresol	8.8	2.9	26.8	0.7	4.2	7.5
1-Butanol	0.0		0.0		0.0	
5-Propyl-Dihydro-2(3H)-Furanone	47.9	4.6	18187.8	7.3	424.2	12.4
3-Methyl-Pyridazine	132.8	5.4	549.0	3.2	317.4	7.7
2-Heptanone	0.0		0.0		0.0	

m-Menthane	0.0		17.7	6.4	0.0	
1,3-Diazine	3.4	4.3	78.9	4.1	283.9	8.7
2-Methyl-1-Butanol	74.0	0.6	299.4	1.6	224.1	3.7
2-Propyl-Furan	22.8	12.5	14.0	2.8	8.6	9.2
Ethyl Hexanoate	0.0		0.0		0.0	
2-Pentyl-Furan	17.5	4.2	618.1	4.6	19.2	5.4
2-Methyl-Pyrazine	0.0		18.6	2.0	2648.4	5.0
2-Methyl-Dihydro-2(3H)-Furanone	13256.6	8.7	4917.5	6.2	11510.2	7.0
p-Cymene	3.3	9.4	0.0		8.7	13.0
Mesitylene	5.4	12.1	0.0		4.8	10.1
3,5-Xylenol	172.6	4.7	0.0		0.0	
3-Hydroxy-2-Butanone	224.2	10.2	4437.5	9.2	264.5	7.0
Vinylene Carbonate	4188.6	3.3	11592.4	0.9	2263.5	6.5
2,6-Xylenol	753.1	11.6	22.1	4.6	4.2	4.3
1-Hydroxy-2-Propanone	27593.0	7.2	34460.5	8.8	2254.8	5.4
2,5-Dimethyl-Pyrazine	95.9	8.7	14.2	1.3	2183.0	6.1
2,6-Dimethyl-Pyrazine	96.5	3.6	108.8	7.3	2052.0	3.8
2-Cyclopenten-1-one	948.1	2.9	12436.6	5.8	286.3	13.2
1,2,3,4-Tetramethyl-4-(1-methylethenyl)-Benzene	0.0		981.3	0.1	0.0	
2-Methyl-2-Cyclopenten-1-one	136.4	6.6	1472.2	4.5	100.5	4.7
5-Methyl-2-Furanmethanethiol	691.8	8.1	21.5	2.2	6.1	10.5
1-Hydroxy-2-Butanone	4780.2	5.3	13379.9	6.4	407.8	8.7
2-Ethyl-6-Methyl-Pyrazine	5.0	4.1	0.0		965.4	2.1
2-Ethyl-5-Methyl-Pyrazine	0.0		0.0		411.2	4.6
Nonanal	719.0	3.9	465.4	2.4	57.0	6.2
Trimethyl-Pyrazine	0.0		0.0		349.3	3.5
3-Ethyl-2-Methyl-1,3-Hexadiene	0.0		878.3	1.6	0.0	
2-Cyclohexen-1-one	17.3	9.0	9266.3	1.0	6.3	8.6
5-Methyl-2(3H)-Furanone	1350.1	4.6	4675.6	2.9	1065.0	10.8
1,3-Dihydro-4-Methyl-2H-Imidazol-2-one	1133.2	10.6	5605.2	2.2	580.6	6.8
Ethyl Octanoate	0.0		0.0		0.0	
1,2,3,4-Tetrahydro-1,1,6-Trimethyl-Naphthalene	453.6	3.8	7118.8	4.0	65.6	6.8
Furfural	32710.8	10.8	29394.4	6.9	28442.4	9.5
Ethanoic Acid	331784.7	1.1	458847.3	0.7	258647.0	6.3

1,2,3,4-Tetrahydro-1,6,8-Trimethyl-Naphthalene	2291.4	9.3	5266.4	0.3	207.0	12.1
2-Ethyl-1-Hexanol	780.9	7.3	1378.2	5.4	528.5	8.4
3-Methyl-Furfural	132.4	12.0	351.1	1.2	42.3	1.2
Furfuryl Formate	656.1	8.4	657.3	1.5	253.3	7.8
Decanal	98.4	7.0	136.0	1.8	129.9	9.5
1-(2-Furanyl)-Ethanone	8719.1	11.3	13854.3	2.4	1157.4	7.3
Benzaldehyde	19.2	9.3	11608.6	3.7	34.5	10.5
Furfuryl Acetate	533.8	4.4	778.7	3.5	295.2	3.8
Propanoic Acid	4814.3	7.2	10580.2	2.3	53.1	1.9
Dimethyl Sulfoxide	77.5	9.1	1709.4	2.0	114.7	8.7
1,2,3,4-Tetrahydro-1,5,8-Trimethyl-Naphthalene	102.5	9.8	1546.0	1.4	0.0	
5-Methyl-Furfural	10730.2	10.0	7039.8	9.9	6260.5	7.0
4-Cyclopentene-1,3-dione	7077.1	5.2	13667.6	5.4	1381.5	3.4
2-Methyl-Benzofuran	187.9	4.4	610.9	0.3	89.4	9.3
1-(5-Methyl-2-Furanyl)-Ethanone	420.3	12.6	968.9	3.5	550.4	6.3
Diethylene Glycol Ethyl Ether	3573.1	10.0	264.1	9.2	410.6	10.7
3-Methyl-Dihydro-2(3H)-Furanone	1240.4	6.4	4245.1	1.1	470.8	7.9
2,3-Dihydro-1,1,4,6-Tetramethyl-1H-Indene	643.2	6.9	1438.7	6.0	27.4	5.2
Benzeneacetaldehyde	207.9	13.1	902.7	3.5	216.9	4.0
3,5-Dimethyl-Dihydro-2-Furanone	1106.4	11.2	3943.8	1.5	1103.7	7.6
2-Propenoic Acid	4715.6	9.6	8655.5	6.1	67.2	9.9
Ethyl Decanoate	0.0		0.0		0.0	
Acetophenone	0.0		516.3	1.2	0.0	
2-Furanmethanol	40480.6	8.3	20947.7	3.2	19671.2	3.9
3-Furanmethanol	41382.7	9.9	20633.1	0.6	12577.1	7.5
2,3-Dihydro-1,1,5,6-Tetramethyl-1H-Indene	2190.9	4.7	5077.2	2.6	145.0	7.1
2-(2-Furanylmethyl)-5-Methyl-Furan	8.5	5.4	4.2	5.1	0.0	
3-Methyl-2(5H)-Furanone	368.3	9.3	2167.0	2.2	155.9	5.2
5-Methyl-2-Furanmethanol	1066.8	6.5	3940.1	2.7	1573.3	9.8
Benzyl Acetate	4.4	3.5	0.0		0.0	
2(5H)-Furanone	7639.9	12.0	27146.8	2.2	2958.0	6.8
2,2'-Methylenebis[5-Methyl-Furan	353.2	6.8	42.5	4.1	26.8	11.3
1,2-Cyclopentanedione	32232.0	2.8	27157.7	8.4	11403.5	4.7
2-Cyclohexenol	3243.0	3.0	4990.1	2.9	2575.8	11.4

2-Phenylethyl Acetate	0.0		0.0		22.8	2.6
3-Methyl-1,2-Cyclopentanedione	5183.4	10.2	24172.1	4.0	3155.1	8.4
2-Ethyl-Hexanoic Acid	0.0		1114.6	0.2	0.0	
β-Damascenone	966.1	5.8	1997.2	2.0	1365.1	10.4
Ethyl Undecanoate	1151.6	2.2	0.0		0.0	
2-Methyl-Propanoic Acid	2824.1	12.4	26647.2	2.8	662.9	3.3
2,4,6-Trihydroxypyrimidine	153.7	3.1	789.7	2.0	208.9	6.9
Mequinol	132.2	9.0	274.1	1.7	1272.9	0.4
2,2-Diethyl-3-Methyl-Oxazolidine	2313.4	7.9	1732.9	1.3	81.6	10.7
Oxypurinol	3634.0	10.6	2952.8	9.9	791.4	6.9
Benzenemethanol	73.9	13.5	236.9	1.6	28.0	5.5
4-Methyl-2(5H)-Furanone	607.9	3.8	4930.2	6.6	1099.4	4.5
3-Ethyl-2-Hydroxy-2-Cyclopenten-1-one	692.8	4.9	6840.8	0.9	252.5	11.9
Benzeneethanol	602.9	3.0	315.8	0.6	66.0	9.0
Acrylamide	54.9	1.1	437.1	4.5	364.5	16.9
Erythritol	2087.8	3.0	34108.0	0.7	178.0	3.6
2,3-Dimethyl-3-Pyrazolin-5-one	0.0		1329.4	4.3	29.4	15.2
Furfural Acetone	278.8	11.4	670.7	1.3	186.9	4.4
Cyclotene	881.6	5.4	3771.7	8.6	40.5	3.6
2-Methyl-1,4-Benzenediol	738.3	8.8	576.5	5.2	305.6	8.3
Heptanoic Acid	0.0		1995.1	5.1	0.0	
Maltol	6016.3	3.9	4303.6	8.1	1559.3	1.7
2-Acetylpyrrole	2384.6	6.5	618.1	5.7	1289.9	8.3
2,5-Furandicarboxaldehyde	3215.6	11.4	2408.0	3.1	2015.9	7.2
2H-Pyran-2,6(3H)-Dione	7078.0	4.3	1870.8	5.3	66.9	8.7
1-(2-Furanyl)-2-Hydroxy-Ethanone	7788.8	8.1	10007.4	4.8	2681.3	9.6
Phenol	3347.2	12.6	9421.8	3.7	2690.1	6.8
1H-Pyrrole-2-Carboxaldehyde	169.3	11.4	8401.2	3.5	667.4	9.0
Phloroglucinol	1176.9	4.0	3703.4	11.3	136.6	10.1
Furaneol	13922.3	6.6	20922.6	4.9	7929.2	14.2
Octanoic Acid	0.0		5041.2	7.3	0.0	
5-Acetyl-Dihydro-2(3H)-Furanone	11221.8	12.1	17729.2	5.3	6473.0	12.1
2-Furanpropionic Acid	2306.8	11.6	1595.6	6.0	67.6	4.3
1,3-Dihydroxy-2-Propanone	534971.4	10.8	574093.3	1.0	432035.9	3.6

Tetrahydro-5-Methyl-2-Furanmethanol	24988.6	4.5	27991.3	8.5	20.1	13.9
4,5-Dimethyl Vinylene Carbonate	9102.2	10.4	14827.7	0.7	3690.0	8.3
2-Hydroxy-Dihydro-2(3H)-Furanone	35815.5	4.9	29518.2	2.7	32920.6	13.8
5-Acetoxymethyl-2-Furfural A	5644.7	2.3	3317.1	6.9	2500.9	5.5
5-Acetoxymethyl-2-Furfural B	6605.7	9.8	6362.9	0.6	4392.0	10.1
2-Methoxy-4-Vinyl-Phenol	1588.9	7.2	1317.5	3.6	1076.9	9.4
3-Methyl-2,4(3H,5H)-Furandione	2141.9	7.7	3997.5	1.3	1310.9	10.5
3-Hydroxy-2,3-Dihydro-Maltol	25425.9	2.3	30343.9	4.3	2150.2	12.4
Decanoic Acid	1520.2	7.9	17128.8	7.4	0.0	
5-Hydroxy-Maltol	9891.2	13.0	11660.8	1.4	103.5	5.8
2,3-Dihydro-Benzofuran	2327.1	15.7	1721.7	4.1	2326.0	1.5
1,4:3,6-Dianhydro-.alpha.-d-Glucopyranose	2470.2	13.4	9885.4	5.7	4319.2	14.4
4-Pyridinol	212.9	5.0	524.4	2.6	643.2	8.5
4-Amino-Phenol	1112.4	6.7	1073.4	2.3	1723.0	8.0
Benzoic Acid	207.5	2.2	49854.7	6.1	18.2	13.5
Dimethylhydrazone 3-Pentanone	9050.6	6.1	6763.8	3.2	2072.0	9.9
Undecanoic Acid	0.0		0.0		0.0	
5-(Hydroxymethyl)-Dihydro-2(3H)-Furanone	25179.9	4.4	27077.2	6.2	16016.6	3.0
Dodecanoic Acid	3057.4	6.1	1496.3	0.6	0.0	
5-(Hydroxymethyl)-2-Furfural	166665.3	5.9	63429.4	7.3	136780.2	4.7
3-Methoxy-1,2-Benzenediol	236.3	11.9	423.2	8.1	81.5	7.4
1-(2-Furanyl)-1,2-Ethanediol	42681.8	6.7	43532.9	4.9	23323.9	5.3
4-Hydroxy-Dihydro-2(3H)-Furanone	5502.0	12.2	12161.4	2.3	5550.6	5.3

[1]RSD - Relative Standard Deviation values.

Table S4. Mean, minimum and maximum peak area values of identified VOCs in sugarcane-based syrups samples.

Volatile Organic Compounds	Relative Peak Areas (x 10^3)											
	CERT			NCERT			MED			STH		
	Mean	Min	Max	Mean	Min	Max	Mean	Min	Max	Mean	Min	Max
Pentane	16.8	99.9	46.9	39.0	137.7	68.8	84.3	387.4	255.8	262.0	552.2	367.9
Hexane	60.4	265.0	130.5	69.3	242.9	139.2	62.8	483.0	279.9	42.2	351.4	214.0
1,4-Pentadiene	0.0	0.0	0.0	0.0	2.8	0.9	0.0	0.0	0.0	36.4	120.8	66.6
Methanethiol	118.2	1088.5	345.5	151.8	514.4	277.7	15.0	1139.9	435.5	117.3	703.6	392.5
Ethanal	1703.7	13999.5	7444.6	48.1	10363.4	5020.8	1175.8	7541.0	3665.5	1833.1	6500.3	4128.6
Dimethyl Sulfide	575.5	8188.6	2205.2	511.1	8414.8	3701.1	30.2	17048.5	11024.7	270.7	29191.9	13592.9
Propanal	261.2	1550.1	586.4	293.6	1513.2	854.6	257.6	1365.7	719.8	223.9	2797.8	1094.5
Furan	525.2	6043.5	2359.2	1964.8	3022.8	2292.8	554.7	3252.5	1870.1	2618.9	7488.0	4178.5
2-Methyl-Propanal	6228.8	38319.5	15491.3	6151.6	13601.3	9493.4	4375.9	11873.6	8056.4	3049.1	8691.3	6359.2
Ethyl Formate	0.0	7519.6	2265.5	0.0	3467.6	1770.7	0.0	815.9	159.4	0.0	1365.4	436.0
2-Propenal	145.2	578.2	323.7	26.0	561.6	299.1	38.0	783.2	369.2	262.2	1435.7	699.1
2-Methyl-Furan	624.9	7432.3	2883.0	165.7	2490.6	1591.6	425.0	5223.8	2028.0	1527.6	11554.7	6207.4
Ethyl Acetate	267.0	25835.5	8965.4	7.2	21438.9	10134.9	70.5	9679.4	2234.1	51.5	752.1	292.9
2-Butanone	189.2	4610.6	1104.4	64.1	594.2	332.8	187.3	724.3	395.1	315.0	1299.7	864.9
2-Methyl-Butanal	12490.3	41222.5	24426.1	8928.2	23464.0	15632.6	5645.1	14326.7	10378.1	8293.5	11858.9	10404.6
3-Methyl-Butanal	6399.9	47345.2	20221.3	1469.6	28625.9	12914.9	3153.2	28465.0	11330.5	4128.8	7007.2	5289.4
Ethanol	99041.5	217029.4	135755.4	2969.7	62772.1	36784.4	11808.6	105622.4	51163.9	15240.6	71477.3	39789.3
Benzene	17.1	209.1	98.9	3.4	123.5	52.7	44.4	136.1	67.6	30.1	386.5	154.8
3-Buten-2-one	263.5	5320.6	1872.2	41.3	3506.7	1527.3	43.2	2674.7	958.2	284.6	4895.1	1999.6
2,5-Dimethyl-Furan	482.8	4274.2	1575.0	83.4	2949.2	1360.3	136.4	1640.3	895.8	1709.4	3112.4	2570.2
Ethyl Propanoate	0.0	1964.7	472.3	0.0	10426.2	3442.4	0.0	0.0	0.0	0.0	0.0	0.0
2,3-Butanedione	1212.6	55294.5	19465.7	196.3	21154.3	11029.1	1347.7	17963.1	9368.8	6567.0	32420.5	16292.3
2-Methylpropyl Acetate	0.0	0.0	0.0	0.0	2128.9	681.8	0.0	0.0	0.0	0.0	0.0	0.0
1-Propanol	0.0	98.7	49.6	0.0	10496.4	3971.8	0.0	712.5	129.9	0.0	0.0	0.0
2-Ethyl-5-Methyl-Furan	24.6	595.7	234.2	0.0	746.9	239.6	7.0	510.3	176.7	314.3	434.1	398.4
Toluene	637.2	59213.8	12908.9	1145.8	5093.7	3410.6	19.6	6999.8	1982.7	8.0	2481.4	1037.7
Ethyl Butanoate	0.0	0.0	0.0	0.0	1353.1	423.6	0.0	145.3	30.4	0.0	0.0	0.0
2-Methyl-3-Buten-2-ol	0.0	3196.8	425.2	0.0	916.1	286.4	0.0	0.0	0.0	0.0	1975.6	769.4
Nonomethyl Succinate	0.0	3780.2	801.4	0.0	328.1	116.0	0.0	3212.1	832.8	0.0	891.8	282.1

2,3-Pentanedione	831.9	18346.9	5430.2	120.9	10026.0	4881.9	276.3	8295.7	4012.6	1754.0	16224.4	6903.6
Dimethyl Disulfide	0.0	325.6	137.1	0.0	2285.6	726.5	34.5	484.0	219.4	0.0	2002.5	620.0
2-Ethenyl-Furan	25.3	290.1	148.7	27.4	224.4	94.1	32.7	645.0	256.1	0.0	522.3	190.1
2,5-Diethyltetrahydro-Furan	0.0	1432.1	176.9	0.0	231.6	70.1	0.0	0.0	0.0	0.0	446.6	154.9
Hexanal	0.0	0.0	0.0	0.0	13.7	4.4	0.0	678.5	221.7	0.0	8303.8	2676.7
2-Methyl-1-Propanol	0.0	21211.9	3570.4	7.3	42253.3	13699.9	13.7	149.0	59.5	35.1	155.0	82.0
5-Butyl-Dihydro-2(3H)-Furanone	497.7	10094.7	2377.8	14.4	1199.5	376.1	6.5	103.0	59.9	1076.1	22802.6	8386.1
m-Cresol	147.6	3783.7	1268.6	18.7	1001.3	379.4	4.5	385.3	127.6	3.9	27.0	13.3
1-Butanol	16.8	1100.7	254.0	0.0	1090.0	375.9	0.0	19.0	7.2	0.0	0.0	0.0
5-Propyl-Dihydro-2(3H)-Furanone	270.6	2789.5	920.9	9.4	770.2	250.9	10.3	691.5	168.0	45.7	19515.9	6219.9
3-Methyl-Pyridazine	1524.7	9115.2	3702.9	11.5	2551.9	1087.4	2.6	1125.1	334.7	125.7	566.4	333.1
2-Heptanone	0.0	0.0	0.0	0.0	8.8	2.9	0.0	0.0	0.0	0.0	0.0	0.0
m-Menthane	3.1	155.7	38.1	0.0	0.0	0.0	0.0	438.5	120.0	0.0	18.8	5.9
1,3-Diazine	20.1	364.7	111.8	1.6	1036.8	451.5	11.0	1812.5	585.4	3.2	308.6	122.1
2-Methyl-1-Butanol	0.0	23747.0	8132.2	888.0	19408.9	8565.2	0.0	4714.5	956.1	73.6	304.0	199.2
2-Propyl-Furan	77.2	3680.4	1146.9	6.8	566.0	197.1	7.7	305.4	84.6	7.8	25.7	15.1
Ethyl Hexanoate	0.0	0.0	0.0	0.0	340.8	98.6	0.0	0.0	0.0	0.0	0.0	0.0
2-Pentyl-Furan	2.3	117.3	24.9	0.0	11.7	3.6	6.1	52.7	28.8	16.8	646.3	218.3
2-Methyl-Pyrazine	187.8	2296.5	1017.2	34.1	1954.3	888.0	0.0	1603.9	581.1	0.0	2781.5	889.0
2-Methyl-Dihydro-2(3H)-Furanone	10436.3	34517.2	19037.4	59.7	18762.0	6801.4	1487.6	5508.2	3357.9	4613.8	14410.4	9894.7
p-Cymene	19.5	120.9	56.5	0.0	230.8	88.8	0.0	5.9	1.1	0.0	9.8	4.0
Mesitylene	2.8	3835.9	499.7	0.0	120.6	40.2	0.0	3.6	0.7	0.0	6.0	3.4
3,5-Xylenol	43.4	396.3	167.4	0.0	89.6	29.5	0.0	11.0	2.1	0.0	180.8	57.5
3-Hydroxy-2-Butanone	1250.4	5427.4	2817.2	321.1	1961.9	1001.1	66.2	2179.5	875.1	201.4	4844.3	1642.0
Vinylene Carbonate	3994.6	27223.9	14822.8	79.6	13224.2	5634.9	771.3	8132.9	4476.1	2115.9	11693.9	6014.8
2,6-Xylenol	118.1	1679.0	660.4	4.6	289.2	153.6	4.1	275.8	91.0	4.0	840.5	259.8
1-Hydroxy-2-Propanone	5359.8	30542.4	14325.7	3669.8	21425.1	11092.4	1530.2	15728.7	7598.7	2133.8	37495.1	21436.1
2,5-Dimethyl-Pyrazine	36.4	1529.7	579.4	90.2	133.0	110.3	4.8	311.8	118.3	14.0	2316.6	764.4
2,6-Dimethyl-Pyrazine	1022.0	3449.8	1605.0	26.8	1510.4	822.4	19.0	1029.1	394.5	93.0	2130.2	752.4
2-Cyclopenten-1-one	1105.5	5960.4	2661.3	16.6	1134.8	494.5	92.6	578.5	231.0	248.5	13157.0	4557.0
1,2,3,4-Tetramethyl-4-(1-methylethenyl)-Benzene	0.0	0.0	0.0	0.0	0.0	0.0	0.0	48.9	11.1	0.0	981.9	327.1
2-Methyl-2-Cyclopenten-1-one	322.9	2259.1	1160.0	18.3	436.1	203.4	16.7	439.7	203.1	95.7	1538.7	569.7
5-Methyl-2-Furanmethanethiol	25.3	8606.1	2489.1	11.1	13804.7	5140.8	5.9	2855.6	631.0	5.4	747.5	239.8
1-Hydroxy-2-Butanone	625.6	10894.9	4798.4	98.9	4834.4	2054.1	447.4	4613.5	1635.5	372.5	14242.7	6189.3

2-Ethyl-6-Methyl-Pyrazine	115.0	432.8	183.7	13.8	158.0	63.3	0.0	8.8	1.7	0.0	985.6	323.4
2-Ethyl-5-Methyl-Pyrazine	0.0	0.0	0.0	0.0	0.0	0.0	0.0	0.0	0.0	0.0	430.0	137.1
Nonanal	471.5	2652.2	1048.9	187.1	2195.8	917.8	228.7	988.4	644.2	53.5	746.9	413.8
Trimethyl-Pyrazine	0.0	0.0	0.0	0.0	0.0	0.0	0.0	0.0	0.0	0.0	361.6	116.4
3-Ethyl-2-Methyl-1,3-Hexadiene	0.0	0.0	0.0	0.0	0.0	0.0	0.0	23.8	4.3	0.0	892.4	292.8
2-Cyclohexen-1-one	8.6	793.4	212.7	22.9	109.7	69.1	13.3	460.8	162.6	5.7	9355.0	3096.6
5-Methyl-2(3H)-Furanone	2863.0	13000.4	7643.6	156.9	4543.1	2074.9	335.2	3296.3	1570.0	950.3	4811.3	2363.6
1,3-Dihydro-4-Methyl-2H-Imidazol-2-one	1338.2	7356.9	3449.7	72.4	2446.3	1125.0	309.8	2025.7	1055.3	541.1	5730.1	2439.7
Ethyl Octanoate	0.0	0.0	0.0	0.0	1306.2	415.9	0.0	0.0	0.0	0.0	0.0	0.0
1,2,3,4-Tetrahydro-1,1,6-Trimethyl-Naphthalene	15.4	727.2	324.4	3.1	117.1	61.8	8.7	1613.5	976.1	61.2	7401.7	2546.0
Furfural	22947.6	41364.6	30033.0	22178.0	42904.6	30159.9	25433.9	51301.2	35420.2	25738.5	36254.7	30182.5
Ethanoic Acid	63131.0	636409.9	259844.9	214264.9	439351.3	341352.6	180455.2	740414.3	371792.0	242461.9	462065.1	349759.6
1,2,3,4-Tetrahydro-1,6,8-Trimethyl-Naphthalene	443.0	1463.8	738.5	4.8	370.0	192.5	37.0	1017.1	614.4	182.0	5283.0	2588.3
2-Ethyl-1-Hexanol	344.4	3128.3	1135.9	63.6	11756.1	4024.1	292.5	825.8	432.7	483.9	1452.0	895.9
3-Methyl-Furfural	167.8	909.7	437.5	218.8	4810.0	2234.7	25.9	176.8	109.9	41.8	355.2	175.3
Furfuryl Formate	756.1	4858.7	2427.0	54.6	1531.8	637.5	284.1	1173.4	700.7	233.6	711.3	522.2
Decanal	74.5	3543.2	1376.0	76.2	498.9	227.0	45.9	172.7	98.0	91.5	142.1	121.4
1-(2-Furanyl)-Ethanone	10211.4	17692.6	13246.7	40.4	16726.1	8782.4	4500.8	15752.8	10122.0	1073.1	14185.8	7910.3
Benzaldehyde	463.1	1921.5	1087.5	5.3	1255.3	548.5	58.3	446.0	246.8	17.4	12037.0	3887.4
Furfuryl Acetate	517.7	3495.2	1799.4	104.1	962.1	421.3	24.5	559.9	332.7	283.9	805.7	535.9
Propanoic Acid	207.0	7345.3	1616.1	0.0	7097.5	2632.4	975.2	4879.5	2551.8	52.0	10821.9	5149.2
Dimethyl Sulfoxide	43.0	768.2	381.9	44.4	350.8	151.0	15.8	1833.6	776.5	70.5	1743.1	633.9
1,2,3,4-Tetrahydro-1,5,8-Trimethyl-Naphthalene	113.2	633.5	318.6	5.0	305.1	135.3	0.0	450.8	139.4	0.0	1567.4	549.5
5-Methyl-Furfural	11661.3	24544.6	17921.8	359.4	19111.9	8116.8	5228.9	21728.0	13984.7	5824.5	11803.7	8010.2
4-Cyclopentene-1,3-dione	11200.1	23433.1	17369.9	242.1	13012.0	5437.2	2473.2	6775.3	4345.0	1334.4	14408.7	7375.4
2-Methyl-Benzofuran	2204.8	14920.2	7068.4	19.5	3604.7	1954.3	14.2	776.1	341.4	81.0	612.6	296.0
1-(5-Methyl-2-Furanyl)-Ethanone	499.1	4383.4	1901.4	103.5	978.6	449.4	367.3	1056.2	727.5	367.2	1003.3	646.5
Diethylene Glycol Ethyl Ether	1653.0	5846.8	3019.8	22.5	2187.8	1032.7	634.0	3656.2	2184.9	239.7	3930.2	1415.9
3-Methyl-Dihydro-2(3H)-Furanone	662.4	2081.1	1330.4	127.7	1955.1	826.5	291.4	1491.3	743.2	433.8	4291.6	1985.5
2,3-Dihydro-1,1,4,6-Tetramethyl-1H-Indene	29.4	136.5	68.8	0.0	0.0	0.0	0.0	185.8	62.9	25.9	1524.8	703.1
Benzeneacetaldehyde	726.6	19142.8	7726.2	92.0	5877.1	2014.1	17.3	3141.0	1178.4	180.7	934.2	442.5
3,5-Dimethyl-Dihydro-2-Furanone	425.6	3416.5	1287.3	396.9	17618.1	5820.0	336.7	1036.6	805.6	982.3	4002.7	2051.3

2-Propenoic Acid	40.1	13502.8	5851.7	825.5	8050.9	3329.4	49.9	11447.5	2467.8	60.5	9187.0	4479.4
Ethyl Decanoate	0.0	0.0	0.0	0.0	2326.1	750.6	0.0	0.0	0.0	0.0	0.0	0.0
Acetophenone	107.9	884.9	344.0	0.0	0.0	0.0	0.0	0.0	0.0	0.0	522.5	172.1
2-Furanmethanol	18118.3	56912.8	27308.2	21939.2	29450.6	25539.9	19309.6	33178.7	23990.8	18906.9	43825.7	27033.1
3-Furanmethanol	15255.1	68830.6	33416.9	29472.8	39746.2	33933.0	11151.0	26502.6	18649.9	11628.5	45494.0	24864.3
2,3-Dihydro-1,1,5,6-Tetramethyl-1H-Indene	82.7	570.6	306.8	0.0	298.8	101.5	58.2	1183.9	539.2	134.7	5207.1	2471.1
2-(2-Furanylmethyl)-5-Methyl-Furan	111.6	1122.5	531.9	0.0	21.5	11.6	0.0	13.7	2.5	0.0	9.0	4.2
3-Methyl-2(5H)-Furanone	246.7	1021.2	668.3	100.9	748.6	392.7	97.8	879.8	425.6	147.9	2215.4	897.1
5-Methyl-2-Furanmethanol	1243.5	6078.3	3056.2	154.7	1767.5	1149.8	0.0	2360.1	822.7	997.0	4046.3	2193.4
Benzyl Acetate	0.0	394.8	203.6	13.7	2072.9	690.1	0.0	0.0	0.0	0.0	4.6	1.5
2(5H)-Furanone	7621.7	38051.3	21720.5	4133.1	18361.9	10541.1	3165.6	13227.8	6597.2	2757.0	27733.0	12581.6
2,2'-Methylenebis[5-Methyl-Furan	292.6	1584.4	729.4	7.7	370.0	169.5	5.9	132.7	70.5	23.8	377.2	140.9
1,2-Cyclopentanedione	14388.7	42812.3	29007.4	583.7	25973.6	12616.9	15152.4	26088.5	20144.7	10871.3	33119.7	23597.7
2-Cyclohexenol	5070.0	18808.9	11409.9	161.8	7349.5	3108.9	700.4	5371.1	2854.4	2282.9	5136.6	3603.0
2-Phenylethyl Acetate	346.7	6315.9	2050.6	12.0	9227.2	3215.8	5.9	12566.9	2747.5	0.0	23.4	7.6
3-Methyl-1,2-Cyclopentanedione	6163.4	27187.7	14228.9	121.0	9928.3	4382.5	2675.4	8165.4	4797.8	2890.3	25139.6	10836.9
2-Ethyl-Hexanoic Acid	0.0	0.0	0.0	0.0	0.0	0.0	0.0	2464.6	1120.8	0.0	1117.2	371.5
β-Damascenone	195.2	11239.0	3725.2	314.9	1874.2	848.0	20.0	1003.5	466.2	910.5	2036.5	1442.8
Ethyl Undecanoate	0.0	0.0	0.0	0.0	4584.4	1465.6	0.0	0.0	0.0	0.0	1177.4	383.9
2-Methyl-Propanoic Acid	2174.0	13352.8	6316.8	183.5	7906.7	3655.6	110.8	5747.2	2487.9	641.2	27382.6	10044.7
2,4,6-Trihydroxypyrimidine	16209.4	34488.3	23333.6	105.2	209.3	136.4	89.8	413.0	228.2	149.0	805.3	384.1
Mequinol	539.1	3372.2	1440.6	82.3	1103.5	584.9	110.4	1517.0	576.6	120.3	1277.9	559.7
2,2-Diethyl-3-Methyl-Oxazolidine	1957.4	14005.0	6563.0	13.9	7309.8	2907.1	298.1	6861.3	3079.2	72.9	2497.2	1376.0
Oxypurinol	4387.7	15475.8	9210.9	86.1	6130.5	2566.2	477.5	5888.6	3161.7	736.5	4018.5	2459.4
Benzenemethanol	235.8	10917.3	2000.8	89.9	6338.7	2281.9	18.9	107.6	62.9	26.5	240.7	112.9
4-Methyl-2(5H)-Furanone	1403.4	7690.9	3718.5	172.2	1659.3	680.8	385.5	1525.8	883.7	584.6	5255.1	2212.5
3-Ethyl-2-Hydroxy-2-Cyclopenten-1-one	858.6	7308.4	2738.3	52.6	150.8	87.9	18.0	1042.9	542.3	222.5	6902.3	2595.4
Benzeneethanol	53.3	522.9	225.0	18.5	3986.0	1397.0	100.2	1812.8	614.8	60.1	621.0	328.2
Acrylamide	1148.6	7504.0	4439.0	149.8	4972.6	1789.9	20.5	5020.1	1321.4	54.3	456.9	285.5
Erythritol	3583.0	18880.0	9237.9	156.4	11885.3	5262.1	305.2	7624.6	2857.2	171.5	34330.0	12124.6
2,3-Dimethyl-3-Pyrazolin-5-one	501.9	2303.3	1283.1	227.6	965.4	525.8	0.0	646.9	284.4	0.0	1386.7	452.9
Furfural Acetone	455.0	2233.9	1191.5	23.4	700.5	327.0	149.7	332.3	256.0	178.6	679.4	378.8
Cyclotene	1418.7	8271.0	4290.3	33.5	2469.2	1134.9	193.6	2328.4	862.8	39.1	4095.2	1564.6

2-Methyl-1,4-Benzenediol	865.7	10124.3	3443.8	49.1	2852.0	1109.2	190.2	1719.6	720.5	280.2	803.1	540.1
Heptanoic Acid	0.0	0.0	0.0	0.0	0.0	0.0	0.0	129.5	23.8	0.0	2097.7	665.0
Maltol	7069.3	25568.3	15486.6	450.4	19398.6	7637.0	2651.9	9435.4	6963.4	1533.1	6249.6	3959.7
2-Acetylpyrrole	6981.8	31047.7	12772.3	160.6	13478.9	6275.4	655.7	4113.7	2450.3	582.9	2540.4	1430.9
2,5-Furandicarboxaldehyde	10985.7	48056.4	24639.8	191.0	11443.4	4255.9	1884.8	10496.9	6232.9	1871.2	3581.0	2546.5
2H-Pyran-2,6(3H)-Dione	1900.4	17495.8	10140.6	365.4	8890.3	4313.2	2375.4	7681.1	4755.0	61.0	7382.9	3005.2
1-(2-Furanyl)-2-Hydroxy-Ethanone	11782.3	57616.5	26518.6	269.4	22036.7	10363.2	6271.4	31068.2	14333.8	2423.6	10484.3	6825.8
Phenol	1793.7	12809.3	5289.8	232.3	3267.5	1549.6	1474.3	4781.3	2790.7	2507.7	9774.6	5153.0
1H-Pyrrole-2-Carboxaldehyde	599.2	6263.3	2929.6	263.4	776.2	500.3	269.1	1957.7	797.1	150.0	8692.2	3079.3
Phloroglucinol	2419.3	17924.6	9850.4	184.7	12170.1	5032.8	191.4	8508.4	3639.1	122.8	4123.1	1672.3
Furaneol	13072.7	19674.3	15660.9	188.0	17818.0	9951.6	8938.7	15074.7	11751.2	6801.4	21954.1	14258.0
Octanoic Acid	170.8	416.8	302.3	0.0	0.0	0.0	0.0	0.0	0.0	0.0	5411.1	1680.4
5-Acetyl-Dihydro-2(3H)-Furanone	10982.6	18325.1	13960.6	3975.2	13460.5	9264.7	5494.9	14655.9	9471.6	5688.6	18675.1	11808.0
2-Furanpropionic Acid	2201.0	11946.6	7426.0	234.3	4166.8	1916.4	1754.1	4878.2	2997.4	64.7	2575.1	1323.3
1,3-Dihydroxy-2-Propanone	277416.1	475218.7	374715.3	367532.2	608245.8	488678.4	389857.3	968707.3	573672.2	416352.8	592855.4	513700.2
Tetrahydro-5-Methyl-2-Furanmethanol	55.0	16816.7	7363.7	21888.5	27542.0	23664.7	13750.2	29202.3	21004.8	17.3	30368.1	17666.6
4,5-Dimethyl Vinylene Carbonate	5605.3	21117.7	11900.9	766.3	21915.2	10673.2	1323.4	18560.2	8673.2	3382.6	14934.6	9206.6
2-Hydroxy-Dihydro-2(3H)-Furanone	3724.0	36063.3	19282.0	1726.5	36461.2	20645.4	19202.1	34656.0	28079.0	28392.7	37588.3	32751.5
5-Acetoxymethyl-2-Furfural A	9370.2	18952.0	13291.1	456.3	16969.4	6821.7	3283.5	15535.7	8959.7	2363.1	5772.0	3820.9
5-Acetoxymethyl-2-Furfural B	12210.8	21938.5	16893.8	155.3	15901.9	7119.3	4686.6	15936.5	9690.1	3946.5	7251.3	5786.9
2-Methoxy-4-Vinyl-Phenol	731.7	6924.5	2914.0	30.2	2209.0	848.7	157.8	1438.1	767.7	975.7	1703.7	1327.8
3-Methyl-2,4(3H,5H)-Furandione	2926.3	11587.8	6993.8	99.4	4594.4	2176.1	1317.8	3020.6	2004.9	1173.6	4050.5	2483.4
3-Hydroxy-2,3-Dihydro-Maltol	6671.7	15163.6	10715.9	343.3	18409.8	9107.7	5941.1	26463.5	14722.5	1883.8	31652.9	19306.6
Decanoic Acid	191.4	3628.6	1223.7	0.0	1882.2	933.2	0.0	1559.2	669.6	0.0	18403.4	6216.3
5-Hydroxy-Maltol	9425.1	52877.1	27044.4	258.8	16051.4	8965.4	2035.9	29930.3	12242.0	97.4	11826.0	7218.5
2,3-Dihydro-Benzofuran	821.3	5941.1	2460.7	15.2	2306.8	956.5	570.5	2039.6	1152.0	1650.4	2693.5	2124.9
1,4:3,6-Dianhydro-.alpha.-d-Glucopyranose	2560.9	8896.0	5570.4	190.7	5604.9	2498.0	1934.8	6758.8	3962.9	2138.2	10448.9	5558.3
4-Pyridinol	3745.0	17161.6	9516.8	154.3	780.0	380.6	138.0	959.8	508.1	202.2	697.9	460.2
4-Amino-Phenol	1996.1	12202.3	6197.1	320.9	6613.5	2735.1	112.9	8155.0	3085.8	1038.1	1860.4	1302.9
Benzoic Acid	14.8	15964.1	3453.6	82.2	4784.0	1638.9	121.6	1842.8	757.5	15.7	52908.6	16693.5
Dimethylhydrazone 3-Pentanone	4837.5	22438.0	11102.9	354.9	23158.2	9722.6	3032.3	19449.6	9802.9	1866.9	9603.2	5962.1
Undecanoic Acid	72.0	11261.9	1971.3	0.0	771.5	250.3	0.0	2547.3	845.9	0.0	0.0	0.0
5-(Hydroxymethyl)-Dihydro-2(3H)-Furanone	19514.0	84401.1	37307.9	344.9	36947.0	20667.1	16365.9	36332.5	26922.0	15533.5	28762.7	22757.9

Dodecanoic Acid	68.7	3884.3	1750.3	0.0	1147.1	694.0	0.0	0.0	0.0	0.0	3244.1	1517.9
5-(Hydroxymethyl)-2-Furfural	151000.3	218661.0	177830.0	123179.0	155732.7	140907.9	100520.2	233092.4	172166.2	58828.0	176461.4	122291.6
3-Methoxy-1,2-Benzenediol	1870.1	15054.5	6072.2	106.1	587.4	288.5	54.2	809.2	397.4	75.5	457.4	247.0
1-(2-Furanyl)-1,2-Ethanediol	10279.8	43585.0	19107.1	20013.6	50379.3	32342.2	423.6	86629.7	36970.8	22088.8	45659.7	36512.9
4-Hydroxy-Dihydro-2(3H)-Furanone	6259.5	33221.5	18445.0	294.7	20803.5	9263.4	4221.8	38141.8	20755.9	4830.4	12436.1	7738.0

Table S5. VOCs identified in samples from this study and previously identified in others sugarcane-based syrups from other studies.

Volatile Organic Compounds	Sugarcane-Based Syrups						
	**Portugal (Madeira)[1]**	**Egypt[2]**	**USA[3]**	**Dominican Republic[4]**	**China[5]**	**Japan[6]**	**Japan[7]**
Pentane							
Hexane							
1,4-Pentadiene							
Methanethiol							
Ethanal	X						X
Dimethyl Sulfide	X	X					X
Propanal	X						X
Furan	X						
2-Methyl-Propanal	X	X					X
Ethyl Formate							
2-Propenal							
2-Methyl-Furan	X						
Ethyl Acetate	X						
2-Butanone	X	X					
2-Methyl-Butanal	X	X		X			X
3-Methyl-Butanal	X	X		X			X
Ethanol	X	X					X
Benzene							
3-Buten-2-one							
2,5-Dimethyl-Furan	X						
Ethyl Propanoate	X						
2,3-Butanedione	X			X			X

2-Methylpropyl Acetate						
1-Propanol	X					
2-Ethyl-5-Methyl-Furan	X					
Toluene						
Ethyl Butanoate						
2-Methyl-3-Buten-2-ol	X	X				
Nonomethyl Succinate						
2,3-Pentanedione	X					
Dimethyl Disulfide	X					
2-Ethenyl-Furan						
2,5-Diethyltetrahydro-Furan						
Hexanal	X				X	
2-Methyl-1-Propanol	X	X		X		
5-Butyl-Dihydro-2(3H)-Furanone	X					
m-Cresol				X		
1-Butanol	X					
5-Propyl-Dihydro-2(3H)-Furanone						
3-Methyl-Pyridazine						
2-Heptanone						
m-Menthane						
1,3-Diazine						
2-Methyl-1-Butanol	X	X		X		
2-Propyl-Furan						
Ethyl Hexanoate						
2-Pentyl-Furan						
2-Methyl-Pyrazine					X	
2-Methyl-Dihydro-2(3H)-Furanone	X					X
p-Cymene	X					
Mesitylene						
3,5-Xylenol						
3-Hydroxy-2-Butanone		X	X			X
Vinylene Carbonate						
2,6-Xylenol						
1-Hydroxy-2-Propanone	X	X				X

2,5-Dimethyl-Pyrazine			X		X		X
2,6-Dimethyl-Pyrazine	X				X		X
2-Cyclopenten-1-one							
1,2,3,4-Tetramethyl-4-(1-methylethenyl)-Benzene							
2-Methyl-2-Cyclopenten-1-one	X						
5-Methyl-2-Furanmethanethiol	X						
1-Hydroxy-2-Butanone							
2-Ethyl-6-Methyl-Pyrazine	X				X		
2-Ethyl-5-Methyl-Pyrazine							
Nonanal	X				X		
Trimethyl-Pyrazine							
3-Ethyl-2-Methyl-1,3-Hexadiene							
2-Cyclohexen-1-one							
5-Methyl-2(3H)-Furanone							
1,3-Dihydro-4-Methyl-2H-Imidazol-2-one							
Ethyl Octanoate							
1,2,3,4-Tetrahydro-1,1,6-Trimethyl-Naphthalene	X						
Furfural	X	X					
Ethanoic Acid	X	X	X	X			X
1,2,3,4-Tetrahydro-1,6,8-Trimethyl-Naphthalene							
2-Ethyl-1-Hexanol							
3-Methyl-Furfural							
Furfuryl Formate							
Decanal	X				X	X	
1-(2-Furanyl)-Ethanone	X					X	
Benzaldehyde	X						
Furfuryl Acetate	X						
Propanoic Acid		X					
Dimethyl Sulfoxide					X		
1,2,3,4-Tetrahydro-1,5,8-Trimethyl-Naphthalene	X						
5-Methyl-Furfural	X	X				X	
4-Cyclopentene-1,3-dione							
2-Methyl-Benzofuran	X						
1-(5-Methyl-2-Furanyl)-Ethanone						X	

Diethylene Glycol Ethyl Ether							
3-Methyl-Dihydro-2(3H)-Furanone							
2,3-Dihydro-1,1,4,6-Tetramethyl-1H-Indene							
Benzeneacetaldehyde	X						
3,5-Dimethyl-Dihydro-2-Furanone							
2-Propenoic Acid							
Ethyl Decanoate							
Acetophenone							
2-Furanmethanol	X	X	X		X	X	X
3-Furanmethanol							
2,3-Dihydro-1,1,5,6-Tetramethyl-1H-Indene							
2-(2-Furanylmethyl)-5-Methyl-Furan							
3-Methyl-2(5H)-Furanone							
5-Methyl-2-Furanmethanol	X				X		
Benzyl Acetate	X						
2(5H)-Furanone							
2,2'-Methylenebis[5-Methyl-Furan	X						
1,2-Cyclopentanedione							
2-Cyclohexenol							
2-Phenylethyl Acetate	X						
3-Methyl-1,2-Cyclopentanedione							
2-Ethyl-Hexanoic Acid							
β-Damascenone				X		X	
Ethyl Undecanoate							
2-Methyl-Propanoic Acid							
2,4,6-Trihydroxypyrimidine							
Mequinol	X			X		X	
2,2-Diethyl-3-Methyl-Oxazolidine							
Oxypurinol							
Benzenemethanol	X					X	
4-Methyl-2(5H)-Furanone							
3-Ethyl-2-Hydroxy-2-Cyclopenten-1-one							
Benzeneethanol	X			X		X	
Acrylamide			X				

Erythritol					
2,3-Dimethyl-3-Pyrazolin-5-one					
Furfural Acetone					
Cyclotene		X			X
2-Methyl-1,4-Benzenediol					
Heptanoic Acid					
Maltol					
2-Acetylpyrrole	X	X		X	X
2,5-Furandicarboxaldehyde					
2H-Pyran-2,6(3H)-Dione					
1-(2-Furanyl)-2-Hydroxy-Ethanone					
Phenol	X				
1H-Pyrrole-2-Carboxaldehyde					
Phloroglucinol					
Furaneol		X	X		
Octanoic Acid					
5-Acetyl-Dihydro-2(3H)-Furanone		X			
2-Furanpropionic Acid					
1,3-Dihydroxy-2-Propanone					
Tetrahydro-5-Methyl-2-Furanmethanol					
4,5-Dimethyl Vinylene Carbonate					
2-Hydroxy-Dihydro-2(3H)-Furanone					
5-Acetoxymethyl-2-Furfural A					
5-Acetoxymethyl-2-Furfural B					
2-Methoxy-4-Vinyl-Phenol		X			X
3-Methyl-2,4(3H,5H)-Furandione					
3-Hydroxy-2,3-Dihydro-Maltol					
Decanoic Acid			X		
5-Hydroxy-Maltol					
2,3-Dihydro-Benzofuran		X			
1,4:3,6-Dianhydro-.alpha.-d-Glucopyranose		X			
4-Pyridinol					
4-Amino-Phenol					
Benzoic Acid		X			X

Dimethylhydrazone 3-Pentanone				
Undecanoic Acid				
5-(Hydroxymethyl)-Dihydro-2(3H)-Furanone				
Dodecanoic Acid				
5-(Hydroxymethyl)-2-Furfural	X	X		X
3-Methoxy-1,2-Benzenediol				
1-(2-Furanyl)-1,2-Ethanediol				
4-Hydroxy-Dihydro-2(3H)-Furanone			X	

X – Identified in sugarcane based-product.

References

(1) Silva, P.; Freitas, J.; Silva, C. L.; Perestrelo, R.; Nunes, F. M.; Câmara, J. S. Establishment of Authenticity and Typicality of Sugarcane Honey Based on Volatile Profile and Multivariate Analysis. Food Control 2017, 73, 1176–1188. https://doi.org/10.1016/j.foodcont.2016.10.035.

(2) Edris, A. E.; Murkovic, M.; Siegmund, B. Application of Headspace-Solid-Phase Microextraction and HPLC for the Analysis of the Aroma Volatile Components of Treacle and Determination of Its Content of 5-Hydroxymethylfurfural (HMF). Food Chem. 2007, 104 (3), 1310–1314. https://doi.org/10.1016/j.foodchem.2006.10.033.

(3) Quinn, B. P.; Bernier, U. R.; Geden, C. J.; Hogsette, J. A.; Carlson, D. A. Analysis of Extracted and Volatile Components in Blackstrap Molasses Feed as Candidate House Fly Attractants. J. Chromatogr. A 2007, 1139 (2), 279–284. https://doi.org/10.1016/j.chroma.2006.11.039.

(4) Franitza, L.; Granvogl, M.; Schieberle, P. Influence of the Production Process on the Key Aroma Compounds of Rum: From Molasses to the Spirit. J. Agric. Food Chem. 2016, 64 (47), 9041–9053. https://doi.org/10.1021/acs.jafc.6b04046.

(5) Chen, E.; Song, H.; Li, Y.; Chen, H.; Wang, B.; Che, X.; Zhang, Y.; Zhao, S. Analysis of Aroma Components from Sugarcane to Non-Centrifugal Cane Sugar Using GC-O-MS. RSC Adv. 2020, 10 (54), 32276–32289. https://doi.org/10.1039/d0ra05963c.

(6) Abe, E.; Nakatani, Y.; Yamanishi, T.; Muraki, S. Studies on the" Sugary Flavor" of Raw Cane Sugar. I. Proc. Japan Acad. Ser. B Phys. Biol. Sci. 1978, 54 (9), 542–547. https://doi.org/10.2183/pjab.54.542.

(7) Asikin, Y.; Wada, K.; Imai, Y.; Kawamoto, Y.; Mizu, M.; Mutsuura, M.; Takahashi, M. Compositions, Taste Characteristics, Volatile Profiles, and Antioxidant Activities of Sweet Sorghum (Sorghum Bicolor L.) and Sugarcane (Saccharum Officinarum L.) Syrups. J. Food Meas. Charact. 2018, 12 (2), 884–891. https://doi.org/10.1007/s11694-017-9703-2.

Table S6A. Summary of number of volatile organic compounds identified, relative peak areas and total relative peak areas (%) values of main chemical classes identified in sugarcane-based syrups samples from CERT group.

Main Chemical Class	FRS07			FRS13			FRS14			FRS15			FRS16			FRS17			FRS18		
	N°[1]	RPA[2]	TRPA[3] (%)	N°[1]	RPA[2]	TRPA[3] (%)	N°[1]	RPA[2]	TRPA[3] (%)	N°[1]	RPA[2]	TRPA[3] (%)	N°[1]	RPA[2]	TRPA[3] (%)	N°[1]	RPA[2]	TRPA[3] (%)	N°[1]	RPA[2]	TRPA[3] (%)
Alcohol	9	238926	8.56	8	141489	9.48	8	117867	7.14	8	125395	9.33	8	177046	10.39	8	193986	9.25	5	195084	8.42
Aldehyde	8	110261	3.95	8	45983	3.08	8	36391	2.20	8	31888	2.37	8	57729	3.39	8	93030	4.44	8	121146	5.23
Benzene	13	64694	2.32	13	13223	0.89	13	10510	0.64	13	19716	1.47	13	49834	2.92	13	82987	3.96	12	39840	1.72
Benzofuran	2	16623	0.60	2	5103	0.34	2	3991	0.24	2	3832	0.29	2	9945	0.58	2	12791	0.61	2	14420	0.62
Carboxylic Acid	8	652840	23.38	8	321528	21.55	8	358502	21.71	8	80140	5.97	8	105433	6.19	8	211106	10.07	8	222590	9.61
Ester	6	59876	2.14	6	20255	1.36	6	22649	1.37	6	22873	1.70	6	57831	3.39	6	53139	2.54	3	37974	1.64
Ether	1	3325	0.12	1	1714	0.11	1	1815	0.11	1	2018	0.15	1	1869	0.11	1	5243	0.25	1	5155	0.22
Furan	45	711917	25.50	44	499327	33.47	44	551374	33.38	44	517432	38.52	44	624600	36.66	44	604150	28.82	44	750447	32.40
Hydrocarbon	2	358	0.01	2	134	0.01	2	83	0.01	2	97	0.01	2	195	0.01	2	147	0.01	2	228	0.01
Indene	2	323	0.01	2	136	0.01	2	286	0.02	2	319	0.02	2	353	0.02	2	600	0.03	2	612	0.03
Ketone	16	627082	22.46	16	354013	23.73	16	430465	26.06	16	399621	29.75	16	432674	25.39	16	587460	28.02	16	642068	27.72
Naphthalene	3	1290	0.05	3	1038	0.07	3	1334	0.08	3	810	0.06	3	1571	0.09	3	2531	0.12	3	1096	0.05
Nitrogen	16	136025	4.87	16	47471	3.18	16	59396	3.60	16	62281	4.64	16	86140	5.06	16	123170	5.88	16	128124	5.53
Phenol	8	45120	1.62	8	8409	0.56	8	12521	0.76	8	15127	1.13	8	29237	1.72	8	34839	1.66	8	49265	2.13
Pyran	5	104079	3.73	5	30917	2.07	5	42422	2.57	5	56803	4.23	5	65921	3.87	5	87172	4.16	5	95391	4.12
Sulfur	3	9225	0.33	4	864	0.06	4	1815	0.11	4	1585	0.12	4	2305	0.14	4	3538	0.17	3	2155	0.09
Terpene	3	10290	0.37	3	393	0.03	3	268	0.02	3	3357	0.25	3	1264	0.07	3	341	0.02	3	10826	0.47

[1]Number of volatile organic compounds identified.

[2]Relative Peak Areas values.

[3]Total Relative Peak Areas percentage values.

Table S6B. Summary of number of volatile organic compounds identified, relative peak areas and total relative peak areas (%) values of main chemical classes identified in sugarcane-based syrups samples from NCERT group.

Main Chemical Class	ECAL14			ENCAL14			GLA14		
	Nº[1]	RPA[2]	TRPA[3] (%)	Nº[1]	RPA[2]	TRPA[3] (%)	Nº[1]	RPA[2]	TRPA[3] (%)
Alcohol	9	74710	4.68	7	48908	4.68	8	104618	4.82
Aldehyde	8	72891	4.56	9	17437	1.67	8	45767	2.11
Benzene	12	6628	0.41	11	2663	0.25	12	40772	1.88
Benzofuran	2	4074	0.25	2	38	0.00	2	4621	0.21
Carboxylic Acid	6	359032	22.47	4	237798	22.74	6	461712	21.26
Ester	6	28397	1.78	4	965	0.09	11	77463	3.57
Ether	1	1028	0.06	1	24	0.00	1	2046	0.09
Furan	43	436967	27.34	43	330082	31.56	43	583090	26.85
Hydrocarbon	2	155	0.01	3	114	0.01	2	358	0.02
Indene	0	0	0.00	1	7	0.00	1	298	0.01
Ketone	17	537614	33.64	16	394890	37.76	16	702576	32.35
Naphthalene	3	428	0.03	3	14	0.00	3	727	0.03
Nitrogen	16	29900	1.87	16	1823	0.17	16	56333	2.59
Phenol	7	8104	0.51	7	921	0.09	8	24915	1.15
Pyran	5	30634	1.92	5	1758	0.17	5	65172	3.00
Sulfur	4	5666	0.35	3	7870	0.75	3	1033	0.05
Terpene	2	1843	0.12	1	420	0.04	2	548	0.03

[1]Number of volatile organic compounds identified.

[2]Relative Peak Areas values.

[3]Total Relative Peak Areas percentage values.

Table S6C. Summary of number of volatile organic compounds identified, relative peak areas and total relative peak areas (%) values of main chemical classes identified in sugarcane-based syrups samples from MED group.

Main Chemical Class	ESP16			EGP17			EGPA16			EGPB16			EGPC16		
	Nº[1]	RPA[2]	TRPA[3] (%)	Nº[1]	RPA[2]	TRPA[3] (%)	Nº[1]	RPA[2]	TRPA[3] (%)	Nº[1]	RPA[2]	TRPA[3] (%)	Nº[1]	RPA[2]	TRPA[3] (%)
Alcohol	7	51207	4.22	5	114539	5.23	5	65927	2.53	7	46370	3.09	7	14261	1.27
Aldehyde	8	17653	1.46	9	40377	1.84	9	63256	2.43	9	33742	2.25	9	22389	2.00
Benzene	12	2261	0.19	10	19924	0.91	10	14686	0.56	11	5452	0.36	10	1618	0.14
Benzofuran	2	1392	0.11	2	2018	0.09	2	1977	0.08	2	1465	0.10	2	614	0.05
Carboxylic Acid	5	206945	17.06	7	538636	24.58	7	684737	26.26	6	274028	18.28	7	205452	18.37
Ester	3	10623	0.88	5	37504	1.71	5	23277	0.89	5	5496	0.37	4	5128	0.46
Ether	1	650	0.05	1	2313	0.11	1	3373	0.13	1	3420	0.23	1	1169	0.10
Furan	43	361628	29.81	43	623287	28.44	42	655896	25.15	44	477822	31.87	43	366930	32.80
Hydrocarbon	2	379	0.03	2	668	0.03	2	797	0.03	2	320	0.02	2	515	0.05
Indene	2	655	0.05	1	1137	0.05	1	273	0.01	2	877	0.06	2	69	0.01
Ketone	16	514880	42.44	16	670634	30.60	16	958064	36.74	16	561424	37.45	16	444025	39.69
Naphthalene	3	2465	0.20	2	2482	0.11	2	921	0.04	3	2731	0.18	2	50	0.00
Nitrogen	16	10739	0.89	15	48065	2.19	15	42134	1.62	15	12995	0.87	15	9588	0.86
Phenol	8	5446	0.45	7	23060	1.05	7	17230	0.66	7	6915	0.46	7	2753	0.25
Pyran	5	25529	2.10	5	48277	2.20	5	55116	2.11	5	51234	3.42	5	33073	2.96
Sulfur	4	227	0.02	4	17167	0.78	4	19469	0.75	4	14369	0.96	4	11049	0.99
Terpene	2	484	0.04	2	1414	0.06	2	572	0.02	3	464	0.03	2	25	0.00

[1]Number of volatile organic compounds identified.

[2]Relative Peak Areas values.

[3]Total Relative Peak Areas percentage values.

Table S6D. Summary of number of volatile organic compounds identified, relative peak areas and total relative peak areas (%) values of main chemical classes identified in sugarcane-based syrups samples from STH group.

Main Chemical Class	MCBR14			MDBR14			AUS17		
	Nº[1]	RPA[2]	TRPA[3] (%)	Nº[1]	RPA[2]	TRPA[3] (%)	Nº[1]	RPA[2]	TRPA[3] (%)
Alcohol	7	43836	2.60	7	58615	2.74	6	69939	5.67
Aldehyde	8	24916	1.48	9	39631	1.85	8	29015	2.35
Benzene	11	2941	0.17	11	68107	3.19	11	819	0.07
Benzofuran	2	2515	0.15	2	2333	0.11	2	2415	0.20
Carboxylic Acid	6	348716	20.68	9	531506	24.86	4	259430	21.04
Ester	6	17328	1.03	3	26475	1.24	3	6046	0.49
Ether	1	3573	0.21	1	264	0.01	1	411	0.03
Furan	45	526628	31.23	44	473805	22.16	43	355368	28.82
Hydrocarbon	3	374	0.02	3	937	0.04	3	635	0.05
Indene	2	2834	0.17	2	6516	0.30	2	172	0.01
Ketone	16	629608	37.34	16	778171	36.40	16	461884	37.46
Naphthalene	3	2848	0.17	3	13931	0.65	2	273	0.02
Nitrogen	15	19440	1.15	15	29924	1.40	18	15939	1.29
Phenol	8	8292	0.49	7	15839	0.74	7	6908	0.56
Pyran	5	50882	3.02	5	58064	2.72	5	8199	0.66
Sulfur	3	496	0.03	3	31002	1.45	4	14220	1.15
Terpene	2	969	0.06	4	2893	0.14	2	1374	0.11

[1]Number of volatile organic compounds identified.

[2]Relative Peak Areas values.

[3]Total Relative Peak Areas percentage values.

Table S7. One-way ANOVA test results based on the relative peak areas of the identified VOCs in sugarcane-based syrups samples.

Volatile Organic Compounds	One-way ANOVA Test					
	All Samples		Tukey Test[3] ($\alpha < 0.05$)			
	F[1]	P[2]	CERT (A)	NCERT (B)	MED (C)	STH (D)
Pentane	48.69	7.22E-15	C; D	C; D	A; B; D	A; B; C
Hexane	6.19	1.17E-03	C	C	A; B	
1,4-Pentadiene	49.98	4.44E-15	D	D	D	A; B; C
Methanethiol	0.55	6.48E-01				
Ethanal	3.90	1.39E-02	C		A	
Dimethyl Sulfide	10.62	1.63E-05	C; D	C; D	A; B	A; B
Propanal	1.66	1.87E-01				
Furan	4.36	8.35E-03	D		D	A; C
2-Methyl-Propanal	6.29	1.05E-03	C; D		A	A
Ethyl Formate	6.50	8.41E-04	C; D		A	A
2-Propenal	4.14	1.07E-02	D	D	D	A; B; C
2-Methyl-Furan	7.02	4.96E-04	D	D	D	A; B; C
Ethyl Acetate	6.88	5.71E-04	C; D	C; D	A; B	A; B
2-Butanone	2.67	5.76E-02				
2-Methyl-Butanal	17.62	6.14E-08	B; C; D	A	A	A
3-Methyl-Butanal	4.84	4.90E-03	D			A
Ethanol	38.57	4.72E-13	B; C; D	A	A	A
Benzene	2.93	4.25E-02				
3-Buten-2-one	1.00	4.00E-01				
2,5-Dimethyl-Furan	6.32	1.02E-03	D	D	D	A; B; C
Ethyl Ester Propanoic Acid	6.06	1.34E-03	B	A; C; D	B	B
2,3-Butanedione	1.97	1.30E-01				
2-Methylpropyl Acetate	7.54	2.96E-04	B	A; C; D	B	B
1-Propanol	10.48	1.85E-05	B	A; C; D	B	B
2-Ethyl-5-Methyl-Furan	2.34	8.43E-02				
Toluene	3.73	1.69E-02	C		A	
Ethyl Butanoate	6.54	8.12E-04	B	A; C; D	B	B
2-Methyl-3-Buten-2-ol	1.98	1.29E-01				

Nonomethyl Succinate	1.48	2.32E-01				
2,3-Pentanedione	0.67	5.73E-01				
Dimethyl Disulfide	3.01	3.86E-02				
2-Ethenyl-Furan	1.81	1.57E-01				
2,5-Diethyltetrahydro-Furan	1.17	3.32E-01				
Hexanal	6.57	7.86E-04	D	D	D	A; B; C
2-Methyl-1-Propanol	4.77	5.31E-03	B	A; C; D	B	B
5-Butyl-Dihydro-2(3H)-Furanone	7.17	4.29E-04	D	D	D	A; B; C
m-Cresol	7.92	2.04E-04	B; C; D	A	A	A
1-Butanol	4.26	9.35E-03		C	B	
5-Propyl-Dihydro-2(3H)-Furanone	6.25	1.10E-03	D	D	D	A; B; C
3-Methyl-Pyridazine	17.95	4.85E-08	B; C; D	A	A	A
2-Heptanone	6.94	5.37E-04	B	A; C; D	B	B
m-Menthane	4.49	7.22E-03		C	B; D	C
1,3-Diazine	4.21	9.93E-03	C		A	
2-Methyl-1-Butanol	7.80	2.29E-04	C; D	C; D	A; B	A; B
2-Propyl-Furan	8.62	1.03E-04	B; C; D	A	A	A
Ethyl Hexanoate	6.79	6.29E-04	B	A; C; D	B	B
2-Pentyl-Furan	6.50	8.47E-04	D	D	D	A; B; C
2-Methyl-Pyrazine	0.91	4.45E-01				
2-Methyl-Dihydro-2(3H)-Furanone	23.37	1.35E-09	B; C; D	A	A	A
p-Cymene	11.15	1.02E-05	C; D	C; D	A; B	A; B
Mesitylene	1.67	1.85E-01				
3,5-Xylenol	15.25	3.56E-07	B; C; D	A	A	A
3-Hydroxy-2-Butanone	7.23	4.00E-04	B; C	A	A	
Vinylene Carbonate	11.87	5.49E-06	B; C; D	A	A	A
2,6-Xylenol	7.97	1.93E-04	B; C	A	A	
1-Hydroxy-2-Propanone	4.87	4.76E-03			D	C
2,5-Dimethyl-Pyrazine	3.76	1.64E-02				
2,6-Dimethyl-Pyrazine	9.80	3.41E-05	B; C; D	A	A	A
2-Cyclopenten-1-one	6.76	6.49E-04	C	D	A, D	B; C
1,2,3,4-Tetramethyl-4-(1-methylethenyl)-Benzene	6.78	6.33E-04	D	D	D	A; B; C
2-Methyl-2-Cyclopenten-1-one	12.45	3.36E-06	B; C; D	A	A	A
5-Methyl-2-Furanmethanethiol	5.25	3.17E-03		C; D	B	B

1-Hydroxy-2-Butanone	4.92	4.51E-03	C		A; D	C
2-Ethyl-6-Methyl-Pyrazine	5.56	2.25E-03	A	D	A; D	B; C
2-Ethyl-5-Methyl-Pyrazine	6.93	5.44E-04	D	D	D	A; B; C
Nonanal	3.02	3.83E-02	D			A
Trimethyl-Pyrazine	6.94	5.41E-04	D	D	D	A; B; C
3-Ethyl-2-Methyl-1,3-Hexadiene	6.87	5.77E-04	D	D	D	A; B; C
2-Cyclohexen-1-one	6.19	1.16E-03	D	D	D	A; B; C
5-Methyl-2(3H)-Furanone	29.65	3.71E-11	B; C; D	A	A	A
1,3-Dihydro-4-Methyl-2H-Imidazol-2-one	8.43	1.24E-04	B; C	A	A	
Ethyl Octanoate	6.93	5.44E-04	B	A; C; D	B	B
1,2,3,4-Tetrahydro-1,1,6-Trimethyl-Naphthalene	6.21	1.14E-03	D	D		A; B
Furfural	2.57	6.44E-02				
Ethanoic Acid	1.59	2.03E-01				
1,2,3,4-Tetrahydro-1,6,8-Trimethyl-Naphthalene	12.54	3.13E-06	D	D	D	A; B; C
2-Ethyl-1-Hexanol	4.98	4.23E-03	B	A; C; D	B	B
3-Methyl-Furfural	16.94	1.01E-07	B	A; C; D	B	B
Furfuryl Formate	17.23	8.15E-08	B; C; D	A	A	A
Decanal	14.47	6.54E-07	B; C; D	A	A	A
1-(2-Furanyl)-Ethanone	3.94	1.33E-02	D			A
Benzaldehyde	4.98	4.21E-03	D	D	D	A; B; C
Furfuryl Acetate	20.88	6.61E-09	B; C; D	A	A	A
Propanoic Acid	3.32	2.70E-02	D			A
Dimethyl Sulfoxide	3.01	3.87E-02		C	B	
1,2,3,4-Tetrahydro-1,5,8-Trimethyl-Naphthalene	3.47	2.28E-02			D	C
5-Methyl-Furfural	12.90	2.31E-06	B; D	A; C	B; D	A; C
4-Cyclopentene-1,3-dione	42.70	7.89E-14	B; C; D	A	A	A
2-Methyl-Benzofuran	27.39	1.27E-10	B; C; D	A	A	A
1-(5-Methyl-2-Furanyl)-Ethanone	11.80	5.84E-06	B; C; D	A	A	A
Diethylene Glycol Ethyl Ether	5.67	2.00E-03	B; D	A		A
3-Methyl-Dihydro-2(3H)-Furanone	4.62	6.25E-03		D	D	B; C
2,3-Dihydro-1,1,4,6-Tetramethyl-1H-Indene	17.05	9.30E-08	D	D	D	A; B; C
Benzeneacetaldehyde	11.51	7.47E-06	B; C; D	A	A	A
3,5-Dimethyl-Dihydro-2-Furanone	5.54	2.31E-03	B	A; C	B	
2-Propenoic Acid	2.09	1.14E-01				

Ethyl Decanoate	6.94	5.40E-04	B	A; C; D	B	B
Acetophenone	13.09	1.98E-06	B; C	A	A	
2-Furanmethanol	0.50	6.84E-01				
3-Furanmethanol	5.07	3.82E-03	C	C	A; B	
2,3-Dihydro-1,1,5,6-Tetramethyl-1H-Indene	14.77	5.16E-07	D	D	D	A; B; C
2-(2-Furanylmethyl)-5-Methyl-Furan	28.08	8.70E-11	B; C; D	A	A	A
3-Methyl-2(5H)-Furanone	2.66	5.80E-02				
5-Methyl-2-Furanmethanol	13.31	1.65E-06	B; C	A	A; D	C
Benzyl Acetate	6.18	1.18E-03	B	A; C; D	B	B
2(5H)-Furanone	10.38	2.02E-05	B; C; D	A	A	A
2,2'-Methylenebis[5-Methyl-Furan	24.37	7.39E-10	B; C; D	A	A	A
1,2-Cyclopentanedione	9.93	3.05E-05	B; C	A; D	A	B
2-Cyclohexenol	23.74	1.08E-09	B; C; D	A	A	A
2-Phenylethyl Acetate	1.70	1.78E-01				
3-Methyl-1,2-Cyclopentanedione	8.89	7.98E-05	B; C	A	A	
2-Ethyl-Hexanoic Acid	16.91	1.03E-07	C	C	A; B; D	C
β-Damascenone	4.62	6.25E-03	C		A	
Ethyl Undecanoate	6.25	1.09E-03	B	A; C	B	
2-Methyl-Propanoic Acid	3.65	1.86E-02			D	C
2,4,6-Trihydroxypyrimidine	180.09	≤1.00E-19	B; C; D	A	A	A
Mequinol	7.19	4.20E-04	B; C; D	A	A	A
2,2-Diethyl-3-Methyl-Oxazolidine	7.29	3.79E-04	B; C; D	A	A	A
Oxypurinol	23.80	1.04E-09	B; C; D	A	A	A
Benzenemethanol	3.17	3.23E-02				
4-Methyl-2(5H)-Furanone	13.71	1.19E-06	B; C	A	A	
3-Ethyl-2-Hydroxy-2-Cyclopenten-1-one	8.08	1.74E-04	B; C	A; D	A; D	B; C
Benzeneethanol	4.25	9.45E-03	B	A; D		C
Acrylamide	13.60	1.30E-06	B; C; D	A	A	A
Erythritol	3.43	2.40E-02			D	C
2,3-Dimethyl-3-Pyrazolin-5-one	13.49	1.43E-06	B; C; D	A	A	A
Furfural Acetone	22.71	2.03E-09	B; C; D	A	A	A
Cyclotene	17.59	6.30E-08	B; C; D	A	A	A
2-Methyl-1,4-Benzenediol	7.42	3.32E-04	B; C; D	A	A	A
Heptanoic Acid	6.74	6.57E-04	D	D	D	A; B; C

Maltol	15.39	3.21E-07	B; C; D	A	A	A
2-Acetylpyrrole	17.38	7.30E-08	B; C; D	A	A	A
2,5-Furandicarboxaldehyde	30.06	2.98E-11	B; C; D	A	A	A
2H-Pyran-2,6(3H)-Dione	9.16	6.17E-05	B; C; D	A	A	A
1-(2-Furanyl)-2-Hydroxy-Ethanone	9.33	5.29E-05	B; C; D	A	A	A
Phenol	6.26	1.08E-03	B; C	A; D	A	B
1H-Pyrrole-2-Carboxaldehyde	4.50	7.14E-03	B; C	A	A	
Phloroglucinol	10.07	2.69E-05	B; C; D	A	A	A
Furaneol	5.05	3.91E-03	B; C	A	A	
Octanoic Acid	6.11	1.26E-03	D	D	D	A; B; C
5-Acetyl-Dihydro-2(3H)-Furanone	6.80	6.22E-04	B; C	A	A	
2-Furanpropionic Acid	19.07	2.23E-08	B; C; D	A	A	A
1,3-Dihydroxy-2-Propanone	10.30	2.18E-05	C; D		A	A
Tetrahydro-5-Methyl-2-Furanmethanol	15.38	3.23E-07	B; C; D	A	A	A
4,5-Dimethyl Vinylene Carbonate	0.89	4.55E-01				
2-Hydroxy-Dihydro-2(3H)-Furanone	4.53	6.94E-03	D			A
5-Acetoxymethyl-2-Furfural A	11.55	7.21E-06	B; C; D	A	A; D	A; C
5-Acetoxymethyl-2-Furfural B	22.74	2.00E-09	B; C; D	A	A	A
2-Methoxy-4-Vinyl-Phenol	10.71	1.51E-05	B; C; D	A	A	A
3-Methyl-2,4(3H,5H)-Furandione	22.67	2.09E-09	B; C; D	A	A	A
3-Hydroxy-2,3-Dihydro-Maltol	4.00	1.25E-02	D	D		A; B
Decanoic Acid	6.02	1.39E-03	D	D	D	A; B; C
5-Hydroxy-Maltol	13.13	1.92E-06	B; C; D	A	A	A
2,3-Dihydro-Benzofuran	6.15	1.22E-03	B; C	A	A	
1,4:3,6-Dianhydro-.alpha.-d-Glucopyranose	4.60	6.39E-03	B	A; D		B
4-Pyridinol	45.77	2.25E-14	B; C; D	A	A	A
4-Amino-Phenol	7.14	4.38E-04	B; C; D	A	A	A
Benzoic Acid	4.98	4.22E-03	D	D	D	A; B; C
Dimethylhydrazone 3-Pentanone	1.27	2.96E-01				
Undecanoic Acid	2.03	1.21E-01				
5-(Hydroxymethyl)-Dihydro-2(3H)-Furanone	4.65	6.04E-03	B; D	A		A
Dodecanoic Acid	8.96	7.45E-05	C		A; D	C
5-(Hydroxymethyl)-2-Furfural	8.22	1.51E-04	B; D	A	D	A; C
3-Methoxy-1,2-Benzenediol	22.36	2.55E-09	B; C; D	A	A	A

1-(2-Furanyl)-1,2-Ethanediol	3.47	2.28E-02	C		A	
4-Hydroxy-Dihydro-2(3H)-Furanone	6.27	1.07E-03	D	C	B; D	A; C

[1]F Value - Fischer value from One-way ANOVA test.
[2]P Value - Probability value from One-way ANOVA test.
[3]Samples with significantly different relative peak area values determined by one-way ANOVA with Post-hoc Tukey test ($\alpha < 0.05$).

Table S8. Information summary of Principal Component Analysis (PCA) and Partial Least Square Analysis (PLS) based on the relative peak areas of the identified VOCs in sugarcane-based syrups samples.

Analysis	Component	R^2X	Eigenvalues	R^2Y	Q^2	Significance	Iterations
PCA	1	0.3542	57.37	n.a.	0.322	S	6
	2	0.1846	29.90	n.a.	0.020	S	13
	3	0.1105	17.90	n.a.	0.109	S	11
	4	0.0533	8.64	n.a.	0.019	S	8
	5	0.0485	7.86	n.a.	0.055	S	12
	6	0.0374	6.06	n.a.	-0.002	UNKNOWN	50
PLS	1	0.3856	57.07	0.058	0.014	S	10
	2	0.1982	29.34	0.059	0.001	S	9
	3	0.1158	17.14	0.059	0.019	S	11
	4	0.0562	8.31	0.059	0.006	S	8
	5	0.0479	7.09	0.059	0.012	S	12
	6	0.0361	5.35	0.059	0.011	S	25
	7	0.0307	4.55	0.058	0.020	S	33
	8	0.0290	4.29	0.059	0.023	S	13
	9	0.0211	3.12	0.059	0.019	S	24
	10	0.0206	3.05	0.059	0.041	S	15
	11	0.0147	2.17	0.058	0.026	S	50
	12	0.0138	2.04	0.059	0.086	S	13
	13	0.0091	1.35	0.058	0.084	S	16
	14	0.0072	1.07	0.058	0.117	S	10
	15	0.0043	0.64	0.058	0.068	S	21

16	0.0030	0.45	0.058	0.117	S	3
17	0.0026	0.39	0.058	0.900	S	3
18	0.0007	0.09	0.002	0.094	S	10

n.a. - not available.

Table S9. Loading results and variable importance in projection scores of variables from Principal Component Analysis (PCA) and Partial Least Squares Analysis (PLS) based on the relative peak areas of the identified VOCs in samples sugarcane-based syrups samples.

Volatile Organic Compounds	Abbreviations	PCA					PLS				
		1	2	3	VIP[1] Importance	VIP[1] Power (x 100)	1	2	3	VIP[1] Importance	VIP[1] Power (x 100)
Pentane	PTANE	-0.254	-0.731	0.050	112	78.55	-0.03	-0.14	0.01	21	10.79
Hexane	HXANE	0.026	-0.253	0.145	110	80.04	0.00	-0.05	0.03	12	11.76
1,4-Pentadiene	PT14DIENE	-0.065	-0.904	-0.026	50	94.19	-0.01	-0.17	-0.01	98	6.05
Ethanal	ETAL	0.900	0.001	0.232	34	95.60	0.12	0.00	0.06	116	5.40
Dimethyl Sulfide	DMSULFI	-0.158	-0.763	-0.106	106	82.37	-0.02	-0.14	-0.03	32	9.61
Furan	FUR	0.244	-0.062	-0.130	134	55.20	0.03	-0.01	-0.03	15	11.50
2-Methyl-Propanal	MPPAL	0.782	0.281	-0.213	72	90.37	0.10	0.05	-0.05	61	7.73
Ethyl Formate	EESTFA	0.363	0.280	0.237	136	51.32	0.05	0.05	0.06	17	11.06
2-Propenal	PPENAL	0.163	-0.790	0.136	108	80.59	0.02	-0.15	0.03	30	9.70
2-Methyl-Furan	M2FUR	0.314	-0.248	-0.175	49	94.21	0.04	-0.05	-0.04	27	9.83
Ethyl Acetate	EESTAA	0.380	0.307	0.586	124	67.97	0.05	0.05	0.14	37	9.51
2-Methyl-Butanal	M2BTAL	0.857	0.252	-0.197	94	85.46	0.11	0.05	-0.04	43	9.03
3-Methyl-Butanal	M3BTAL	0.729	0.191	-0.194	126	66.86	0.10	0.03	-0.04	41	9.20
Ethanol	ETOL	0.742	0.340	-0.435	91	85.94	0.10	0.06	-0.10	53	8.35
Benzene	BNZ	0.490	-0.787	-0.096	84	87.55	0.06	-0.15	-0.02	56	8.13
2,5-Dimethyl-Furan	DM25FUR	0.268	-0.024	0.379	100	83.90	0.04	-0.01	0.09	19	10.87
Ethyl Propanoate	EESTPA	0.142	0.153	0.947	2	99.00	0.02	0.02	0.23	97	6.21
2-Methylpropyl Acetate	MP2ESTAA	0.064	0.120	0.934	30	96.00	0.01	0.02	0.23	90	6.60
1-Propanol	P1POL	0.037	0.135	0.922	48	94.25	0.00	0.02	0.23	77	6.91
Toluene	TOLNE	0.416	0.134	0.042	140	40.09	0.06	0.02	0.01	9	12.22
Ethyl Butanoate	EESTBA	0.062	0.117	0.943	24	96.27	0.01	0.02	0.23	85	6.62

Dimethyl Disulfide	DMDSFD	-0.274	0.141	-0.175	137	49.62	-0.04	0.03	-0.04	14	11.58
Hexanal	HXAL	0.060	-0.985	0.045	10	97.94	0.01	-0.18	0.01	129	4.97
2-Methyl-1-Propanol	M2PP1OL	0.167	0.183	0.927	21	96.57	0.02	0.03	0.22	83	6.65
5-Butyl-Dihydro-2(3H)-Furanone	BDH2FURONE	0.295	-0.897	-0.063	29	96.03	0.04	-0.17	-0.02	109	5.61
m-Cresol	MCREOL	0.882	0.200	-0.079	101	83.58	0.12	0.04	-0.02	92	6.57
1-Butanol	BT1OL	0.371	0.236	0.734	87	87.20	0.05	0.04	0.18	57	8.10
5-Propyl-Dihydro-2(3H)-Furanone	PDH2FURONE	0.173	-0.961	0.004	14	97.51	0.02	-0.18	0.00	127	5.02
3-Methyl-Pyridazine	M3PYRDZNE	0.881	0.234	-0.052	95	85.43	0.12	0.04	-0.01	96	6.26
2-Heptanone	HPT2ONE	-0.151	0.087	-0.064	144	6.95	-0.02	0.02	-0.01	8	12.53
m-Menthane	MMTHANE	0.170	-0.002	-0.031	130	62.40	0.02	0.00	-0.01	3	14.24
1,3-Diazine	D13ZINE	0.001	0.067	0.438	97	84.45	0.00	0.01	0.10	22	9.98
2-Methyl-1-Butanol	M2BT1OL	0.315	0.298	0.609	105	82.49	0.04	0.05	0.15	50	8.66
2-Propyl-Furan	PP2FUR	0.751	0.225	-0.068	129	64.35	0.10	0.04	-0.01	28	9.81
Ethyl Hexanoate	EESTHA	0.068	0.116	0.928	47	94.60	0.01	0.02	0.23	89	6.60
2-Pentyl-Furan	PT2FUR	0.067	-0.973	0.018	31	95.81	0.01	-0.18	0.00	70	7.19
2-Methyl-Dihydro-2(3H)-Furanone	MDH2FURONE	0.710	0.227	-0.345	116	75.37	0.09	0.04	-0.08	45	8.96
p-Cymene	PCYMNE	0.346	0.306	0.769	73	89.84	0.05	0.05	0.19	75	7.00
3,5-Xylenol	XYL35NOL	0.816	0.247	-0.072	104	82.90	0.11	0.04	-0.02	86	6.61
3-Hydroxy-2-Butanone	HXY3BT2ONE	0.902	-0.336	-0.034	45	94.70	0.12	-0.06	-0.01	113	5.48
Vinylene Carbonate	VYLESTCA	0.977	-0.013	0.064	23	96.47	0.13	0.00	0.02	112	5.49
2,6-Xylenol	XYL26NOL	0.671	0.230	-0.104	122	71.31	0.09	0.04	-0.02	47	8.85
1-Hydroxy-2-Propanone	HXY1PP2ONE	0.574	-0.524	0.263	120	72.83	0.08	-0.10	0.06	46	8.91
2,5-Dimethyl-Pyrazine	DM25PYZNE	0.111	0.206	-0.193	117	74.78	0.02	0.04	-0.05	2	16.06
2,6-Dimethyl-Pyrazine	DM26PYZNE	0.571	0.316	-0.157	114	76.08	0.08	0.06	-0.04	49	8.77
2-Cyclopenten-1-one	CY2PT1EONE	0.514	-0.819	-0.055	3	98.59	0.07	-0.15	-0.01	134	4.86
1,2,3,4-Tetramethyl-4-(1-methylethenyl)-Benzene	TM4MEYL1BNZ	0.059	-0.981	0.039	5	98.56	0.01	-0.18	0.01	131	4.96
2-Methyl-2-Cyclopenten-1-one	M2CY2PT1EONE	0.935	-0.232	-0.143	18	96.85	0.12	-0.04	-0.03	110	5.59
5-Methyl-2-Furanmethanethiol	M2FURTHOL	0.209	0.248	0.879	56	93.22	0.03	0.04	0.21	67	7.50
1-Hydroxy-2-Butanone	H1BT2ONE	0.658	-0.604	0.008	99	84.04	0.09	-0.11	0.00	76	7.00
2-Ethyl-6-Methyl-Pyrazine	E6MPYZNE	0.096	0.172	-0.278	65	91.81	0.01	0.03	-0.07	38	9.33
2-Ethyl-5-Methyl-Pyrazine	E5MPYZNE	-0.230	0.045	-0.171	52	94.12	-0.03	0.01	-0.04	35	9.57
Nonanal	NONAL	0.582	0.167	-0.206	139	44.99	0.08	0.03	-0.05	29	9.71
Trimethyl-Pyrazine	TMPYZNE	-0.230	0.045	-0.172	51	94.12	-0.03	0.01	-0.04	34	9.57
3-Ethyl-2-Methyl-1,3-Hexadiene	E3M2HX13DENE	0.067	-0.981	0.041	4	98.58	0.01	-0.18	0.01	132	4.94

2-Cyclohexen-1-one	C2HEXONE	0.102	-0.971	0.049	12	97.80	0.01	-0.18	0.01	124	5.12
5-Methyl-2(3H)-Furanone	M5FURONE	0.952	0.054	-0.039	58	92.91	0.13	0.01	-0.01	108	5.63
1,3-Dihydro-4-Methyl-2H-Imidazol-2-one	M4IMDZONE	0.900	-0.358	0.011	37	95.41	0.12	-0.07	0.00	136	4.81
Ethyl Octanoate	EESTOA	0.068	0.117	0.936	28	96.05	0.01	0.02	0.23	88	6.60
1,2,3,4-Tetrahydro-1,1,6-Trimethyl-Naphthalene	T116TNP	0.035	-0.962	0.029	44	94.72	0.00	-0.18	0.00	80	6.82
1,2,3,4-Tetrahydro-1,6,8-Trimethyl-Naphthalene	T168TNP	0.163	-0.917	0.018	71	90.84	0.02	-0.17	0.00	66	7.50
2-Ethyl-1-Hexanol	E2HX1OL	0.223	0.074	0.929	35	95.44	0.03	0.01	0.23	84	6.63
3-Methyl-Furfural	M3FURAL	-0.081	0.113	-0.134	143	15.04	-0.01	0.02	-0.02	11	11.78
Furfuryl Formate	FURYLFMTE	0.831	0.231	0.045	53	93.86	0.11	0.04	0.01	72	7.04
Decanal	DECAL	0.877	0.212	-0.176	89	86.20	0.12	0.04	-0.04	33	9.61
1-(2-Furanyl)-Ethanone	FURYLONE	0.740	-0.115	0.344	93	85.50	0.10	-0.02	0.08	58	8.10
Benzaldehyde	BENZAL	0.265	-0.936	0.070	11	97.90	0.03	-0.17	0.02	122	5.22
Furfuryl Acetate	FURYLACTE	0.948	0.135	-0.076	39	95.15	0.13	0.02	-0.02	125	5.10
Propanoic Acid	PPANOIC	0.030	-0.633	0.543	111	79.82	0.00	-0.12	0.13	26	9.90
Dimethyl Sulfoxide	DMSULFO	0.202	-0.569	0.091	90	86.14	0.03	-0.11	0.02	42	9.07
1,2,3,4-Tetrahydro-1,5,8-Trimethyl-Naphthalene	T158TNP	0.327	-0.817	0.113	81	88.32	0.04	-0.15	0.03	52	8.43
5-Methyl-Furfural	M5FURAL	0.734	0.262	0.209	92	85.63	0.10	0.05	0.05	60	7.93
4-Cyclopentene-1,3-dione	CPT4E13DONE	0.909	-0.019	0.018	67	91.48	0.12	0.00	0.00	102	5.91
2-Methyl-Benzofuran	M2BNZFUR	0.885	0.253	-0.139	64	91.89	0.12	0.05	-0.03	91	6.57
1-(5-Methyl-2-Furanyl)-Ethanone	M5FURYLONE	0.923	0.088	-0.107	78	88.73	0.12	0.01	-0.02	101	5.95
Diethylene Glycol Ethyl Ether	DENGOLEETHR	0.669	0.275	-0.068	119	73.05	0.09	0.05	-0.02	44	9.01
3-Methyl-Dihydro-2(3H)-Furanone	M3DHFURONE	0.513	-0.737	0.244	76	89.11	0.07	-0.14	0.06	40	9.22
2,3-Dihydro-1,1,4,6-Tetramethyl-1H-Indene	DHT1146MIDNE	0.047	-0.926	0.004	70	90.95	0.01	-0.17	0.00	79	6.83
Benzeneacetaldehyde	BENZACETAL	0.926	0.198	-0.013	55	93.26	0.12	0.04	0.00	104	5.80
3,5-Dimethyl-Dihydro-2-Furanone	DM35DHFURONE	-0.182	-0.107	-0.131	142	23.36	-0.02	-0.02	-0.03	4	14.09
Ethyl Ester Decanoic Acid	EESTDA	0.068	0.117	0.936	26	96.10	0.01	0.02	0.23	87	6.61
Acetophenone	ACETPHONE	0.873	-0.284	-0.250	57	92.92	0.12	-0.05	-0.06	115	5.41
3-Furanmethanol	FUR3OL	0.521	0.156	-0.153	138	46.31	0.07	0.03	-0.03	7	12.56
2,3-Dihydro-1,1,5,6-Tetramethyl-1H-Indene	DHT1156MIDNE	0.067	-0.938	0.054	68	91.23	0.01	-0.17	0.01	81	6.74
2-(2-Furanylmethyl)-5-Methyl-Furan	FURYLMFUR	0.891	0.239	-0.237	60	92.42	0.12	0.04	-0.06	99	6.01
5-Methyl-2-Furanmethanol	M5FUR2OL	0.874	-0.238	-0.098	80	88.58	0.12	-0.05	-0.02	59	7.98
Benzyl Acetate	PMESTAA	0.193	0.183	0.910	19	96.76	0.03	0.03	0.22	65	7.54
2(5H)-Furanone	FURONE	0.839	-0.240	0.005	107	81.85	0.11	-0.05	0.00	64	7.56
2,2'-Methylenebis[5-Methyl-Furan	MNEB5MFUR	0.840	0.273	-0.079	83	87.86	0.11	0.05	-0.02	74	7.04

1,2-Cyclopentanedione	CPT12DONE	0.838	-0.115	0.004	113	78.44	0.11	-0.02	0.00	55	8.18
2-Cyclohexenol	CHEX2E1OL	0.977	0.134	-0.077	13	97.78	0.13	0.02	-0.02	142	4.00
3-Methyl-1,2-Cyclopentanedione	M3CPT12DONE	0.895	-0.419	-0.039	1	99.15	0.12	-0.08	-0.01	144	3.93
2-Ethyl-Hexanoic Acid	E2HXNOIC	-0.219	-0.367	0.004	127	66.09	-0.03	-0.07	0.00	5	13.32
β-Damascenone	DAMSNONE	0.741	0.024	-0.313	109	80.57	0.10	0.00	-0.07	31	9.69
Ethyl Undecanoate	EESTUNDA	0.033	0.105	0.918	74	89.49	0.00	0.02	0.22	73	7.04
2-Methyl-Propanoic Acid	M2PPOICA	0.571	-0.786	0.146	15	97.30	0.08	-0.15	0.03	137	4.66
2,4,6-Trihydroxypyrimidine	THDXYPYMNE	0.823	0.277	-0.245	75	89.13	0.11	0.05	-0.06	107	5.74
Mequinol	MEQNOL	0.800	0.246	-0.098	86	87.21	0.11	0.04	-0.02	51	8.59
2,2-Diethyl-3-Methyl-Oxazolidine	DEMOXZDNE	0.857	0.215	0.171	69	91.08	0.11	0.04	0.04	93	6.54
Oxypurinol	OXYPUROL	0.947	0.198	-0.039	41	95.01	0.13	0.04	-0.01	103	5.90
Benzenemethanol	BENZMTOL	0.484	0.131	0.330	98	84.08	0.06	0.02	0.08	23	9.96
4-Methyl-2(5H)-Furanone	M4FUR2ONE	0.924	-0.264	-0.165	25	96.21	0.12	-0.05	-0.04	140	4.51
3-Ethyl-2-Hydroxy-2-Cyclopenten-1-one	E3H2C2PT1ONE	0.724	-0.574	-0.215	46	94.70	0.10	-0.11	-0.05	111	5.58
Benzeneethanol	BENZETOL	0.020	0.082	0.919	82	88.06	0.00	0.01	0.22	48	8.80
Acrylamide	ACRYLMDE	0.863	0.284	0.214	77	89.00	0.11	0.05	0.05	63	7.65
Erythritol	ERYTOL	0.630	-0.727	0.150	32	95.76	0.08	-0.14	0.04	119	5.26
2,3-Dimethyl-3-Pyrazolin-5-one	DM23PYZLONE	0.961	-0.127	-0.004	27	96.07	0.13	-0.02	0.00	138	4.59
Furfural Acetone	FURALTONE	0.977	0.079	-0.094	6	98.21	0.13	0.01	-0.02	143	3.94
Cyclotene	CYTENE	0.956	-0.063	-0.025	38	95.24	0.13	-0.01	0.00	117	5.33
2-Methyl-1,4-Benzenediol	M2BNZ14DIOL	0.864	0.165	-0.072	42	94.98	0.11	0.03	-0.01	82	6.68
Heptanoic Acid	HPTOIC	0.056	-0.979	0.038	9	98.08	0.01	-0.18	0.01	120	5.24
Maltol	MALTOL	0.901	0.272	0.233	33	95.67	0.12	0.05	0.06	126	5.09
2-Acetylpyrrole	ACTLPYROLE	0.804	0.289	-0.139	88	86.75	0.11	0.05	-0.03	78	6.86
2,5-Furandicarboxaldehyde	FUR25DIAL	0.920	0.260	-0.143	40	95.13	0.12	0.05	-0.03	135	4.82
2H-Pyran-2,6(3H)-Dione	PYR26DIONE	0.884	0.272	0.106	66	91.73	0.12	0.05	0.03	71	7.10
1-(2-Furanyl)-2-Hydroxy-Ethanone	H2FURYLONE	0.654	0.211	0.219	125	67.73	0.09	0.04	0.05	39	9.32
Phenol	PHEOL	0.819	-0.441	-0.111	63	91.98	0.11	-0.08	-0.03	100	5.95
1H-Pyrrole-2-Carboxaldehyde	PYRLE2AL	0.727	-0.593	-0.166	59	92.66	0.10	-0.11	-0.04	105	5.78
Phloroglucinol	PHLOGLNOL	0.913	0.189	0.233	43	94.82	0.12	0.03	0.06	130	4.96
Furaneol	FUREOL	0.627	-0.370	0.279	128	64.81	0.08	-0.07	0.06	24	9.95
Octanoic Acid	OCTOIC	0.159	-0.954	0.007	20	96.71	0.02	-0.18	0.00	118	5.33
5-Acetyl-Dihydro-2(3H)-Furanone	ACYLDHFURONE	0.828	-0.343	0.134	96	84.60	0.11	-0.06	0.03	68	7.26
2-Furanpropionic Acid	FURPPIONIC	0.927	0.244	-0.053	36	95.42	0.12	0.04	-0.01	121	5.24

1,3-Dihydroxy-2-Propanone	DHYPPAONE	-0.075	-0.292	0.297	115	75.70	-0.01	-0.06	0.07	13	11.74
Tetrahydro-5-Methyl-2-Furanmethanol	TEHYMFUROL	-0.414	-0.379	0.433	118	73.62	-0.06	-0.07	0.10	18	11.01
2-Hydroxy-Dihydro-2(3H)-Furanone	HY2DHFURONE	-0.511	-0.163	0.311	135	53.19	-0.07	-0.03	0.07	20	10.86
5-Acetoxymethyl-2-Furfural A	ACTYMFURALA	0.696	0.307	0.290	103	82.98	0.09	0.05	0.07	54	8.29
5-Acetoxymethyl-2-Furfural B	ACTYMFURALB	0.839	0.261	0.139	79	88.70	0.11	0.05	0.03	95	6.32
2-Methoxy-4-Vinyl-Phenol	MXY2VYL4PHEOL	0.942	0.121	-0.054	61	92.28	0.12	0.02	-0.01	133	4.89
3-Methyl-2,4(3H,5H)-Furandione	M3FURDIONE	0.985	0.081	-0.055	7	98.20	0.13	0.01	-0.01	141	4.42
3-Hydroxy-2,3-Dihydro-Maltol	HX3DH23MALTOL	0.100	-0.585	0.246	133	56.55	0.01	-0.11	0.06	10	12.00
Decanoic Acid	DECOIC	0.175	-0.957	0.079	17	97.17	0.02	-0.18	0.02	123	5.15
5-Hydroxy-Maltol	HX5MALTOL	0.912	0.126	-0.085	54	93.32	0.12	0.02	-0.02	69	7.20
2,3-Dihydro-Benzofuran	DHBNZFUR	0.840	0.029	-0.062	102	83.20	0.11	0.00	-0.01	94	6.37
1,4:3,6-Dianhydro-.alpha.-d-Glucopyranose	GLUPYROSE	0.750	-0.489	0.041	85	87.40	0.10	-0.09	0.01	62	7.66
4-Pyridinol	PYRDINOL	0.905	0.242	-0.258	22	96.57	0.12	0.04	-0.06	139	4.55
4-Amino-Phenol	AMIPHEOL	0.861	0.231	0.055	16	97.25	0.11	0.04	0.01	106	5.75
Benzoic Acid	BNZOIC	0.269	-0.930	0.056	8	98.18	0.04	-0.17	0.01	128	4.99
5-(Hydroxymethyl)-Dihydro-2(3H)-Furanone	HM5FURONE	0.703	0.083	0.055	132	60.18	0.09	0.01	0.01	36	9.56
Dodecanoic Acid	DODECOIC	0.748	-0.069	-0.056	131	61.80	0.10	-0.01	-0.01	25	9.95
5-(Hydroxymethyl)-2-Furfural	HM5FURAL	0.107	0.611	-0.141	123	69.81	0.01	0.11	-0.04	6	12.56
3-Methoxy-1,2-Benzenediol	M3BNZDIOL	0.890	0.219	-0.249	62	92.06	0.12	0.04	-0.06	114	5.45
1-(2-Furanyl)-1,2-Ethanediol	FURYLETDIOL	0.048	-0.264	0.282	121	71.33	0.01	-0.05	0.07	16	11.49
4-Hydroxy-Dihydro-2(3H)-Furanone	DHYHYFURONE	0.523	0.078	0.080	141	38.18	0.07	0.01	0.02	1	16.66

[1]VIP - Variable Importance in Projection scores.

Table S10. Loading results of samples and variables from Principal Component Analysis (PCA) and Partial Least Squares Analysis (PLS) based on the relative peak areas of the identified VOCs in sugarcane-based syrups samples.

ID Sample Code	ID Group Code	PCA			PLS		
		1	2	3	1	2	3
FRS07	CERT	0.01	0.07	-0.06	n.a.	n.a.	n.a.
FRS13	CERT	0.21	0.14	0.14	n.a.	n.a.	n.a.
FRS14	CERT	0.35	0.08	-0.06	n.a.	n.a.	n.a.
FRS15	CERT	0.53	0.10	-0.22	n.a.	n.a.	n.a.

FRS16	CERT	-0.08	0.10	-0.06	n.a.	n.a.	n.a.
FRS17	CERT	-0.03	0.10	-0.09	n.a.	n.a.	n.a.
FRS18	CERT	0.48	0.05	-0.18	n.a.	n.a.	n.a.
ECAL14	NCERT	-0.15	0.09	-0.06	n.a.	n.a.	n.a.
ENCAL14	NCERT	-0.32	0.08	-0.13	n.a.	n.a.	n.a.
GLA14	NCERT	0.07	0.12	0.94	n.a.	n.a.	n.a.
ESP16	MED	-0.23	0.02	-0.05	n.a.	n.a.	n.a.
EGP17	MED	-0.05	-0.01	0.06	n.a.	n.a.	n.a.
EGPA16	MED	-0.08	-0.01	0.01	n.a.	n.a.	n.a.
EGPB16	MED	-0.18	0.01	-0.04	n.a.	n.a.	n.a.
EGPC16	MED	-0.25	0.03	-0.06	n.a.	n.a.	n.a.
MCBR14	STH	-0.13	-0.04	0.00	n.a.	n.a.	n.a.
MDBR14	STH	0.07	-0.98	0.04	n.a.	n.a.	n.a.
AUS17	STH	-0.23	0.05	-0.17	n.a.	n.a.	n.a.
Centroid (CERT)	CERT	n.a.	n.a.	n.a.	0.09	0.06	-0.06
Centroid (NCERT)	NCERT	n.a.	n.a.	n.a.	-0.03	0.03	0.12
Centroid (MED)	MED	n.a.	n.a.	n.a.	-0.05	0.01	-0.01
Centroid (STH)	STH	n.a.	n.a.	n.a.	-0.02	-0.11	-0.02

n.a. – not available.

Table S11. Scores results of all cases from Principal Component Analysis (PCA) and Partial Least Squares Analysis (PLS) based on the relative peak areas of the identified VOCs in sugarcane-based syrups samples.

ID Replicate Number	ID Replicate Code	PCA			PLS		
		1	2	3	1	2	3
1	S1-15A	6.71	-0.90	0.26	6.73	-1.39	0.24
2	S1-15B	6.74	-1.30	0.38	6.76	-1.80	0.37
3	S1-15C	6.73	-1.10	0.32	6.74	-1.60	0.31
4	S1-16A	6.95	-3.41	0.22	6.97	-3.76	0.19
5	S1-16B	6.94	-3.38	0.20	6.96	-3.73	0.17
6	S1-16C	6.95	-3.40	0.21	6.96	-3.75	0.18
7	S1-17A	8.29	-12.36	1.66	8.31	-12.09	1.51
8	S1-17B	8.13	-11.06	1.53	8.15	-10.80	1.39

9	S1-17C	8.21	-11.71	1.60	8.23	-11.45	1.45
10	S1-18A	7.45	-10.41	-0.15	7.47	-10.29	-0.21
11	S1-18B	7.40	-9.25	-0.14	7.42	-9.14	-0.20
12	S1-18C	7.43	-9.83	-0.14	7.44	-9.71	-0.21
13	S2-15A	6.50	-0.20	0.59	6.51	-0.18	0.55
14	S2-15B	6.52	0.13	0.56	6.53	0.14	0.52
15	S2-15C	6.51	-0.04	0.57	6.52	-0.02	0.54
16	S2-16A	6.11	1.26	0.05	6.12	1.21	0.05
17	S2-16B	6.04	1.12	0.01	6.05	1.08	0.01
18	S2-16C	6.07	1.19	0.03	6.08	1.14	0.03
19	S2-17A	6.65	-0.04	0.46	6.66	0.01	0.43
20	S2-17B	6.64	0.00	0.44	6.64	0.05	0.41
21	S2-17C	6.65	-0.02	0.45	6.65	0.03	0.42
22	S2-18A	6.36	1.20	-0.09	6.37	1.17	-0.09
23	S2-18B	6.38	0.94	-0.10	6.38	0.91	-0.10
24	S2-18C	6.37	1.07	-0.10	6.37	1.04	-0.10
25	S3-15A	4.20	2.73	0.21	4.20	2.83	0.22
26	S3-15B	4.26	2.71	0.26	4.26	2.81	0.28
27	S3-15C	4.23	2.72	0.23	4.23	2.82	0.25
28	S3-16A	3.39	3.58	0.27	3.40	3.64	0.30
29	S3-16B	3.27	3.72	0.31	3.28	3.78	0.34
30	S3-16C	3.33	3.65	0.29	3.34	3.71	0.32
31	S3-17A	3.89	3.36	0.31	3.89	3.44	0.33
32	S3-17B	3.71	3.45	0.24	3.71	3.52	0.26
33	S3-17C	3.80	3.41	0.28	3.80	3.48	0.30
34	S3-18A	3.68	3.33	0.23	3.68	3.41	0.26
35	S3-18B	3.64	3.31	0.17	3.65	3.38	0.20
36	S3-18C	3.66	3.32	0.20	3.66	3.39	0.23
37	S4-15A	1.55	4.01	0.56	1.56	4.07	0.58
38	S4-15B	2.35	3.83	0.47	2.36	3.89	0.49
39	S4-15C	1.95	3.92	0.52	1.96	3.98	0.54
40	S4-16A	3.06	3.51	0.27	3.06	3.58	0.28
41	S4-16B	2.66	3.67	0.36	2.66	3.74	0.38
42	S4-16C	2.86	3.59	0.31	2.86	3.66	0.33

43	S4-17A	3.78	3.09	0.00	3.78	3.17	0.03
44	S4-17B	3.59	3.21	-0.06	3.58	3.30	-0.03
45	S4-17C	3.69	3.15	-0.03	3.68	3.23	0.00
46	S4-18A	3.18	3.48	-0.02	3.18	3.55	0.01
47	S4-18B	2.84	3.51	0.03	2.84	3.58	0.06
48	S4-18C	3.01	3.50	0.01	3.01	3.56	0.03
49	S5-15A	-9.18	1.46	-2.55	-9.32	1.28	-2.51
50	S5-15B	-10.59	1.31	-2.61	-10.72	1.13	-2.57
51	S5-15C	-9.89	1.38	-2.58	-10.02	1.20	-2.54
52	S5-16A	-20.09	-1.99	-12.39	-20.14	-1.90	-11.98
53	S5-16B	-17.36	-1.72	-11.78	-17.41	-1.63	-11.35
54	S5-16C	-18.72	-1.85	-12.08	-18.77	-1.76	-11.66
55	S5-17A	-25.33	-2.03	-1.50	-25.32	-1.99	-1.39
56	S5-17B	-24.97	-1.89	-1.81	-24.96	-1.85	-1.72
57	S5-17C	-25.15	-1.96	-1.66	-25.14	-1.92	-1.56
58	S5-18A	-25.18	-0.78	10.67	-25.13	-0.83	10.23
59	S5-18B	-30.16	-1.21	12.44	-30.10	-1.26	12.07
60	S5-18C	-27.67	-0.99	11.56	-27.61	-1.05	11.15

Table S12. Canonical Discriminant Function Coefficients and Highest Probability Classification results of samples from Linear Discriminant Analysis (LDA) after matrix reduction method obtained from One-way ANOVA test based on the relative peak areas of identified VOCs in sugarcane-based syrups samples.

ID Replicate Code	ID Group Code	LDA									PLS					
		CDF[1]			CDF[1] Class Means			Highest Probability Classification			Score Value			Loading Value		
		1	2	3	1	2	3	First	Second	Third	1	2	3	1	2	3
FRS07A	CERT	-367.3	-4.6	-2.0	-367.5	-4.8	-2.6	CERT	MED	STH	1.51	0.73	-0.09	0.18	0.04	0.02
FRS07B		-367.4	-5.2	-2.0				CERT	MED	STH	2.15	0.57	-0.21			
FRS07C		-367.4	-4.9	-2.0				CERT	MED	STH	1.83	0.65	-0.15			
FRS13A		-367.3	-4.8	-1.9				CERT	MED	STH	5.66	-0.03	-0.35			
FRS13B		-367.8	-4.9	-3.4				CERT	MED	STH	4.50	0.26	-0.17			
FRS13C		-367.6	-4.8	-2.6				CERT	MED	STH	5.08	0.11	-0.26			

FRS14A		-367.5	-4.6	-1.9				CERT	MED	STH	7.78	-0.58	-0.11			
FRS14B		-367.6	-5.0	-3.0				CERT	MED	STH	7.80	-0.45	-0.12			
FRS14C		-367.5	-4.8	-2.4				CERT	MED	STH	7.79	-0.52	-0.11			
FRS15A		-367.3	-5.5	-2.4				CERT	MED	STH	8.87	-0.55	0.01			
FRS15B		-367.7	-4.4	-3.0				CERT	MED	STH	11.10	-0.93	-0.08			
FRS15C		-367.5	-4.9	-2.7				CERT	MED	STH	9.99	-0.74	-0.04			
FRS16A		-366.7	-5.7	-3.4				CERT	MED	STH	0.38	1.23	0.48			
FRS16B		-368.4	-4.2	-2.1				CERT	MED	STH	0.84	1.16	0.46			
FRS16C		-367.6	-4.9	-2.8				CERT	MED	STH	0.61	1.19	0.47			
FRS17A		-367.6	-3.6	-1.4				CERT	MED	STH	0.85	1.01	0.35			
FRS17B		-367.5	-5.9	-4.7				CERT	MED	STH	1.72	0.82	0.33			
FRS17C		-367.6	-4.8	-3.1				CERT	MED	STH	1.28	0.92	0.34			
FRS18A		-367.8	-4.4	-2.4				CERT	MED	STH	8.01	0.13	0.20			
FRS18B		-367.4	-5.2	-2.8				CERT	MED	STH	10.18	-0.02	0.21			
FRS18C		-367.6	-4.8	-2.6				CERT	MED	STH	9.09	0.05	0.20			
ECAL14A		189.2	-31.1	43.8				NCERT	MED	STH	-2.31	2.92	3.48			
ECAL14B		189.0	-29.4	44.2				NCERT	MED	STH	-2.60	2.81	3.27			
ECAL14C		189.1	-30.2	44.0				NCERT	MED	STH	-2.46	2.87	3.37			
ENCAL14A		192.3	-27.6	44.2				NCERT	MED	STH	-4.85	2.48	2.10			
ENCAL14B	NCERT	185.6	-32.6	42.8	189.1	-30.2	43.7	NCERT	MED	STH	-4.83	2.47	2.10	-0.05	0.22	0.34
ENCAL14C		189.0	-30.1	43.5				NCERT	MED	STH	-4.84	2.48	2.10			
GLA14A		189.8	-31.3	43.4				NCERT	MED	STH	-0.56	0.93	0.43			
GLA14B		188.4	-29.3	44.0				NCERT	MED	STH	-0.87	0.88	0.40			
GLA14C		189.1	-30.3	43.7				NCERT	MED	STH	-0.71	0.91	0.41			
ESP16A		217.2	75.4	-5.2				MED	NCERT	STH	-3.88	1.07	-0.76			
ESP16B		217.2	74.8	-9.3				MED	NCERT	STH	-3.95	1.09	-0.70			
ESP16C		217.2	75.1	-7.2				MED	NCERT	STH	-3.91	1.08	-0.73			
EGP17A		219.3	71.9	-5.0				MED	NCERT	STH	-2.42	0.22	-2.38			
EGP17B	MED	215.2	77.7	-9.1	217.1	75.0	-7.0	MED	NCERT	STH	-2.17	-0.05	-2.50	-0.09	0.06	-0.50
EGP17C		217.3	74.8	-7.0				MED	NCERT	STH	-2.29	0.08	-2.44			
EGPA16A		216.9	76.2	-5.8				MED	NCERT	STH	-2.80	0.19	-2.65			
EGPA16B		216.8	74.7	-8.6				MED	NCERT	STH	-2.81	0.29	-2.95			
EGPA16C		216.8	75.4	-7.2				MED	NCERT	STH	-2.81	0.24	-2.80			
EGPB16A		217.8	74.2	-6.6				MED	NCERT	STH	-3.73	0.02	-2.77			

EGPB16B		216.7	75.8	-7.2				MED	NCERT	STH	-3.59	0.13	-2.60			
EGPB16C		217.3	75.0	-6.9				MED	NCERT	STH	-3.66	0.08	-2.69			
EGPC16A		219.7	75.3	-9.0				MED	NCERT	STH	-4.27	0.64	-1.69			
EGPC16B		214.6	73.8	-4.5				MED	NCERT	STH	-4.35	0.84	-1.51			
EGPC16C		217.2	74.6	-6.8				MED	NCERT	STH	-4.31	0.74	-1.60			
MCBR14A		306.0	-82.6	-25.8				STH	MED	NCERT	-3.24	-2.55	1.40			
MCBR14B		307.1	-84.4	-26.4				STH	MED	NCERT	-3.02	-2.69	1.27			
MCBR14C		306.6	-83.5	-26.1				STH	MED	NCERT	-3.13	-2.62	1.34			
MDBR14A		306.1	-82.4	-24.3				STH	MED	NCERT	-3.59	-7.09	1.31			
MDBR14B	STH	307.1	-84.6	-27.8	306.6	-83.4	-26.0	STH	MED	NCERT	-3.71	-6.96	1.22	-0.07	-0.35	0.23
MDBR14C		306.6	-83.5	-26.1				STH	MED	NCERT	-3.65	-7.02	1.27			
AUS17A		307.4	-84.6	-25.5				STH	MED	NCERT	-3.81	-0.56	1.32			
AUS17B		305.9	-82.0	-26.1				STH	MED	NCERT	-3.99	-0.45	1.33			
AUS17C		306.6	-83.3	-25.8				STH	MED	NCERT	-3.90	-0.50	1.33			

[1]CDF - Canonical Discriminant Function Coefficients.

Table S13. Information **s**ummary of Partial Least Square Analysis (PLS) based only on the relative peak areas of the most predictive VOCs identified in sugarcane-based syrups samples.

Analysis	Component	R^2X	Eigenvalues	R^2Y	Q^2	Significance	Iterations
PLS	1	0.6370	22.931	0.0585	0.0104	S	5
	2	0.1211	4.359	0.0588	0.0224	S	8
	3	0.0720	2.590	0.0588	0.0209	S	16
	4	0.0479	1.725	0.0585	0.0169	S	11
	5	0.0332	1.196	0.0580	0.0214	S	14
	6	0.0228	0.822	0.0581	0.0346	S	10
	7	0.0158	0.569	0.0584	0.0242	S	14
	8	0.0122	0.440	0.0582	0.0340	S	20
	9	0.0089	0.321	0.0579	0.0271	S	8
	10	0.0069	0.250	0.0581	0.0350	S	10
	11	0.0054	0.194	0.0561	0.0454	S	37
	12	0.0046	0.164	0.0578	0.0584	S	18

13	0.0035	0.126	0.0571	0.0721	S	18
14	0.0030	0.107	0.0569	0.1358	S	19
15	0.0023	0.083	0.0554	0.1979	S	8
16	0.0011	0.040	0.0551	0.3034	S	8
17	0.0005	0.017	0.0488	0.5225	S	5
18	0.0006	0.018	0.0060	0.0654	S	9

Table S14. Results of variables from Linear Discriminant Analysis (LDA) after matrix reduction method to 20 % of original dimension according with higher on the variable importance in projection (VIP) scores values from the PLS analysis based on the relative peak areas of the identified VOCs in sugarcane-based syrups samples.

Volatile Organic Compounds	Abbreviations	PLS		LDA	
		VIP[1]			
		Importance	Power (x 100)	W[2]	F[3]
4-Hydroxy-Dihydro-2(3H)-Furanone	DHYHYFURONE	1	16.66	3.65E-01	11.01
2,5-Dimethyl-Pyrazine	DM25PYZNE	2	16.06	1.08E-01	52.36
m-Menthane	MMTHANE	3	14.24	1.52E-01	35.32
3,5-Dimethyl-Dihydro-2-Furanone	DM35DHFURONE	4	14.09	7.00E-02	84.12
2-Ethyl-Hexanoic Acid	E2HXNOIC	5	13.32	1.74E-01	30.00
5-(Hydroxymethyl)-2-Furfural	HM5FURAL	6	12.56	9.88E-02	57.78
3-Furanmethanol	FUR3OL	7	12.56	3.37E-01	12.46
2-Heptanone	HPT2ONE	8	12.53	1.25E-01	44.35
Toluene	TOLNE	9	12.22	1.70E-01	30.92
3-Hydroxy-2,3-Dihydro-Maltol	HX3DH23MALTOL	10	12.00	5.06E-02	118.94
3-Methyl-Furfural	M3FURAL	11	11.78	5.51E-02	108.69
Hexane	HXANE	12	11.76	7.81E-02	74.72
1,3-Dihydroxy-2-Propanone	DHYPPAONE	13	11.74	1.39E-01	39.10
Dimethyl Disulfide	DMDSFD	14	11.58	Removed from analysis.	
Furan	FUR	15	11.50	5.16E-02	116.40
1-(2-Furanyl)-1,2-Ethanediol	FURYLETDIOL	16	11.49	1.52E-01	35.27
Ethyl Formate	EESTFA	17	11.06	9.00E-02	64.02

Tetrahydro-5-Methyl-2-Furanmethanol	TEHYMFUROL	18	11.01	2.45E-01	19.50
2,5-Dimethyl-Furan	DM25FUR	19	10.87	3.60E-01	11.28
2-Hydroxy-Dihydro-2(3H)-Furanone	HY2DHFURONE	20	10.86	5.74E-02	104.08
Pentane	PTANE	21	10.79	1.47E-01	36.65
1,3-Diazine	D13ZINE	22	9.98	2.12E-01	23.53
Benzenemethanol	BENZMTOL	23	9.96	7.13E-02	82.55
Furaneol	FUREOL	24	9.95	2.44E-01	19.66
Dodecanoic Acid	DODECOIC	25	9.95	4.00E-02	151.93
Propanoic Acid	PPANOIC	26	9.90	2.88E-01	15.69
2-Methyl-Furan	M2FUR	27	9.83	2.44E-01	19.67
2-Propyl-Furan	PP2FUR	28	9.81	1.35E-01	40.60
Nonanal	NONAL	29	9.71	1.69E-01	31.04
2-Propenal	PPENAL	30	9.70	1.16E-01	48.21
β-Damascenone	DAMSNONE	31	9.69	2.90E-01	15.50
Dimethyl Sulfide	DMSULFI	32	9.61	1.43E-01	38.09
Decanal	DECAL	33	9.61	1.45E-01	37.49

[1]F - Fischer value from One-way ANOVA test.

[2]W - Wilks value from Linear Discriminant Analysis.

[3]F - Fischer value from Linear Discriminant Analysis.

[4]CDF - Canonical Discriminant Function Coefficients.
